# Unravelling unexplored diversity of cercosporoid fungi (Mycosphaerellaceae, Mycosphaerellales, Ascomycota) in tropical Africa

**DOI:** 10.3897/mycokeys.81.67850

**Published:** 2021-06-17

**Authors:** Yalemwork Meswaet, Ralph Mangelsdorff, Nourou S. Yorou, Meike Piepenbring

**Affiliations:** 1 Department of Mycology, Institute of Ecology, Evolution and Diversity, Faculty of Biosciences, Goethe University Frankfurt am Main, Biologicum, Max-von-Laue-Str. 13, 60438 Frankfurt am Main, Germany Goethe University Frankfurt am Main Frankfurt am Main Germany; 2 Faculty of Agronomy, University of Parakou, BP 123 Parakou, Benin University of Parakou Parakou Benin

**Keywords:** Benin, *
Cercospora
*, Fabaceae, Leguminosae, molecular phylogenetic analysis, *
Nothopassalora
*, *
Passalora
*, *
Pseudocercospora
*, West Africa

## Abstract

Cercosporoid fungi (Mycosphaerellaceae, Mycosphaerellales, Ascomycota) are one of the largest and most diverse groups of hyphomycetes causing a wide range of diseases of economically important plants as well as of plants in the wild. Although more than 6000 species are known for this group, the documentation of this fungal group is far from complete. Especially in the tropics, the diversity of cercosporoid fungi is poorly known. The present study aims to identify and characterise cercosporoid fungi collected on host plants belonging to Fabaceae in Benin, West Africa. Information on their morphology, host species and DNA sequence data (18S rDNA, 28S rDNA, ITS and *tef1*) is provided. DNA sequence data were obtained by a simple and non-culture-based method for DNA isolation which has been applied for cercosporoid fungi for the first time in the context of the present study. Among the loci used for the phylogenetic analysis, *tef1* provided the best resolution together with the multigene dataset. Species delimitation in many cases, however, was only possible by combining molecular sequence data with morphological characteristics. Based on forty specimens recently collected in Benin, 18 species are presented with morphological descriptions, illustrations and sequence data. Among these, six species in the genus *Cercospora* and two species in *Pseudocercospora* are proposed as species new to science. The newly described species are Cercospora (C.) beninensis on *Crotalaria macrocalyx*, *C. parakouensis* on *Desmodium tortuosum*, *C. rhynchophora* on *Vigna unguiculata*, *C. vignae*-*subterraneae* on *Vigna subterranea*, *C. tentaculifera* on *Vigna unguiculata*, *C. zorniicola* on *Zornia glochidiata*, *Pseudocercospora sennicola* on *Senna occidentalis* and *Pseudocercospora tabei* on *Vigna unguiculata*. Eight species of cercosporoid fungi are reported for Benin for the first time, three of them, namely C. cf. canscorina, C. cf. fagopyri and *C. phaseoli-lunati* are new for West Africa. The presence of two species of cercosporoid fungi on Fabaceae previously reported from Benin, namely *Nothopassalora personata* and *Passalora arachidicola*, is confirmed.

## Introduction

Hyphomycetous anamorphs of *Mycosphaerella*-like teleomorphs are generally referred to as cercosporoid fungi and are classified in genera with concepts that often changed (Crous and Braun 2003; Braun et al. 2013; Kirschner 2014). Cercosporoid fungi include about 6000 recognized species (Braun et al. 2015), in more than ten genera, with *Cercospora* Fresen. (*C.*), *Nothopassalora* U.Braun, C. Nakash., Videira & Crous (*N.*), *Passalora* Fr. (*P.*) and *Pseudocercospora* Speg. (*Ps.*) being the genera relevant for the present publication.

Cercosporoid fungi belonging to Mycosphaerellaceae (Mycosphaerellales, Ascomycota) are one of the largest and most diverse groups of hyphomycetes and cause a wide range of diseases, on numerous economically important plants such as cereals, vegetables and fruits as well as on wild plants. Major diseases include the angular leaf spot of bean caused by *Pseudocercospora griseola*, black leaf streak of banana caused by *Ps. fijiensis* (M.Morelet) Deighton, fruit and leaf spot disease of citrus caused by *Ps. angolensis* (T.Carvalho & O.Mendes) Crous & U.Braun, leaf spot disease of celery (*Cercospora apii* Fresen.), of sugar beet (*C. beticola* Sacc.), and foliar diseases of groundnut caused by *Nothopassalora personata* (Berk. & M.A.Curtis) U.Braun, C. Nakash., Videira & Crous or *Passalora arachidicola* (Hori) U.Braun (Braun et al. 2013; Videira et al. 2017). Infections by these fungi are mostly evident by leaf spots, but cercosporoid fungi can also cause necrotic lesions on flowers, fruits, seeds and pedicels of numerous hosts in most climatic regions (Agrios 2005). Cercosporoid fungi are known from all parts of the world but they are more abundant and diverse in tropical and subtropical regions (Beilharz et al. 2002; Braun and Freire 2004; Hernández-Gutiérrez and Dianese 2008, 2009).

Cercosporoid fungi are dematiaceous hyphomycetes with conidiophores formed singly or in groups, arranged in sporodochia or in synnemata, with integrated, terminal or intercalary conidiogenous cells (Crous and Braun 2003; Ávila et al. 2005; Braun et al. 2013). Most of the cercosporoid species were previously assigned to a single genus, *Cercospora*, which was later split into several smaller genera mainly by Deighton (1967, 1973, 1974, 1976, 1979), Braun (1993) and Crous and Braun (2003). Crous and Braun (2003) recognized four genera, namely *Cercospora*, *Passalora*, *Pseudocercospora* and *Stenella* Syd as important cercosporoid genera. Later, the genus *Stenella* was assigned to the Teratosphaeriaceae based on the phylogenetic placement of the type species. *Stenella*-like species remaining in Mycosphaerellaceae were classified in the genus *Zasmidium* Fr. (Arzanlou et al. 2007; Braun et al. 2013). In the present paper, we follow generic concepts defined by Crous and Braun (2003) and recently updated by Braun et al. (2013), Crous et al. (2013a), Groenewald et al. (2013) and Videira et al. (2017). However, according to recent molecular sequence analyses, most genera of the cercosporoid fungi are not monophyletic (Videira et al. 2017). As many cercosporoid fungi have a strong impact on cultivated plants, a better understanding and stabilisation of the taxonomy of these fungi are urgently needed.

The genus *Cercospora* was established by Fresenius in 1863 (Fuckel 1863) based on the type species *Cercospora apii* (Braun and Crous 2016; Videira et al. 2017). It is one of the most species-rich genera of the hyphomycetes and contains numerous important plant pathogenic fungi throughout the world (Crous and Braun 2003). In 1954, the genus was monographed by Chupp (1954), who treated 1419 *Cercospora*-species using a broad generic concept. Later, several attempts have been made to split *Cercospora* s. lat. into smaller genera by using characteristics of conidiomatal structure, hyphae, conidiophores, conidiogenous cells, conidiogenous loci and conidia (Ellis 1971, 1976; Deighton 1973, 1979, 1983; Braun 1995a, 1998; Crous and Braun 2003). Currently, *Cercospora* species are morphologically characterised by pigmented conidiophores, unpigmented conidia, as well as thickened and darkened conidiogenous loci and conidial hila (Crous and Braun 2003; Groenewald et al. 2013). A significant problem in the taxonomy of *Cercospora* is the host specificity of its species. Most *Cercospora* species are considered to be distinct based on the host and thus assumed to be specific to a host species or to a host genus (Chupp 1954; Braun 1995a). Some species, such as *C. apii* and *C. beticola*, however, were isolated from a high number of host species belonging to several families (Groenewald M et al. 2006). Moreover, phylogenetic approaches based on multi-locus sequences can be problematic for species delimitation in *Cercospora* due to a high level of conservation in DNA sequences of commonly used loci (i.e., ITS, *tef1*, actA, cmdA and his3) (Bakhshi et al. 2018).

The genus *Pseudocercospora* was introduced by Spegazzini (1910) based on the type species *Ps. vitis* (Lév.) Speg., a foliar pathogen of grapevine. The majority of *Pseudocercospora* species are known as pathogens occurring on many different plants, mainly in tropical and sub-tropical regions (Chupp 1954; Crous and Braun 2003; Crous et al. 2013). In contrast to *Cercospora* spp., they are characterised by pigmented conidiophores and conidia, without thickened and darkened conidiogenous loci and conidial hila (Deighton 1976). The monophyly of the genus has not yet been fully resolved (Kirschner 2014). According to molecular sequence data, most species of *Pseudocercospora* appear to be host specific (Crous et al. 2013).

The genus *Passalora* Fr. was introduced by Fries (1849) based on the type species *Passalora bacilligera* (Mont. & Fr.) Mont. & Fr. (≡ *Cladosporium bacilligerum* Mont. & Fr.) (Videira et al. 2017). Species of *Passalora* are characterised by pigmented conidiophores and conidia as well as thickened and darkened conidiogenous loci and conidial hila (Crous and Braun 2003).

Several molecular phylogenetic studies are available on species of cercosporoid fungi that are represented by strains in culture collections (Świderska-Burek et al. 2020). These, however, only represent a small fraction of several hundreds of taxa of cercosporoid fungi that are valid species defined by morphological characteristics (Braun et al. 2016; Świderska-Burek et al. 2020). Therefore, the number of cercosporoid species known by detailed morphological characteristics as well as molecular sequence data has to be increased.

Although cercosporoid fungi cause a wide range of diseases on major agricultural crops, the study of cercosporoid fungi in West Africa is still at an early pioneer stage and only very incomplete information is currently available (Piepenbring et al. 2020). To date, approximately 320 species of cercosporoid hyphomycetes are known from 14 West African countries (Piepenbring et al. 2020, Suppl. materials 1, 2). Among these, 12 species of cercosporoid fungi have been reported for Benin (Turner 1971; Marley et al. 2002; Crous and Braun 2003; Houessou et al. 2011; Piątek and Yorou 2018; Soura et al. 2018; Meswaet et al. 2019; Farr and Rossman 2021). Morphological characteristics and molecular sequence data are lacking for most cercosporoid species known for Benin and other West African countries. Although cercosporoid fungi have been investigated for more than 150 years and are important in the agricultural sector, almost no, or only inadequate, studies have been carried out in most West African countries such as Benin. In addition to this lack of species knowledge in tropical regions, many species of cercosporoid fungi are characterised morphologically only. Since many cercosporoid species are known as pathogens on cultivated plants, an accurate diagnosis, identification and documentation of these fungi are a prerequisite and urgent for their control and epidemiological surveys.

As a first step towards a systematic documentation of cercosporoid fungi in tropical Africa, we focus on species infecting hosts belonging to the Fabaceae (Leguminosae) in the present publication. Fabaceae are the third largest family of angiosperms (Gepts et al. 2005). This family includes peas, lentils, beans, peanuts and other plants with pods and/or seeds that are consumed as food (Messina 1999). Several species belonging to *Vigna* originate from West Africa (Benin, Burkina Faso, Cameroon, Ghana, Niger, Nigeria and Togo) including two important cultivated crops *Vigna unguiculata* (L.) Walp. and *Vigna subterranea* (L.) Verdc. (Hepper 1963; Faris 1965; Padulosi and Ng 1990). They provide important nutrients such as proteins, low glycemic index carbohydrates, minerals and vitamins. Legumes are richer in protein than other cultivated plants because of nitrogen-fixing bacteria living in nodules of their roots (Kouris-Blazos and Belski 2016).

We apply an integrative approach that includes sampling in Benin, detailed descriptions and illustrations of collected specimens and herbarium specimens, examination of closely related known species on the same or closely related host species based on herbarium specimens and the isolation, sequencing and analysis of nuclear DNA sequence data. For the isolation of DNA, a new, simple method for DNA isolation has been developed and is presented for the first time for cercosporoid fungi.

## Methods

### Collections and morphological studies

Samples of leaves infected by cercosporoid fungi were randomly collected in farmlands and fallows in Benin from July–August 2016, July–September 2017 and August–September 2019. Infected leaves were dried in a plant press and deposited in the herbaria Botanische Staatssammlung München (M) and University of Parakou (**UNIPAR**).

Dried specimens were observed by stereomicroscopy and by light microscopy, using a Zeiss Axioscope 40 microscope. For light microscopy, leaf sections were made with razor blades and mounted in distilled water or 5% KOH without staining. Semi-permanent preparations of sections of the infected leaves were made by a microtome (Leica CM 1510-1) and mounted in lactophenol with cotton blue. For approximately 50 ml lactophenol cotton blue solution we mixed 10 mg phenol, 0.025 mg cotton blue, 10 ml lactic acid, 20 ml glycerin and 10 ml distilled water. Measurements of 30 conidia, conidiophores and other structures have been made for each specimen at a magnification of ×1000. Measurements are presented as mean value ± standard deviation with extreme values in parentheses. Line drawings were made freehand on scaled paper. Images and drawings were edited with Photoshop CS5 (Adobe, San Jose, California). Critical taxa were determined with the help of type specimens and other specimens loaned from the US National Fungus Collections (BPI), the Herbarium of the University of Illinois (ILL) and the New York Botanical Garden (NY).

### Host plant identification

Host plants were identified by morphological characteristics and in some cases by molecular methods. Morphological identifications were made by comparison with herbarium specimens, literature (e.g., Akoégninou et al. 2006) and with the help of local botanists. Molecular sequence data for species identifications were obtained by polymerase chain reaction (PCR) for the amplification of the partial region of chloroplast rbcL with the primer pairs rbcLa-F (Levin et al. 2003) and rbcLa-R (Kress et al. 2009). DNA was extracted from approx. 0.05 g of leaf tissue dried with silica gel using the innuPREP Plant DNA Kit (Analytik Jena, Germany) and following the manufacturer’s instructions. Protocols for PCR were carried out as described by Fazekas et al. (2012).

### DNA Extraction and PCR amplification of fungal DNA

DNA was isolated from caespituli taken with a needle from dry specimens using the E.Z.N.A Forensic DNA Extraction Kit following the manufacturer’s instructions. Small pieces of leaves containing several clean caespituli, with as little contaminations as possible, were selected under the stereomicroscope. Precautions were taken to avoid picking cells of any other organism (fungi, algae) associated with the leaves. To extract total genomic DNA from caespituli, a small amount of clean hyphae from the leaf surface was transferred into a sterile Eppendorf tube using a sterilized needle or adhesive mini-tapes. The sample was homogenized for 7–10 min. using a Retsch Mixer Mill MM301 with TL buffer and 2.5 mm Zirconia beads. Isolated DNA was re-suspended in elution buffer and stored at -20 °C. DNA concentration was checked by a NanoDrop 2000c spectrophotometer (Thermo Fisher Scientific, USA).

Four partial nuclear gene regions (three ribosomal loci and one protein-coding gene) were amplified and sequenced: For the large subunit nuclear ribosomal DNA (nrLSU, 28S rDNA) the primers LSU1Fd and LSU3Rd (Crous et al. 2009a), for the small subunit nuclear ribosomal DNA (nrSSU, 18S rDNA) the primers SSU1Fd and SSU1Fd (Crous et al. 2009a), for the internal transcribed spacer region of ribosomal DNA (ITS) the primers V9G (de Hoog and van den Ende 1998) and ITS4 (White et al. 1990) and for the translation elongation factor 1-α (*tef1*) the primers EF1- 728F and EF1-986R (Carbone and Kohn 1999) were used. PCR amplification and sequencing were conducted following the protocols of Hunter et al. (2006), Crous et al. (2009a, 2012) and Videira et al. (2017). The PCR mixtures consisted of 1 μL genomic DNA, 15× MgCl_2_ reaction buffer (Bioline, Luckenwalde, Germany), 25 mM MgCl_2_, 25 μM of each dNTP, 10 μM of each primer and 5 U Taq DNA polymerase (VWR) in a total volume of 25 μL. Cycling parameters of the PCR for LSU, SSU and ITS were as follows: initial denaturation at 94 °C for 3 min, followed by 35 cycles of amplification [denaturation at 94 °C for 30 s, primer annealing at 52 °C for 30 s and primer extension at 72 °C for 45 s] and a final extension at 72 °C for 5 min, followed by storage at 8 °C. The PCR mixture for *tef1* contained 2 μL of template DNA and the cycling parameters to obtain the partial *tef1* were as follows: an initial denaturation at 96 °C for 2 min; followed by 35 cycles of amplification [denaturation at 94 °C for 30 s, primer annealing at 56 °C for 30 s and primer extension at 72 °C for 30 s] and a final extension at 72 °C for 7 min, followed by storage at 8 °C. PCR-products were checked on 1.5% agarose electrophoresis gels containing HDGreenPlus DNA stain. Ampliﬁed PCR products were puriﬁed with the Cycle Pure Kit (VWR-Omega, USA). Sequencing was performed at Seqlab GmbH, Germany.

### Molecular phylogeny

Amplification of the SSU, LSU, ITS and *tef1* gene regions for all isolates used in this study yielded fragments of approximately 1100 bp, 900 bp, 650 bp and 300 bp, respectively. Consensus sequences of trace files were generated with Geneious 10.2.2 (https://www.geneious.com, Kearse et al. 2012) and searched against GenBank (https://www.ncbi.nlm.nih.gov/, Benson et al. 2014) with MegaBLAST. Sequences with a high similarity (65 sequences of LSU, ITS and *tef1* regions) were retrieved (Table 1). A total of 148 sequences for 65 specimens were obtained from GenBank (Table 1) and 92 sequences for 28 specimens from Benin were generated in this study (Table 2). They were aligned with MAFFT v. 7 using the L-INS-i algorithm (Nakamura et al. 2018). The alignments were manually checked by using MEGA v. 7 (Kumar et al. 2016). Gblocks v. 0.91b (Talavera and Castresana 2007) was used to remove poorly aligned positions and divergent regions from the DNA alignment using the parameters for a less stringent selection. Subsequently, a four-locus concatenated alignment (SSU, LSU, ITS and *tef1*) dataset was assembled for phylogenetic analyses using Geneious 10.2.2. *Cladosporium sphaerospermum* (G402) served as outgroup taxon, because the genus *Cladosporium* s. str. was shown to be the sister group of *Mycosphaerella* s. str. (Braun et al. 2003). PartitionFinder2 v.2.1.1 (Lanfear et al. 2014) on XSEDE (Miller et al. 2010) was used to select the best-fit model of evolution for each gene fragment separately. Data were partitioned by gene and by codon position in the case of protein-coding sequences. The TRNEF+G model was applied to 28S rDNA, K80 model to 18S rDNA, K81+G to ITS and TRN+G model to *tef1*. The alignment and the tree were deposited in TreeBASE (http://purl.org/phylo/treebase/phylows/study/TB2:S28032). Phylogenetic analyses of this study were conducted by applying Maximum Likelihood (ML) in RAxML-HPC2 v.8.2.12 (Stamatakis 2014) on XSEDE (Miller et al. 2010) and Bayesian with the program MrBayes 3.2.6 (Ronquist et al. 2012) on XSEDE (Miller et al. 2010) on the CIPRES Science Gateway web portal. (http://www. phylo.org/sub_sections/portal/).

For Maximum Likelihood analyses one thousand nonparametric bootstrap iterations were used with the generalised time-reversible model with a discrete gamma distribution (GTRGAMMA) (Stamatakis et al. 2008). For Bayesian phylogenies, two parallel runs with eight chains of Metropolis-coupled Markov chain Monte Carlo iterations were performed with the heat parameter being set at 0.2. Analyses were run for 100 million generations, with trees sampled every 1000^th^ generation until the average standard deviation of split frequencies reached 0.01 (stop value). The first 25% of saved trees were discarded as the ‘burn-in’ phase. Posterior probabilities (PP) were determined from the remaining trees. Bayesian posterior probabilities (BPP) ≥ 94% and Bootstrap values (BS) ≥ 70% are considered as significant.

**Table 1.** Data of DNA sequences of cercosporoid fungi downloaded from GenBank and used in this study.

Species	Host	Host family	Country	Source	GenBank Accession Numbers			Reference
					nrLSU	ITS	*tef1*	
Cercospora cf. apii Fresen.	*Cajanus cajan* (L.) Millsp.	Fabaceae	S. Africa	CBS 115411	JN941171	JN942278	–	Groenewald JZ et al. (2013)
*Cercospora asparagi* Sacc.	*Asparagus* sp.	Asparagaceae	USA	AS16-02	KY549100	KY549098	KY549102	Hay et al. (2017)
*Cercospora canescens* Ellis & G.Martin	*Vigna radiata* (L.) R.Wilczek.	Fabaceae	India	Cer70-18	–	MN795675	–	Das et al. 2019
*Cercospora capsica* Heald & F.A.Wolf	*Capsicum annuum* L.	Solanaceae	S. Korea	CBS 132622	–	JX143568	JX143323	Groenewald JZ et al. (2013)
Cercospora cf. citrulline Cooke	*Citrullus lanatus* (Thunb.) Matsum. & Nakai	Cucurbitaceae	Japan	MUCC 576	–	JX143579	JX143337	Groenewald JZ et al. (2013)
*Cercospora dubia* Speg.	*Chenopodium* sp.	Amaranthaceae	Mexico	CPC 15600	KX286968	KX287277	–	Videira et al. (2016)
*Cercospora kikuchii* (Tak. Matsumoto & Tomoy.) M.W.Gardner	*Glycine max* (L.) Merr.	Fabaceae	USA	DLS5070-3A		AY373573	AY373582	Cai and Schneider (2008)
*Cercospora lactucae*-*sativae* Sawada	*Lactuca sativa* L.	Asteraceae	Japan	MUCC 570	–	JX143623	JX143382	Groenewald JZ et al. (2013)
*Cercospora malayensis* F.Stevens & Solheim	*Abelmoschus esculentus* (L.) Moench	Malvaceae	S. Korea	KACC 47769	–	MH129519	MH129517	Ju et al. (2020)
Cercospora cf. maloti Ellis & Everh.	*Cucumis melo* L.	Cucurbitaceae	Japan	MUCC 575	–	JX143625	JX143384	Groenewald JZ et al. (2013)
Cercospora cf. nicotianae Ellis & Everh.	*Nicotiana tabacum* L.	Solanaceae	–	CBS 570.69	–	DQ835074	DQ835100	Groenewald JZ et al. (2010)
*Cercospora olivascens* Sacc.	*Aristolochia clematitis* L.	Aristolochiaceae	Romania	CBS 253.67	–	JX143632	JX143391	Groenewald JZ et al. (2013)
*Cercospora physalidis* Ellis	*Solanum melongena* L.	Solanaceae	India	Cer 69-18	MK027095	MK029358	–	Sinha et al., unpublished
*Cercospora rodmanii* Conway	*Eichhornia* sp.	Pontederiaceae	Mexico	15-GTOX	GQ884187	GQ884185	–	Montenegro-Calderón et al. (2011)
*Cercospora sojina* Hara	*Glycine soja* Siebold & Zucc.	Fabaceae	S. Korea	CBS 132615	–	JX143659	JX143419	Groenewald JZ et al. (2013)
*Cercospora* sp. Q JZG-2013	*Acacia mangium* Willd.	Fabaceae	Thailand	CPC 10550	–	AY752139	AY752172	Groenewald JZ et al. (2013)
*Cercospora vignigena* C. Nakash., Crous, U.Braun & H.D.Shin	*Vigna unguiculata* (L.) Walp.	Fabaceae	Japan	MUCC 579	–	JX143736	JX143495	Groenewald JZ et al. (2013)
*Cercospora zebrina* Pass.	*Trifolium subterraneum* L.	Fabaceae	Australia	CBS 118790	KF251651	KF251147	–	Quaedvlieg et al. (2013)
*Cladosporium sphaerospermum* Penz.	–	–	Russia	G402	KJ443113	KJ443245	KJ443201	Grum-Grzhimaylo et al. (2016)
*Mycosphaerella keniensis* Crous & T.A.Cout.	*Eucalyptus grandis* W.Hill	Myrtaceae	Kenya	CMW5147	DQ246259	–	DQ235100	Hunter et al. (2006)
*Mycosphaerella microsora* Syd.	*Tilia platyphyllos* Scop.	Malvaceae	Romania	CBS 552.71	MH872022	MH860260		Vu et al. (2019)
*Mycosphaerella valgourgensis* Crous	*Yucca* sp.	Asparagaceae	France	CPC:18385	JF951175	JF951152	–	Crous et al. (2011)
*Nothopassalora personata* (Berk. & M.A.Curtis) U.Braun, C. Nakash., Videira & Crous	*Arachis hypogaea* L.	Fabaceae	Australia	CBS 142236	NG_058496	NR_156379	–	Videira et al. (2017)
*Paracercospora egenula* (Syd.) Deighton	*Solanum melongena* L.	Solanaceae	India	CBS 485.81	JQ324940	GU269699	GU384415	Crous et al. (2013a)
*Passalora arctostaphyli* Moreno-Rico & Crous	*Arctostaphylos pungens* Kunth	Ericaceae	Mexico	CPC 22067	KJ152785	KJ152782	–	Moreno-Rico et al. (2014)
*Neocercosporidium smilacis* (Thüm.) U.Braun, C. Nakash., Videira & Crous	*Smilax aspera* L.	Smilacaceae	Italy	CBS 556.71	KJ633269	KJ633265	–	Collemare et al. (2015)
*Pseudocercospora abelmoschi* (Ellis & Everh.) Deighton	*Hibiscus syriacus* L.	Malvaceae	S.Korea	CBS 132103	GU253696	GU269647	GU384365	Crous et al. (2013a)
*Pseudocercospora atromarginalis* (G.F.Atk.) Deighton	*Solanum* sp.	Solanaceae	New Zealand	CBS 114640	GU253706	GU269658	GU384376	Crous et al. (2013a)
*Pseudocercospora cercidicola* Crous, U.Braun & C. Nakash.	*Cercis chinensis* Bunge	Fabaceae	Japan	MUCC 896	GU253719	GU269671	GU384388	Crous et al. (2013a)
*Pseudocercospora chengtuensis* (F.L.Tai) Deighton	*Lycium chinense* Mill.	Solanaceae	S. Korea	CBS 131924	MH877506	MH866053	–	Vu et al. (2019)
*Pseudocercospora chiangmaiensis* Cheew., K.D.Hyde & Crous	*Eucalyptus camaldulensis* Dehnh.	Myrtaceae	Thailand	CBS 123244	MH874812	MH863288	–	Vu et al. (2019)
*Pseudocercospora cruenta* (Sacc.) Deighton	*Phaseolus vulgaris* L.	Fabaceae	Taiwan	CBS 117232	GU253730	GU269689	GU384405	Crous et al. (2013a)
*Pseudocercospora cydoniae* (Ellis & Everh.) Y.L.Guo & X.J.Liu	*Chaenomeles speciosa* (Sweet) Nakai	Rosaceae	S. Korea	CBS 131923	MH877505	MH866052	–	Vu et al. (2019)
*Pseudocercospora dingleyae* U.Braun & C.F.Hill	*Haloragis erecta* (Murray) Oken	Haloragaceae	New Zealand	CBS 114645	KX286997	KX287299	–	Videira et al. (2016)
*Pseudocercospora dovyalidis* (Chupp & Doidge) Deighton	*Dovyalis zeyheri* (Sond.) Warb.	Salicaceae	S. Africa	CBS 126002	MH875338	MH863877	–	Vu et al. (2019)
*Pseudocercospora encephalarti* Y.Meswaet, Mangelsdorff, Yorou & M.Piepenbr.	*Encephalartos barteri* Carruth. ex Miq.	Zamiaceae	Benin	YMMAS78	–	MK397016	–	Meswaet et al. (2019)
*Pseudocercospora flavomarginata* G.C.Hunter, Crous & M.J.Wingf.	*Eucalyptus camaldulensis* Dehnh.	Myrtaceae	Thailand	CBS 118824	–	NR_111805	–	Quaedvlieg et al. (2012)
*Pseudocercospora fuligena* (Roldan) Deighton	*Solanum lycopersicum* L.	Solanaceae	Japan	MUCC 533	GU253749	GU269712	GU384428	Crous et al. (2013a)
*Pseudocercospora griseola f. griseola* (Sacc.) Crous & U.Braun	*Phaseolus vulgaris* L.	Fabaceae	S. Korea	CBS 131929	MH877495	MH866046	–	Vu et al. (2019)
*Pseudocercospora hakeae* (U.Braun & Crous) U.Braun & Crous	*Hakea* sp.	Proteaceae	Australia	CBS:144520	MK442553	MK442617	MK442708	Crous et al. (2019)
*Pseudocercospora humuli* (Hori) Y.L.Guo & X.J.Liu	*Humulus lupulus* L.	Cannabaceae	Japan	MUCC 742	GU253758	–	GU384439	Crous et al. (2013a)
*Pseudocercospora kaki* Goh & W.H.Hsieh	*Diospyros kaki* L.f.	Ebenaceae	Japan	MUCC 900	GU253761	GU269729	GU384442	Crous et al. (2013a)
*Pseudocercospora madagascariensis* Crous & M.J.Wingf.	*Eucalyptus camaldulensis* Dehnh.	Myrtaceae	Madagascar	CBS 124155	MH874880	MH863357	–	Vu et al. (2019)
*Pseudocercospora metrosideri* U.Braun	*Metrosideros collina* (J.R.Forst. & G.Forst.) A.Gray	Myrtaceae	New Zealand	CBS 118795	GU253774	GU269746	GU384458	Crous et al. (2013a)
*Pseudocercospora neriicola* Crous, Frisullo & Camele	*Nerium oleander* L.	Apocynaceae	Italy	CPC 23765	KJ869222	KJ869165	KJ869240	Crous et al. (2014)
*Pseudocercospora pallida* (Ellis & Everh.) H.D.Shin & U.Braun	*Campsis grandiflora* (Thunb.) K.Schum.	Bignoniaceae	S. Korea	CBS 131889	–	–	GU384469	Crous et al. (2013a)
*Pseudocercospora paraguayensis* (Tak. Kobay.) Crous	*Eucalyptus nitens* (H.Deane & Maiden) Maiden	Myrtaceae	Brazil	CBS:111286	KF901945	KF901619	KF903205	Quaedvlieg et al. (2014)
*Pseudocercospora parapseudarthriae* Crous & A.R.Wood	*Pseudarthria hookeri* Wight & Arn.	Fabaceae	S. Africa	CPC 23449	KJ869208	KJ869151	KJ869238	Crous et al. (2014)
*Pseudocercospora pittospori* (Plakidas) Y.L.Guo & X.J.Liu	*Pittosporum* sp.	Pittosporaceae	USA	HI-018	MK210475	MK210511	–	Vaghefi et al. 2021
*Pseudocercospora proteae* Crous	*Protea mundii* Klotzsch	Proteaceae	S. Africa	CBS 131587	–	–	GU384519	Crous et al. (2013a)
*Pseudocercospora prunicola* (Ellis & Everh.) U.Braun	*Prunus* sp.	Rosaceae	China	BJFU ZYP141005.9	KX853057	KX853048	KX853066	Liu et al. (2016)
*Pseudocercospora ranjita* (S.Chowdhury) Deighton	*Gmelina* sp.	Lamiaceae	Indonesia	CBS 126005	MH875340	MH863879	GU384500	Crous et al. (2013a)
*Pseudocercospora ravenalicola* G.C.Hunter & Crous	*Ravenala madagascariensis* Sonn.	Strelitziaceae	India	CBS 122468	GU253828	–	GU384521	Crous et al. (2013a)
*Pseudocercospora schizolobii* (M.J.Wingf. & Crous) M.J.Wingf. & Crous	*Eucalyptus camaldulensis* Dehnh.	Myrtaceae	Thailand	CBS 124990	KF251827	KF251323	KF253270	Verkley et al. (2013)
*Pseudocercospora sennae-multijugae* Meir. Silva, R.W.Barreto & Crous	*Senna multijuga* (Rich.) H.S.Irwin & Barneby	(Fabaceae)	Brazil	CPC 25206	KT290169	KT290142	KT290196	Silva et al. (2016)
*Pseudocercospora* sp.	*Citrus grandis* (L.) Osbeck	Rutaceae	China	ZJUM 75	KP895896	KP896026	KP896073	Huang et al. (2015)
*Pseudocercospora* sp.	*Eichhornia azurea* (Sw.) Kunth	Pontederiaceae	Brazil	CPC 19537	KX287003	KX287304	–	Videira et al. (2016)
*Pseudocercospora* sp.	*Eichhornia azurea* (Sw.) Kunth	Pontederiaceae	Brazil	CPC 19535	KX287001	KX287303	–	Videira et al. (2016)
*Pseudocercospora* sp. A MB-2015	*Phaseolus vulgaris* L.	Fabaceae	Iran	CCTU 1166	KP717028	KM452864	KM452886	Bakhshi et al. (2014)
*Pseudocercospora stizolobii* (Syd. & P.Syd.) Deighton	*Eucalyptus camaldulensis* Dehnh.	Myrtaceae	Thailand	CPC 25217	KT290170	KT290143	KT290197	Silva et al. (2016)
*Pseudocercospora tereticornis* Crous & Carnegie	*Eucalyptus tereticornis* Sm.	Myrtaceae	Australia	CBS 125214	MH874960	MH863460	–	Vu et al. (2019)
*Pseudocercospora vitis* (Lév.) Speg.	*Vitis vinifera* L.	Vitaceae	S. Korea	CPC 11595	–	–	JX901702	Quaedvlieg et al. (2012)
*Pseudocercosporella bakeri* (Syd. & P.Syd.) Deighton	*Ipomoea indica* (Burm.) Merr.	Convolvulaceae	New Zealand	CBS 119488	KX287005	KX287306	KX287862	Videira et al. (2016)
*Pseudocercosporella myopori* U.Braun & C.F.Hill	*Myoporum laetum* G.Forst.	Scrophulariaceae	New Zealand	CBS 114644	KX287000	KX287302	JX143491	Groenewald JZ et al. (2013)
*Zasmidium daviesiae* (Cooke & Massee) U.Braun, C. Nakash., Videira & Crous	*Daviesia latifolia* R.Br.	Fabaceae	Australia	CBS:116002	KF901928	KF901603	KF903373	Quaedvlieg et al. (2014)

**Table 2.** Data of sequences of cercosporoid fungi from Benin generated during the present study. Names of species proposed as new in this study are written in bold.

Species	Voucher	Host	Host family	GenBank Accession Numbers			
				nrSSU	nrLSU	ITS	tef1
***Cercospora beninensis***	YMM11	*Crotalaria macrocalyx* Benth.	Fabaceae	MW834445	MW834433	MW834437	MW848615
Cercospora aff. canescens Ellis & G.Martin	YMM07	*Calopogonium* sp.	Fabaceae	MW834475	–	MW834492	MW848605
	YMM01	*Vigna subterranea* (L.) Verdc.	Fabaceae	MW834473	MW834457	MW834490	MW848603
Cercospora cf. canscorina Chidd.	YMM05	*Vigna* sp.	Fabaceae	MW834474	MW834458	MW834491	MW848604
Cercospora cf. fagopyri K.Nakata & S.Takim.	YMM23A	*Lablab* sp.	Fabaceae	–	–	MW861543	MW848607
***Cercospora parakouensis***	YMM296A	*Desmodium tortuosum* (Sw.) DC.	Fabaceae	–	MW834436	MW834442	MW848621
*Cercospora phaseoli-lunati* U.Braun & Crous	YMM289	*Vigna radiata* (L.) R.Wilczek	Fabaceae	MW834471	–	MW834483	MW848601
***Cercospora rhynchophora***	YMM03B	*Vigna unguiculata* (L.) Walp.	Fabaceae	MW834447	MW834431	MW834443	MW848619
*Cercospora* sp.1	YMM3S	*Sorghum bicolor* (L.) Moench	Poaceae	MW834466	MW834452	MW834484	MW848600
*Cercospora* sp.2	YMM48S	*Sorghum bicolor* (L.) Moench	Poaceae	MW834467	MW834453	MW834485	MW848608
*Cercospora* sp.3	YMM229	*Spigelia* sp.	Loganiaceae	–	MW834462	MW834500	MW848599
*Cercospora* sp.4	YMM297B	*Phaseolus lunatus* L.	Fabaceae	MW834481	MW834464	MW834501	MW848612
***Cercospora tentaculifera***	YMM75	*Vigna unguiculata* (L.) Walp.	Fabaceae	MW834448	–	MW834440	MW848614
***Cercospora vignae*-*subterraneae***	YMM293	*Vigna subterranea* (L.) Verdc.	Fabaceae	MW834446	–	MW834438	MW848618
***Cercospora zorniicola***	YMM299	*Zornia glochidiata* DC.	Fabaceae	–	–	–	MW848616
*Nothopassalora personata* (Berk. & M.A.Curtis) U.Braun, C. Nakash., Videira & Crous	YMM49A	*Arachis hypogaea* L.	Fabaceae	MW834479	MW844038	MW834497	–
*Passalora arachidicola* (Hori) U.Braun	YMM49B	*Arachis hypogaea* L.	Fabaceae	MW845059	MW844039	MW834498	–
*Pseudocercospora bradburyae* E.Young	YMM275	*Centrosema pubescens* Benth.	Fabaceae	MW834465	–	–	MW848609
*Pseudocercospora cruenta* (Sacc.) Deighton	YMM288	*Phaseolus* sp.	Fabaceae	MW834472	MW834456	MW834489	MW848602
	YMM04	*Vigna unguiculata* (L.) Walp.	Fabaceae	MW834478	MW834461	MW834496	MW848606
	YMM03A	*Vigna unguiculata* (L.) Walp.	Fabaceae	MW834482	MW834460	MW834495	MW848613
	YMM294B	*Vigna unguiculata* (L.) Walp.	Fabaceae	MW834480	MW834463	MW834493	MW848611
	YMM125	*Vigna unguiculata* (L.) Walp.	Fabaceae	MW834476	MW834451	MW834499	MW848610
*Pseudocercospora griseola* (Sacc.) Crous & U.Braun	YMM297A	*Phaseolus lunatus* L.	Fabaceae	MW834477	MW834459	MW834494	–
***Pseudocercospora sennicola***	YMM12	*Senna occidentalis* (L.) Link	Fabaceae	MW834444	MW834432	MW850550	–
*Pseudocercospora* sp.3	YMM19	*Abelmoschus* sp.	Malvaceae	MW834470	–	MW834488	–
*Pseudocercospora* sp.1	YMM123	*Abelmoschus* sp.	Malvaceae	MW834468	MW834454	MW834486	–
***Pseudocercospora tabei***	YMM220	*Vigna unguiculata* (L.) Walp.	Fabaceae	MW834450	MW834434	MW834439	

## Data availability

The specimen data is available through the Dryad Digital Repository https://datadryad.org/ (https://doi.org/10.5061/dryad.73n5tb2x9).

## Results

### Phylogeny

We isolated DNA from a total of 28 specimens of cercosporoid fungi recently collected in Benin (Table 2). These specimens represent 18 species found on species of Fabaceae for which 76 sequences are provided: 20 sequences of 18S rDNA, 16 of 28S rDNA, 21 of ITS and 19 of *tef1*. The separately aligned data sets for each marker consisted of 35 sequences/893 base pairs for 18S rDNA, 60/719 for 28S rDNA, 82/437 for ITS and 74/160 for *tef1*.

**Figure 1.**
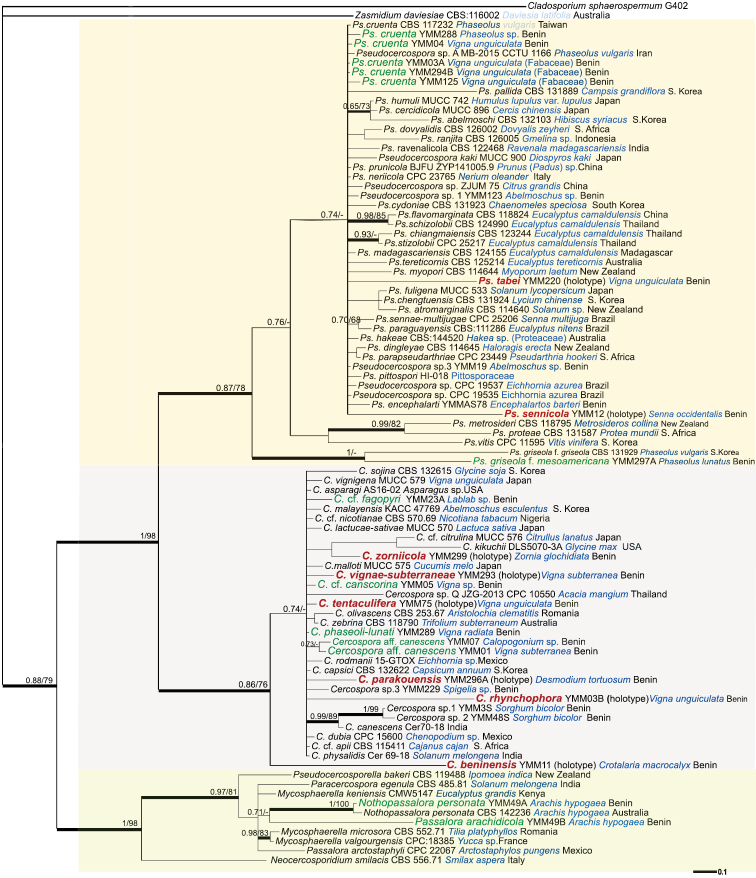
The Bayesian phylogenetic tree inferred from DNA sequence data from the multigene alignment (SSU rDNA, LSU rDNA, ITS and *tef1*) of cercosporoid species. Nodes receiving Bayesian PP ≥ 0.94 or MLBS ≥ 70% are considered as strongly supported and are indicated by thickened branches. Names of newly described species are written in bold and red. Species newly reported for Benin are indicated by green letters. Names of host plants are written with blue letters.

For the four-locus data analysis, DNA sequence data from the 18SrDNA, 28SrDNA, ITS and *tef1* gene regions were combined and submitted to Bayesian and Maximum Likelihood (ML) analyses. The final concatenated alignment contained a total of 91 specimens including the out-group (65 specimens from NCBI and 26 specimens from this study) and had an aligned length of 2212 characters including alignment gaps. As the ML analyses produced tree topologies mostly identical to results of Bayesian analyses, bootstrap support values of the ML trees were incorporated into the tree that resulted from Bayesian analyses (Fig. 1). In this tree, the cercosporoid fungi are grouped in three major clades: *Cercospora* (86/76), *Pseudocercospora* (87/78) and *Passalora* together with other species of other genera (100/98) (Fig. 1). Phylogenetic analyses of individual loci are deposited in TreeBASE. Details of results concerning the delimitation of species are mentioned and discussed as part of species notes below.

*Tef1* sequence data showed differences between closely related species in the genera *Cercospora* and *Pseudocercospora* and are more informative than ITS and LSU rDNA sequence data. Therefore, we provide molecular phylogenetic analyses based on new *tef1* sequences as well as sequences from GenBank for some newly described species, namely *Cercospora rhynchophora*, *C. parakouensis*, *C. zorniicola* and *Pseudocercospora tabei*. For *Ps. sennicola*, we provide an analysis based on ITS sequence data, because we were not able to obtain *Tef1* sequence data.

### Taxonomy

Based on morphological, molecular phylogenetic and host evidence, the cercosporoid fungi recently collected in Benin are assigned to 18 different taxa belonging to four genera. Among these, eight species are proposed as new to science, six in the genus *Cercospora* and two in *Pseudocercospora*. Eight species represent new reports for Benin, three of them are new for the whole of West Africa, namely Cercospora cf. canscorina, C. cf. fagopyri and *C. phaseoli-lunati*. Two species of cercosporoid fungi were previously reported in Benin and are confirmed.

#### Cercospora beninensis

Taxon classificationFungiMycosphaerellalesMycosphaerellaceae

Y.Meswaet, Mangelsdorff, Yorou & M.Piepenbr. sp. nov.

0EC4CB5E-A43D-56A5-B488-FC7786750295

839170

Figs 2A , 3

##### Etymology.

The epithet *beninensis* refers to the country of origin of the type specimens, Benin.

##### Diagnosis.

*Cercospora beninensis* differs from four *Cercospora* spp. known on *Crotalaria* spp. by having only internal hyphae, darker, shorter and narrower conidiophores [(14.5–)28.5–160(–168) × (3–)3.5–4.5(–5) μm] and mostly smaller and narrower conidia [(19–)23.5–122(–150) × (2.5–)3–4(–4.5) μm] (Table 3).

**Table 3.** Comparison of *Cercospora beninensis* (YMM11) on *Crotalaria macrocalyx* with *Cercospora* spp. known from *Crotalaria* spp. based on literature ^a–f^.

*Cercospora* species	Leaf spots colour, size	Stromata	Conidiophore size (in μm), branching, septa, colour	Conidium sizes (in μm), septa
*Cercospora beninensis* (YMM11)	Brown to reddish brown, 0.5(1.5–)–5.5 mm diam.	Small or lacking	(14.5–)28.5–160(–168) × (3–)3.5–4.5(–5), branched, 0–6(–8)-septate, dark brown	(19–)23.5–122(–150) × (2.5–)3.5–4(–4.5), 1–7(–9) septa
*C. apii* ^ab^	Present	Often small or lacking, occasionally developed, (up to 50 μm diam.)	20–300 × 4–6.5, rarely branched, multi-septate, pale brown, uniform in colour and width	25–315 × 3–6, (0–)3–25(–30) septa^b^
*C. canescens* ^a^	3–15 mm	Often small	20–200 × 3–6.5, rarely branched, multi-septate, pale to medium dark brown	25–300 × 2.5–5.5, indistinctly multi-septate
*C. demetrioniana* ^cde^	Rusty brown to dark brown, 1–1.5 mm.	Present	40–350 × 4–6 (–7) ^c^ or up to 1 mm^de^, 1–10-septate, unbranched, pale brown	50–210 × 3.5–5.5 /75–230 × 4–7, 7–16, very closely and indistinctly septate
C. demetrioniana f. minor ^f^	No information	No information	110–130 × 5–6	35–70(–170) × 5–5.5

^a^Hsieh and Goh (1990), ^b^Crous and Braun (2003), ^c^Chupp (1954), ^d^Saccardo (1886), ^e^Winter (1884), ^f^Ciferri and González-Fragosa (1926).

##### Type.

Benin. Borgou: Parakou, c. 363 m a.s.l., 9°20'29"N, 2°37'28"E, on *Crotalaria macrocalyx* Benth. (Fabaceae), 21 Sep 2019, Y. Meswaet and R. Dramani, YMM11 (***Holotype***: M-0312640; ***Isotype***: UNIPAR). ***Ex holotype sequences.***MW834445 (SSU), MW834433 (LSU), MW834437 (ITS), MW848615 (*tef1*).

##### Description.

***Leaf spots*** amphigenous, subcircular to angular-irregular, (0.5–)1.5–5.5 mm diam., brown to reddish brown, more evident on the adaxial surface of the leaves than on the abaxial side, occasionally with a chlorotic halo, the outermost ring darker than the inner ring, often with indefinite margin. ***Caespituli*** amphigenous, mainly epiphyllous, greyish brown to dark brown. ***Mycelium*** internal. Internal hyphae conspicuous, branched, 2.5–3.5 μm wide, septate, pale brown. ***Stromata*** lacking or formed by few aggregated swollen hyphal cells. ***Conidiophores*** in small, loose to moderately dense fascicles of up to approx. 16 conidiophores, occasionally solitary, arising from internal hyphae breaking through the adaxial epidermis of the leaves or penetrating through stomatal openings, erect, straight, subcylindrical, 1–2(–3) times geniculate, sometimes attenuated towards the tips, occasionally branched, (14.5–)28.5–160(–168) × (3–)3.5–4.5(–5) μm, 0–6(–8)-septate, brown to dark brown. ***Conidiogenous cells*** monoblastic or proliferating sympodially, sometimes distinctly subdenticulate; loci 1.5–2.5(–3.5) μm wide, thickened and darkened. ***Conidia*** solitary, acicular to narrowly obclavate, straight to curved, (19–)23.5–122(–150) × (2.5–)3–4(–4.5) μm, 1–7(–9)-septate, hyaline, smooth, tip acute, base truncate to short obconically truncate, 2.5–3(–4) µm wide, hila thickened and darkened.

##### Additional specimens examined.

Benin. Borgou: Parakou, on the way to Okpara forest, c. 323 m a.s.l., 9°18'11"N, 2°43'50"E, on *Crotalaria macrocalyx*, 3 Sep 2019, Y. Meswaet and R. Dramani, YMM274 (***Paratypes***: M-0312641; UNIPAR). Benin. Borgou: N’Dali, c. 380 m a.s.l., 9°52'33"N, 2°41'20"E, same host, 31 Aug 2019, Y. Meswaet and A. Tabé, YMM272 (M-0312642).

##### Host and distribution.

On *Crotalaria macrocalyx* (Fabaceae) in Benin.

##### Notes.

Currently, three species and one form of *Cercospora* are known on *Crotalaria* spp., namely *C. apii*, *C. canescens*, *C. demetrioniana* G.Winter and C. demetrioniana f. minor Gonz. Frag. & Cif. (Farr and Rossman 2021). *C. beninensis* is morphologically distinct from all of them (Table. 3). *C. apii* differs by conidiophores that are more abundant on the abaxial surface of the leaves, in large and dense fascicles and longer [20–300 µm versus (14.5–)28.5–160(–168) in *C. beninensis*] as well as by longer and wider conidia [(25–315 × 3–6 µm versus (19–)23.5–122(–150) × (2.5–)3–4(–4.5) in *C. beninensis*] with more numerous septa (Chupp 1954; Crous and Braun 2003). *C. canescens* causes larger leaf spots often along the leaf margin, paler conidiophores that are more abundant on the abaxial leaf surface and longer conidia [(30–300) µm versus (19–)23.5–122(–150) μm in *C. beninensis*] (Chupp 1954). The distinctness is confirmed by molecular data. *C. demetrioniana* produces unbranched, paler, longer and wider conidiophores [40–350 × 4–6(–7) µm, in the original description a length of up to 1 mm is mentioned, versus (14.5–)28.5–160(–168) × (3–)3.5–4.5(–5) in *C. beninensis*] and above all, longer and wider conidia (75–230 × 4–7 µm with 7–16 indistinct septa versus (19–)23.5–122(–150) × (2.5–)3–4(–4.5) μm with 1–7(–9) distinct septa in *C. beninensis*] (Winter 1884; Saccardo 1886; Chupp 1954). C. demetrioniana f. minor differs from the present species by shorter and wider conidiophores (110–130 × 5–6 µm) and wider conidia (5–5.5 µm) (Ciferri and González-Fragoso 1926).

*C. beninensis* is distinct from all known species for which DNA sequence data are available based on its position in the multi-gene (Fig. 1) and in the *tef1* phylogeny (see Suppl. material 4). In the ITS phylogeny, *C. beninensis* cannot be distinguished from other *Cercospora* spp. (see Suppl. material 3).

#### Cercospora aff. canescens

Taxon classificationFungiMycosphaerellalesMycosphaerellaceae

Ellis & G.Martin, Am. Nat. 16(12): 1003 (1882).

C45944C4-8F5B-57C2-A039-D89D88EBFE36

179841

Figs 2B, C , 4

##### Type.

USA (no further data available), on *Phaseolus* sp. (Fabaceae), 1882, s.n. (“Type?” NY, n.v.).

For synonyms see Crous and Braun (2003) or MycoBank.

##### Description.

***Leaf spots*** amphigenous, subcircular to irregularly angular, 3–11.5(–13) mm diam., occasionally crossing veins, reddish brown to slightly dark brown, with dark margin. ***Caespituli*** amphigenous, greyish brown to dark brown. ***Mycelium*** internal and external. Internal hyphae often indistinct. External hyphae branched, 2.5–3.5 μm wide, septate, olivaceous brown to brown, smooth. **Stromata** lacking or formed by few aggregated swollen hyphal cells, immersed in the mesophyll or in substomatal cavities, dark brown. ***Conidiophores*** in small, loose fascicles of up to 8, arising from stromata, breaking through the adaxial epidermis of the leaves or penetrating through stomatal openings, sometimes solitary arising through stomatal openings or erumpent through the cuticle, erect, straight to sinuous or somewhat geniculate, rarely branched, (16.5–)21–152(–165) × (4–)4.5–5.5 μm, 1–6-septate, brown to dark brown. ***Conidiogenous cells*** terminal, monoblastic to polyblastic, brown; loci 1.5–2.5 (–3) μm wide, thickened and darkened. ***Conidia*** solitary, narrowly obclavate to subacicular, straight to curved, (34–)38–280(–330) × (3–)3.5–4(–4.5) μm, 3–12(–14)-septate, hyaline to subhyaline, smooth, apex subacute or acute, base truncate to short obconically truncate, up to 2.5 μm wide, hila thickened and darkened.

##### Specimens examined.

Benin. Borgou: Parakou, c. 363 m a.s.l., 9°20'29"N, 2°37'28"E, on *Calopogonium* sp., 21 Sep 2019, Y. Meswaet and A. Tabé, YMM07 (M-0312643, UNIPAR). Benin. Borgou: Parakou, c. 395 m a.s.l., 9°21'27"N, 2°36'44"E, *Calopogonium* sp., 17 Sep 2019, Y. Meswaet and A. Tabé, YMM08 (M-0312644). Benin. Borgou: Parakou, c. 395 m a.s.l., 9°21'27"N, 2°36'44"E, on *Vigna subterranea*, 16 Sep 2019, Y. Meswaet and R. Dramani, YMM01 (M-0312645, UNIPAR).

##### Herbarium specimens examined for comparison.

*C. canescens*. On *Vigna unguiculata* (as *V. sinensis* L.): El Salvador. Sacocoyo, 3 Jul 1943, Wellman F. L. 140 (BPI 434127B). On *V. unguiculata* (as *V. sinensis*): USA. Illinois: Gallatin County, 8 Sep 1932, G.H. Boewe B331 (ILL23703 ***Holotype*** of *C. vignicaulis* Tehon). On *V. unguiculata*: USA. Illinois: Pulaski, Olmstead, 17 Sep 1933, G.H. Boewe s.n. (ILL24809 ***Paratype*** of *C. vignicaulis*). On *V. unguiculata* (as *V. sinensis*): USA. Illinois: White, Carmi., 10 Sep 1934, G.H. Boewe B588 (ILL 25450 ***Paratype*** of *C. vignicaulis*).

##### Hosts and distribution.

On many species of Fabaceae and of other families (Crous and Braun 2003), known worldwide, from Australia, Bangladesh, Brazil, Bolivia, Brunei, Cambodia, China, Colombia, Costa Rica, Cuba, Dominican Republic, Ecuador, Fiji, Ghana, Guyana, Haiti, Hong Kong, India, Indonesia, Iran, Japan, Kenya, Korea, Malawi, Malaysia, Malawi, Mauritius, Myanmar, Nepal, New Caledonia, New Zealand, Nigeria, Pakistan, Panama, Papua New Guinea, Peru, Philippines, Puerto Rico, Russia, Senegal, Sierra Leone, Solomon Islands, Somalia, South Africa, Saint Vincent and the Grenadines, Sudan, Tadzhikistan, Taiwan, Tanzania, Thailand, Trinidad and Tobago, Togo, Uganda, USA, Uzbekistan, Vanuatu, Venezuela, Zambia, Zimbabwe (Chupp 1954; Ellis 1976; Shin and Kim 2001; Crous and Braun 2003; Farr and Rossman 2021).

##### Notes.

**Figure 2.**
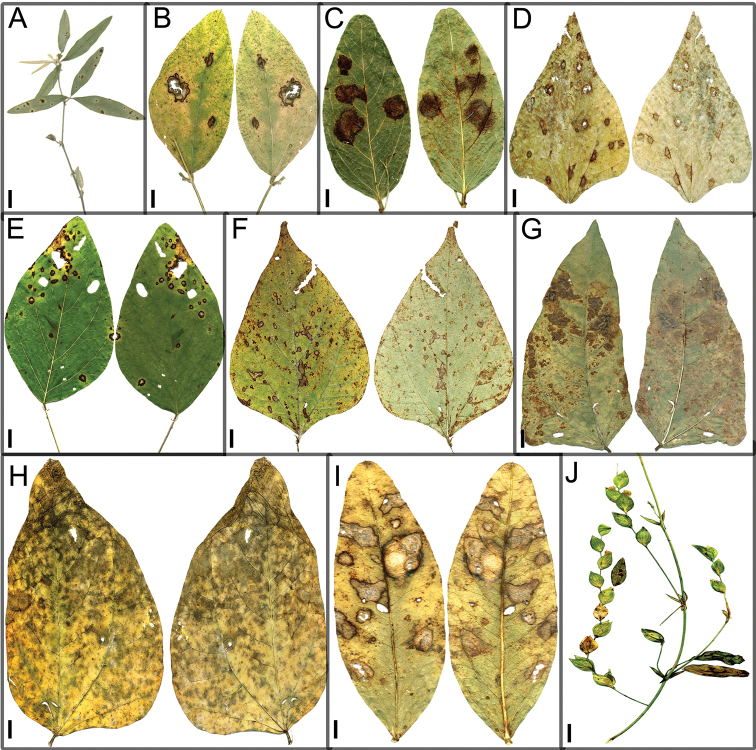
Leaf spot symptoms associated with *Cercospora* spp. **A***Cercospora beninensis* on *Crotalaria macrocalyx* (YMM11) **B**Cercospora aff. canescens on *Calopogonium* sp. (YMM07) **C**Cercospora aff. canescens on *Vigna subterranea* (YMM01) **D***Cercospora fagopyri* on *Lablab* sp. (YMM23A) **E***Cercospora parakouensis* on *Desmodium tortuosum* (YMM296A) **F***Cercospora phaseoli-lunati* on *Vigna radiata* (YMM289) **G***Cercospora rhynchophora* on *Vigna unguiculata* (YMM03B) **H***Cercospora tentaculifera* on *Vigna unguiculata* (YMM75) **I***Cercospora vignae-subterraneae* on *Vigna subterranea* (YMM293) **J***Cercospora zorniicola* on *Zornia glochidiata* (YMM299). Scale bars: 10 mm (**A, C, F, G**); 12 mm (**B, D, E, H, J**); 6 mm (**I**).

The present *Cercospora* sp. on *Calopogonium* sp. also occurs on *Vigna subterranea* with different leaf spot appearances and caespituli. The lesions on *Calopogonium* sp. appear to be associated with a species of Pleosporales, whereas the leaf lesions on *V. subterranea* apparently are not associated with any other fungus and are dark reddish brown to dark brown with a dark margin, which are typical symptoms caused by *Cercospora* spp. The lesions on *V. subterranea* are larger and more abundant than those on *Calopogonium* sp., with abundant, dense caespituli and with dark greyish brown pigmentation (Fig. 2C).

**Figure 3.**
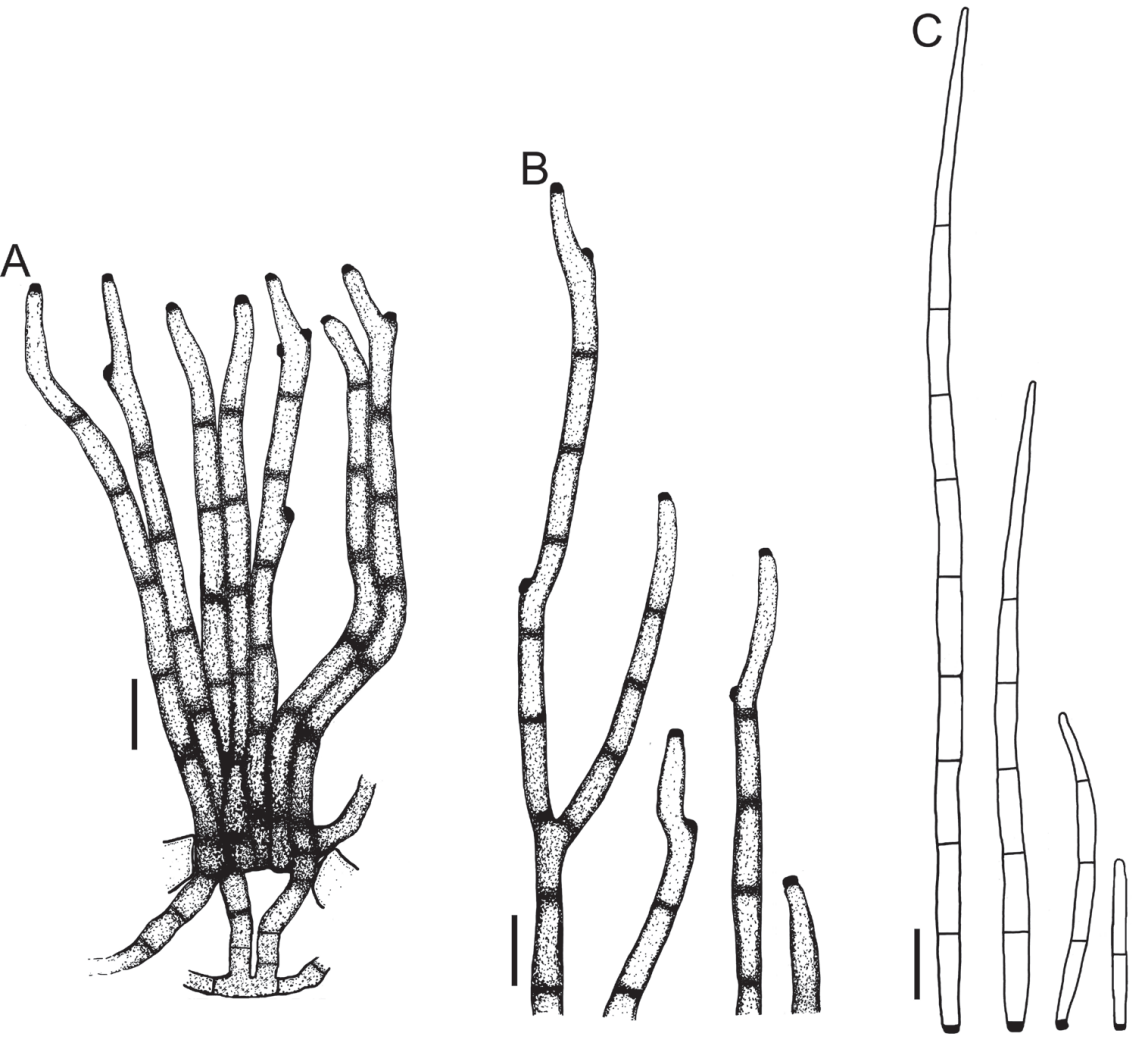
*Cercospora beninensis* on *Crotalaria macrocalyx* (YMM11) **A** fascicle of conidiophores **B** individual conidiophores **C** conidia. Scale bars: 15 μm (**A**); 10 μm (**B, C**).

*Cercospora canescens* is the only species of *Cercospora* known for *Calopogonium* spp. (Farr and Rossman 2021) and has been reported from West Africa (Guinea) on *Calopogonium mucunoides* (Lenné 1990). Apart from having slightly narrower conidia [(3–)3.5–4(–4.5) μm versus 2.5–5.5(–6) μm in *C. canescens*] as described by Chupp (1954), Hsieh and Goh (1990) and Mulder and Holliday (1975), the present specimen from Benin is morphologically identical to *C. canescens*. In the phylogenetic analyses, however, DNA sequences of the two specimens from Benin cluster together but separately from sequences of *C. canescens* available from India. In the multi-gene tree (Fig. 1), *C. canescens* is located on a branch in a clade together with sequences of *Cercospora* spp. YMM3SO and YMM48SO on *Sorghum bicolor* (Poaceae) from Benin. *C. canescens* is known to correspond to a species complex that shows diverse morphological characteristics and genetic diversity (Joshi et al. 2006; Groenewald et al. 2013). Although *C. canescens* is an economically important species, no sequence data from the type or a neotype specimen are available (e.g., Groenewald et al. 2013). These are indispensable to resolve the *C. canescens* species complex. The specimens collected in Benin are tentatively placed into the species complex of *C. canescens* until DNA sequence data from the type locality (USA) and from diverse host species are available. C. aff. canescens is cited here for the first time for Benin (Piepenbring et al. 2020).

**Figure 4.**
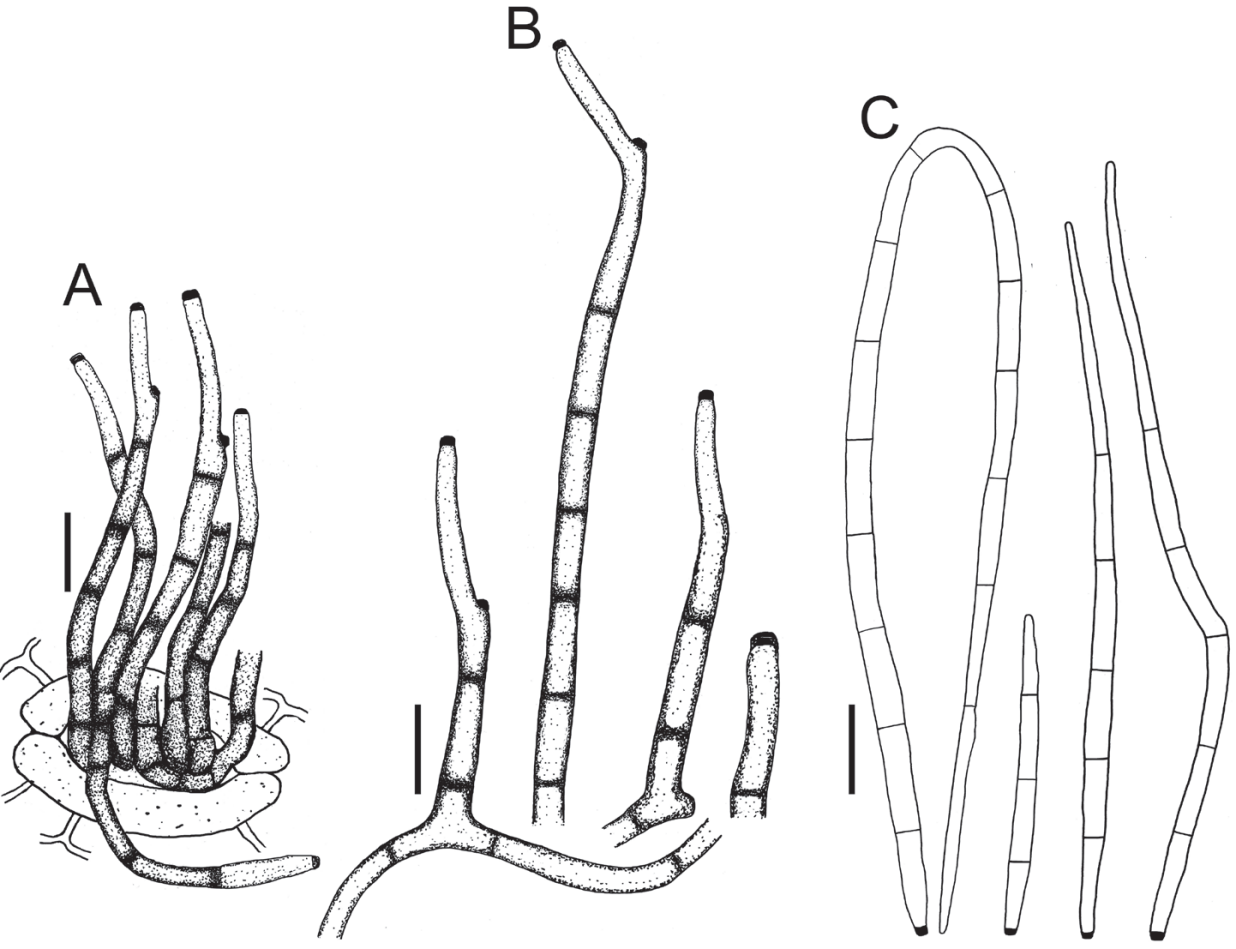
Cercospora aff. canescens on *Calopogonium* sp. (YMM07) **A** fascicle of conidiophores protruding from a stomatal opening **B** solitary conidiophores **C** conidia. Scale bars: 15 μm (**A, C**); 10 μm (**B**).

#### Cercospora cf. canscorina

Taxon classificationFungiMycosphaerellalesMycosphaerellaceae

Chidd., Sydowia 13 (1–6): 155. 1959.

CB5EDB98-0F7C-5816-9CB8-264ABD41172D

294326

Fig. 5

##### Type.

India. R. Br. Khandala (Maharashtra), on *Canscora diffusa* (Vahl) R.Br. ex Roem. & Schult. (Gentianaceae), 9 Nov 1956, Chiddarwar 4 (***Holotype***: IMI 83165, n.v.; ***Isotype*s**: HCIO, BPI, n.v.).

##### Description.

***Leaf spots*** amphigenous, subcircular to irregularly angular, 2.5–8 mm diam., brown to reddish brown, with a dark margin. ***Caespituli*** amphigenous, greyish brown to brown. ***Mycelium*** internal. **Stromata** lacking or formed by few substomatal aggregated swollen hyphal cells. ***Conidiophores*** in small, loose fascicles to moderately large and dense fascicles of up to approx. 22 conidiophores, arising from internal hyphae breaking through the adaxial epidermis of the leaves or penetrating through stomatal openings, sometimes solitary, erect, straight, subcylindrical, 1–2 times geniculate, unbranched, (12–)20.5–68(–72) × (3–)3.5–4.5 μm, 0–6-septate, brown to dark brown. ***Conidiogenous cells*** terminal, usually monoblastic, sometimes polyblastic; loci apical or sometimes located on the shoulders of geniculations, 1.5–2.5(–3) μm wide, thickened and darkened. ***Conidia*** solitary, acicular to narrowly obclavate, straight to curved, 22–76(–80) × 2.5–3.5 μm, 1–7-septate, hyaline, smooth, tip acute, base truncate to short obconically truncate, 2–3 µm wide, hila thickened and darkened.

##### Specimens examined.

Benin. Borgou: Parakou, c. 386 m a.s.l., 9°20'35"N, 2°36'37"E, on *Vigna* sp., 14 Sep 2019, Y. Meswaet and R. Dramani, YMM05 (M-0312646; UNIPAR).

##### Hosts and distribution.

On *Canscora diffusa* (Gentianaceae) from Khandala, West India (Chiddarwar 1959) and on *Vigna unguiculata* (as *Vigna catjang* (Burm.f.) Walp.) from India (Bhat and Pratibha 2010). C. cf. canscorina is reported here for the first time for Benin and for Africa.

##### Notes.

Seven species of *Cercospora* have previously been recorded on *Vigna* spp., namely *C. apii*, *C. canescens*, *C. canscorina*, *C. caracallae* (Speg.) Vassiljevsky & Karak., *C. kikuchii*, *C. longispora* Peck and *C. vignigena* C. Nakash., Crous, U.Braun & H.D. Shin (Farr and Rossman 2021). The present species from Benin is morphologically identical to *C. canscorina* (Chiddarwar 1959; Bhat and Pratibha 2010) except for narrower conidiophores with (3–)3.5–4.5 μm versus 3–7 μm in *C. canscorina* as mentioned by Bhat and Pratibha (2010). The original specimen of *C. canscorina* was not available for morphological examination and no DNA sequence data are currently published for this species. Therefore, a reliable species identification is not possible. The application of the name for the collections from Benin is tentative and must be verified based on sequences derived from the Indian type specimen or similar samples. C. cf. canscorina differs from all species of *Cercospora* on other members of Fabaceae from Benin by producing unbranched, relatively pale conidiophores and above all, shorter conidiophores [(12–)20.5–68(–72) μm] and conidia [22–76(–80) μm]. Based on the multi-gene tree (Fig. 1) it is not possible to distinguish Cercospora cf. canscorina from many other *Cercospora* spp.

**Figure 5.**
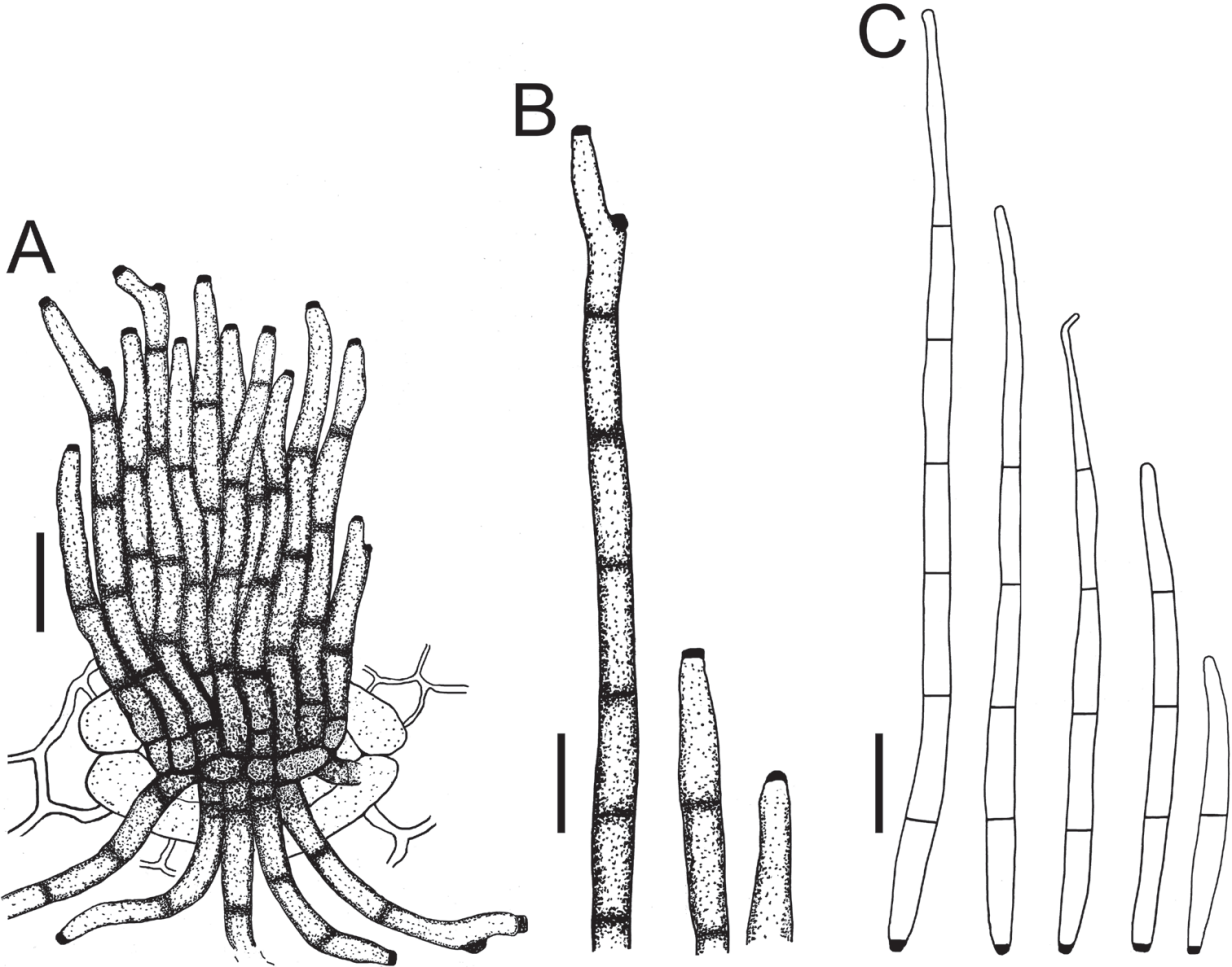
Cercospora cf. canscorina on *Vigna* sp. (YMM05) **A** fascicle of conidiophores protruding from a stomatal opening **B** solitary conidiophores **C** conidia. Scale bars: 15 μm (**A**); 10 μm (**B, C**).

#### Cercospora cf. fagopyri

Taxon classificationFungiMycosphaerellalesMycosphaerellaceae

K.Nakata & S.Takim., J. Agric. Exp. Stat. Gov. Gen. Chosen 15: 29. 1928.

A814C1D6-FEDF-525E-B0AE-A21ACDBB5A21

456931

Figs 2D , 6

##### Type.

South Korea. Suwon, on *Fagopyrum esculentum* Moench (Polygonaceae), Sep 1934, K. Nakata & S. Takimoto (holotype specimen, not located and not preserved according to Groenewald et al. (2013), ***neotype***: CBS H-21008, n.v).

For synonyms see Groenewald et al. (2013) or MycoBank.

##### Description.

***Leaf spots*** amphigenous, circular to subcircular or rarely irregularly angular, 2–5 mm diam., more or less limited by veins, reddish to pale brown, margin dark brown on the adaxial surface, less conspicuous on the abaxial surface. ***Caespituli*** amphigenous, conspicuous, greyish brown to dark brown. ***Mycelium*** internal and external. External hyphae branched, often inconspicuous, 1.5–3 μm wide, septate, olivaceous brown to brown, smooth. ***Stromata*** lacking to well-developed, 10–45 µm diam., dark brown, substomatal or breaking through the epidermis. ***Conidiophores*** in small, loose to moderately dense fascicles of up to approx. 14 conidiophores, arising from stromata breaking through the adaxial epidermis of the leaves or through stomatal openings, sometimes solitary arising from external hyphae, erect, straight, subcylindrical to geniculate, unbranched, (22.5–)36–157(–168) × 3–4(–5) μm, 2–6(–8)-septate, brown to dark brown. ***Conidiogenous cells*** terminal, with 1–2 loci; loci mainly apical, sometimes located on the shoulders of geniculations, 1.5–2(–3) μm wide, thickened and darkened. ***Conidia*** solitary, acicular to narrowly obclavate, straight to somewhat curved, (24–)27.5–70(–78) × (2–)2.5–3(–4) μm, with 2–5(–6) somewhat indistinct septa, hyaline, smooth, tip acute, base truncate to short obconically truncate, 1.5–2.5 μm wide, hila thickened and darkened.

##### Specimens examined.

Benin. Donga: Taneka-Koko, c. 441 m a.s.l., 9°51'30"N, 1°29'34"E, on *Lablab* sp., 29 Jul 2017, Y. Meswaet, M. Piepenbring, N. S. Yorou and participants of the summer school 2017, YMM23A ((M-0312647; UNIPAR). Same locality and host, 03 Aug 2016, Y. Meswaet, M. Piepenbring, N. S. Yorou and participants of the summer school 2016, YMM02 (M-0312648).

##### Hosts and distribution.

On *Cercis chinensis* (Fabaceae), *Cosmos bipinnata* Cav. (Asteraceae), *Fallopia dumetorum* (L.) Holub and *Fagopyrum esculentum* (Polygonaceae), *Hibiscus syriacus* (Malvaceae), *Viola mandshurica* W. Becker (Violaceae), from China, Japan, South Korea, Taiwan, Uganda and Venezuela (Hsieh and Goh 1990; Groenewald et al. 2013). C. cf. fagopyri is cited here for the first time on *Lablab* sp. and the first time for Benin and West Africa.

##### Notes.

Currently there are two species of the genus *Cercospora* known on hosts belonging to *Lablab*, namely *C. canescens* and *C. apii*. The present *Cercospora* sp. (YMM23A) differs from *C. canescens* in leaf spot size, stromata and septation characteristics, as well as unbranched conidiophores. Above all, the sizes of the conidia of the present species are different [(24–)27.5–70(–78) × (2–)2.5–3(–4) μm versus 30–300 × 2.5–5 (–6) µm in *C. canescens*]. *C. apii* differs by often small or lacking stromata, dense fascicules of up to 30 conidiophores, branched, longer conidiophores [20–300 μm versus (22.5–)36–157(–168) μm in C. cf. fagopyri] and above all, longer and wider conidia [25–315 × 3–6 μm versus (24–)27.5–70(–78) × (2–)2.5–3(–4) μm in C. cf. fagopyri] (Chupp 1954).

Our sequence of the *tef1* region of the specimen YMM23A from Benin is 100% similar to a sequence of *Cercospora fagopyri* on *Fallopia dumetorum* (GenBank JX143353) (Identities 233/233, i.e., 100%) and 99% similar to a further sequence of *C. fagopyri* on *Fagopyrum esculentum* (GenBank JX143352; Identities; 233/234, i.e., 99%). The identification of the present specimen as C. cf. fagopyri is only based on molecular data. Morphologically, descriptions of specimens of *C. fagopyri* on diverse host species in the literature differ and are quite confusing (Hsieh and Goh 1990; Groenewald et al. 2013). In order to establish a morphological concept and to know the host range of *C. fagopyri*, fresh specimens need to be collected once again on *Fagopyrum esculentum* in Korea, where this species was originally collected and pathogenicity needs to be proven for diverse host species.

**Figure 6.**
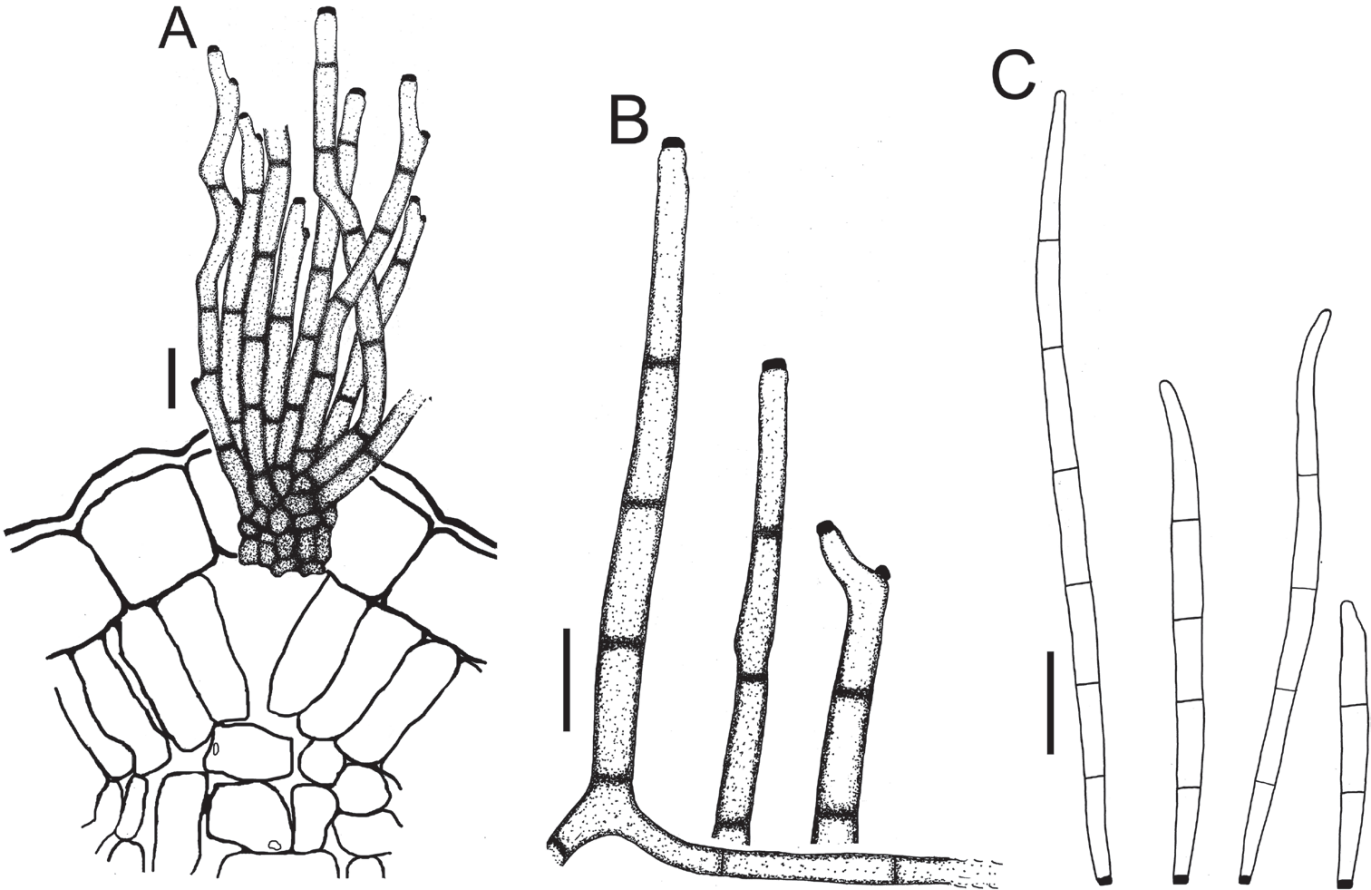
Cercospora cf. fagopyri on *Lablab* sp. (YMM23A) **A** fascicle of conidiophores growing out from a slightly developed stroma in the epidermis shown as part of a transverse section of a leaf **B** solitary conidiophores **C** conidia. Scale bars: 15 μm (**A**); 10 μm (**B, C**).

#### Cercospora parakouensis

Taxon classificationFungiMycosphaerellalesMycosphaerellaceae

Y.Meswaet, Mangelsdorff, Yorou & M.Piepenbr. sp. nov.

51755D67-071A-52FE-A3BA-6E9489B5660B

839171

Figs 2E , 7

##### Type.

Benin. Borgou: Parakou, Tankaro, c. 360 m a.s.l., 9°23'01"N, 2°30'36"E, on *Desmodium tortuosum* (Sw.) DC. (Fabaceae), 20 Sep 2019, Y. Meswaet and R. Dramani, YMM296A (***Holotype***: M-0312649; ***Isotype***: UNIPAR). ***Ex holotype sequences.***MW834436 (LSU), MW834442 (ITS), MW848621 (*tef1*).

##### Etymology.

The epithet *parakouensis* refers to the city of the type collection, Parakou, Benin.

##### Diagnosis.

*Cercospora parakouensis* differs from the two *Cercospora* species known on *Desmodium* spp., namely *C. canescens* and *C. kashiensis* Bharadwaj by producing almost no stromata, branched, darker and shorter conidiophores [(12.5–)18–178(–190) μm] and non- pigmented and shorter conidia [(14–)19–88(–113.5) × 3.5–4.5(–5) μm].

##### Description.

***Leaf spots*** almost lacking to well-developed, amphigenous, subcircular to irregularly angular, 1.5–5 mm diam., darkish brown to reddish brown, often with a diffuse whitish centre surrounded by a darker margin. ***Caespituli*** amphigenous, greyish brown to dark brown. ***Mycelium*** mainly internal. **Stromata** lacking. ***Conidiophores*** in small, loose fascicles, sometimes arising from internal hyphae, breaking through the adaxial epidermis of the leaves or penetrating through stomatal openings, occasionally solitary, arising through stomatal openings, erect, straight to sinuous or somewhat geniculate, occasionally branched, (12.5–)18–178(–190) × (3.5–)4–5(–5.5) μm, 1–6(–8)-septate, brown to dark brown. ***Conidiogenous cells*** terminal or rarely intercalary, usually monoblastic, rarely polyblastic; loci subcircular, 1.5–3 μm wide, thickened and darkened, refractive. ***Conidia*** solitary, narrowly obclavate to subacicular, straight to curved, (14–)19–88(–113.5) × 3.5–4.5(–5) μm, 2–6-septate, hyaline, smooth, apex subacute or acute, base truncate to short obconically truncate, 2–3(–3.5) μm wide, hila thickened and darkened.

##### Additional specimens examined.

Benin. Borgou: Parakou, c. 395 m a.s.l., 9°21'27"N, 2°36'44"E, on *Desmodium tortuosum*, 17 Sep 2019, Y. Meswaet and A. Tabé, YMM292 (***Paratypes***: M-0312650; UNIPAR).

##### Herbarium specimens examined for comparison.

See Cercospora aff. canescens.

##### Host and distribution.

On *Desmodium tortuosum* (Fabaceae) from Benin.

##### Notes.

Currently, two *Cercospora* species are known from *Desmodium* spp., namely *C. canescens* and *C. kashiensis* (Farr and Rossman 2021). *C. canescens* differs from the present species by causing large leaf spots often along the margin of the leaf, 3–15 mm in extent, paler conidiophores and above all, longer conidia [30–300 µm versus (14–)19–88(–113.5) µm in *C. parakouensis*] (Chupp 1954). The distinctness is also confirmed by molecular data (Fig. 1). *C. kashiensis* described on *Desmodium gangeticum* (L.) DC. from India causes different leaf spots, has unbranched and longer conidiophores (40–282 µm versus (12.5–)18–178(–190) in *C. parakouensis*) and above all, pigmented and longer conidia (16–220 µm versus (14–)19–88(–113.5) µm in *C. parakouensis*) with 2–15 septa (Bharadwaj 1971).

In the multi-gene tree (Fig. 1), the ITS and the *tef1* phylogeny (see Suppl. materials 3, 4), *C. parakouensis* forms part of a polytomy with a relatively large genetic distance (branch length) in relation to other sequences considered in the analysis.

Based on a MegaBLAST search using the *tef1* sequence, the closest matches in NCBI’s GenBank nucleotide database were *Cercospora nicotianae* on *Nicotiana tabacum* (Solanaceae) from China (GenBank MK881748; Identities 283/291, i.e., 97%), Cercospora cf. sigesbeckiae on *Persicaria orientalis* L. (Polygonaceae) from South Korea (GenBank JX143412; Identities 283/291, i.e., 97%) and Cercospora aff. canescens on a species of Malvaceae from Mexico (GenBank JX143321; Identities 283/291, i.e., 97%).

**Figure 7.**
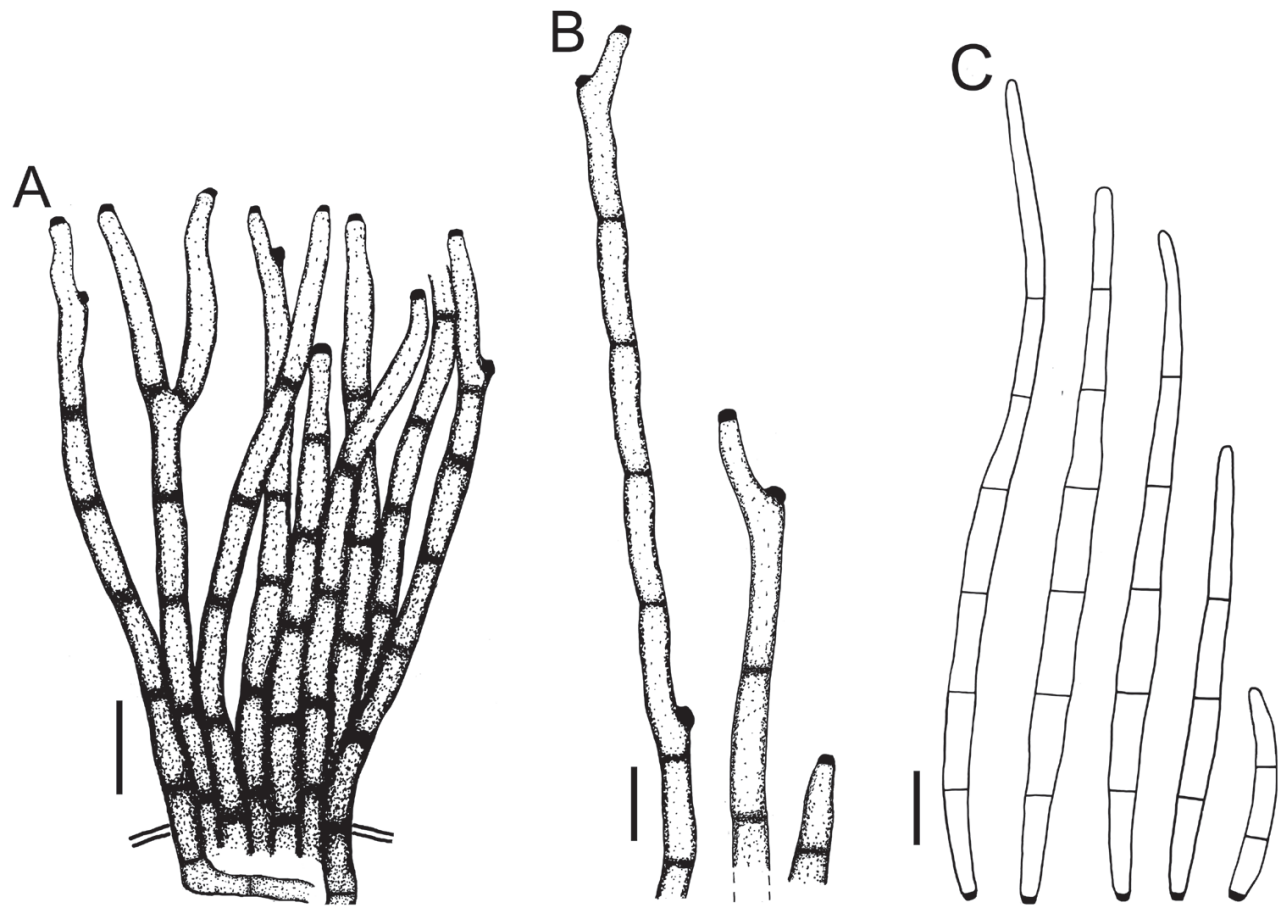
*Cercospora parakouensis* on *Desmodium tortuosum* (YMM296A) **A** fascicle of erumpent conidiophores **B** solitary conidiophores **C** conidia. Scale bars: 15 μm (**A**); 10 μm (**B, C**).

#### Cercospora phaseoli-lunati

Taxon classificationFungiMycosphaerellalesMycosphaerellaceae

U.Braun & Crous, Mycotaxon 92: 396. 2005.

25643304-DB25-5DB4-8E42-0E6E3FDDD0DB

**Figure 8.**
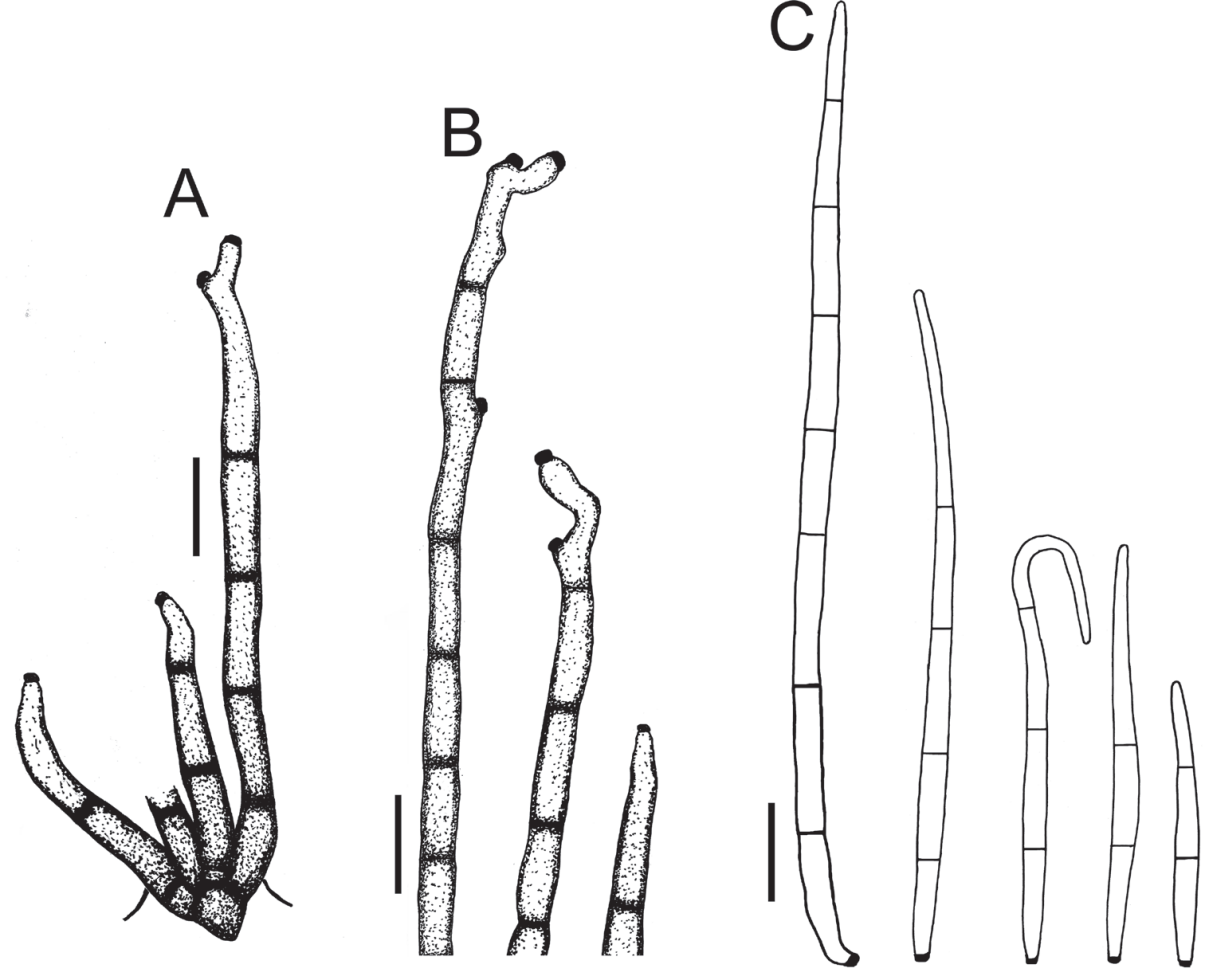
*Cercospora phaseoli-lunati* on *Vigna radiata* (YMM289) **A** small fascicle of conidiophores **B** solitary conidiophores **C** conidia. Scale bars: 12 μm (**A**); 10 μm (**B, C**).

MycoBank No: 500171 Figs 2F , 8

##### Type.

USA. Alabama: Tuskegee, on *Phaseolus lunatus* (Fabaceae), 5 Jul 1897, G.W. Carver 290 (***Holotype***NY, n.v.).

##### Description.

***Leaf spots*** amphigenous, subcircular to irregularly angular, 2.5–8(–12) mm diam., more or less limited by veins, whitish grey to greyish brown, with a narrow to wide dark brown margin on the adaxial surface, less conspicuous on the abaxial surface. ***Caespituli*** amphigenous, mainly epiphyllous, scattered, brown to dark brown. ***Mycelium*** internal, indistinct. External hyphae absent. ***Stromata*** lacking or formed by few aggregated swollen hyphal cells, immersed in the mesophyll or in substomatal cavities. ***Conidiophores*** in small, loose fascicles of up to 6, arising from internal hyphae of small hyphal aggregations, breaking through the adaxial epidermis of the leaves or penetrating through stomatal openings, or solitarily arising through stomatal openings, erect, rarely branched, straight to geniculate or subcylindrical to mostly attenuated towards the tips, conical or irregularly shaped, (18–)21.5–94(–102) × (3.5–)4–5 μm, 1–6-septate, smooth, brown to dark brown. ***Conidiogenous cells*** terminal, monoblastic or polyblastic; loci distinct, up to 2.5 μm wide, thickened and darkened. ***Conidia*** solitary, narrowly obclavate to subacicular, straight to curved, (16–)19–94(–105.5) × 2.5–3.5 μm, 2–7-septate, hyaline to subhyaline, smooth, apex subacute or acute, base truncate to short obconically truncate, up 2.5 μm wide, hila thickened and darkened.

##### Specimen examined.

Benin. Borgou: Parakou, c. 386 m a.s.l., 9°20'35"N, 2°36'37"E, on *Vigna radiata*, 14 Sep 2019, Y. Meswaet and R. Dramani, YMM289 (M-0312651 UNIPAR).

##### Hosts and distribution.

On *Phaseolus lunatus* from USA, Alabama, Tuskegee (type locality) (Braun and Crous 2005). This species is cited here for the first time for Benin. Thereby, it is cited for the first time for West Africa. *Vigna radiata* is a new host species.

##### Notes.

Thirteen *Cercospora* species have previously been recorded on species of *Vigna* and *Phaseolus*, namely *C. albida* Matta & Belliard, *C. apii*, *C. canescens*, *C. canscorina*, *C. caracallae*, *C. kikuchii*, *C. longispora*, *C. olivascens*, *C. phaseoli-lunati*, *C. phaseolicola* U.Braun & Mouch., *C. phaseolina* Speg., *C. vignigena* and *C. zonata* G. Winter (Farr and Rossman 2021). Among these, *C. caracallae* and *C. phaseoli-lunati* are morphologically rather similar to the present collection. *C. caracallae*, however, differs in causing distinct leaf spots and caespituli, dense fascicules composed of unbranched and wider conidiophores [5–6 μm versus (3.5–)4–5 μm in *C. phaseoli-lunati*] and wider conidia (3–5.5 μm versus 2.5–3.5 μm in *C. phaseoli-lunati*) with less septa (3–5 versus 2–7 septa) (Chupp 1954; Spegazzini 1910). Except for the presence of distinct leaf spots and sporulation, the morphology of the present collection from Benin fits well to the original description of *C. phaseoli-lunati* on *Phaseolus lunatus* from the USA provided by Braun and Crous (2005). Based on the present phylogenies, it is not possible to distinguish *C. phaseoli-lunati* from numerous other *Cercospora* spp.

#### Cercospora rhynchophora

Taxon classificationFungiMycosphaerellalesMycosphaerellaceae

Y.Meswaet, Mangelsdorff, Yorou & M.Piepenbr. sp. nov.

B0B89FDF-0245-5ACA-B79E-D26E25E18386

839172

Figs 2G , 9

##### Type.

Benin. Borgou: Parakou, c. 385 m a.s.l., 9°20'34"N, 2°36'39"E, on *Vigna unguiculata* (L.) Walp. (Fabaceae), 14 Sep 2019, Y. Meswaet and R. Dramani, YMM03B (***Holotype***: M-0312652; ***Isotype***: UNIPAR). ***Ex holotype sequences*.**MW834447 (SSU), MW834431 (LSU), MW834443 (ITS), MW848619 (*tef1*).

##### Etymology.

The epithet *rhynchophora* refers to the beak- or hook-like tips of the conidiophores, a characteristic of this species.

##### Diagnosis.

*Cercospora rhynchophora* differs from other *Cercospora* spp. known on *Vigna* spp. by causing distinct leaf spots, often well-developed stromata and up to 4 times geniculate conidiophores with often polyblastic conidiogenous cells with irregular, often beak-shaped tips.

##### Description.

***Leaf spots*** amphigenous, small to fairly large, subcircular to irregularly angular, (3–)4.5–12.5 mm diam. or confluent and larger, dark brown to reddish brown, mostly with an indefinite margin, or whitish grey to greyish brown, with a narrow to wide dark brown margin on the adaxial surface, occasionally confined by veins. ***Caespituli*** amphigenous, scattered to dense, dark brown. ***Mycelium*** mainly internal, but some external hyphae also present. External hyphae septate, brown, 2–3.5 μm wide, smooth. **Stromata** often well-developed, up to 50 μm diam., in substomatal chambers or in the mesophyll, brown to dark brown. ***Conidiophores*** in loose to moderately dense fascicles formed by 3–20 conidiophores, arising from internal hyphae or stromata breaking through the adaxial epidermis of the leaves, or penetrating through stomatal openings, or solitary, erect, straight to 1–4 times geniculate or subcylindrical, sometimes branched, mostly attenuated towards the tips that are often irregularly shaped or conical, (12.5–)26–160(–200) × (3.5–)4–5(–5.5) μm, 0–7(–9)-septate, brown to dark brown. ***Conidiogenous cells*** terminal or rarely intercalary, proliferating sympodially, mostly polyblastic, frequently distinctly subdenticulate, sometimes with bent tips looking like a beak or a hook; loci (1.5–)2–2.5(–3) μm wide, thickened and darkened. ***Conidia*** solitary, acicular to narrowly obclavate, straight to curved, (28–)40–265(–280) × (3–)3.5–4.5(–5) μm, 1–9-septate, hyaline, smooth, tip acute, base truncate to obconically truncate, sometimes long obconically truncate, 2–2.5(–3.5) μm wide, hila thickened and darkened.

##### Additional specimen examined.

Benin. Borgou: Parakou, c. 395 m a.s.l., 9°21'27"N, 2°36'44"E, on *Vigna unguiculata*, 17 Sep 2019, Y. Meswaet and R. Dramani, YMM03C (***Paratypes***: M-0312653; UNIPAR).

##### Herbarium specimens examined for comparison.

See Cercospora aff. canescens.

##### Host and distribution.

On *Vigna unguiculata* (Fabaceae) in Benin.

##### Notes.

The infection of leaves of *Vigna unguiculata* by *Cercospora rhynchophora* was severe and caused dark brown to reddish brown large patches (Fig. 2G). This infection was frequently associated with an infection by *Pseudocercospora cruenta* (Sacc.) Deighton. Seven species of *Cercospora* have previously been recorded on *Vigna* spp. (Table 4). Among these, *C. apii*, *C. canescens*, *C. kikuchii* and *C. vignigena* have to date been reported as agents of leaf spot diseases on *V. unguiculata*. Morphologically, *C. rhynchophora* differs from these species by a specific combination of characteristics (Table 4). *C. apii* has often small or no stromata, forms non-geniculate, densely fasciculate and longer conidiophores (20–300 μm) that are uniform in colour and width and carry monoblastic conidiogenous cells (Chupp 1954; Hsieh and Goh 1990) versus developed stromata, shorter conidiophores [(12.5–)26–160(–200) μm] that are irregularly shaped with polyblastic conidiogenous cells presenting beak-shaped tips in *C. rhynchophora*. Additionally, *C. apii* has pale to olivaceous brown conidiophores (Hsieh and Goh 1990) versus the dark brown ones of *Cercospora rhynchophora*.

**Table 4.** Comparison of *Cercospora rhynchophora* (YMM03B) on *Vigna unguiculata*, *Cercospora tentaculifera* (YMM75) on *Vigna unguiculata* as well as on *Phaseolus vulgaris* and *C. vignae-subterraneae* (YMM293, see below) on *Vigna subterranea* with *Cercospora* species known from *Vigna* spp. based on literature ^a–f^.

*Cercospora* species	*Leaf spots*, colour, size	Stromata	Conidiophore size (in μm), branching, septa, colour	Conidium sizes (in μm), septa
***Cercospora rhynchophora*** (YMM03B)	Dark brown to reddish brown, (3–)4.5–12.5 mm diam.	Well-developed	(12.5–)26–160(–200) × (3.5–)4–5(–5.5), branched, 0–7(–9)-septate, dark brown	(28–)40–265(–280) × (3–)3.5–4.5(–5), 1–9 distinct septa
***C. tentaculifera* (YMM75)**	Almost absent	Small or lacking	(32.5–)40–400(–435) × (3–)3.5–4.5(–5), rarely branched, (2–)3–8(–10)-septate, brown to dark brown	(29–)38–188(–240) × (2.5–)3–3.5(–4.5), 1–9 septa
***C. vignae*-*subterraneae*** (YMM293)	Brown to reddish brown, 2–6.5 mm diam.	Lacking or small	(28–)35.5–278(–340) × (3.5–)4–5, rarely branched, 2–6-septate, brown to dark brown	(19–)26.5–100(–110.5) × (2.5–)3–4, (2–)3–6 septa
*C. apii* ^ab^	Present	Often small or lacking, occasionally developed, up to 50 μm diam.	20–300 × 4–6.5, rarely branched, multi-septate, pale brown, uniform in colour and width	25–315 × 3–6, (0–)3–25(–30) septa^b^
*C. canescens* ^a^	3–15 mm	Often small	20–200 × 3–6.5, rarely branched, multi-septate, pale to medium dark brown	25–300 × 2.5–5.5, indistinctly multi-septate
*C. canscorina* ^c^	Pale brown to brown, 3–6 mm	Developed	29.8–85.0 × 3.4–4.2, 1–3-septate, or rarely non-septate, pale brown	31.2–89.9 × 3–3.4, 3–9 septa
*C. caracallae* ^d^	Present	Present	40–80 × 5–6, unbranched,	50–75 × 4, 3–5 septa
*C. kikuchii* ^a^	Present	Small	45–200 × 3–6.5, unbranched, multi-septate	50–375 × 2.5–5, indistinctly multi-septate
*C. longispora* ^e^	Present	Small	5–30 × 1.5–3, unbranched, multi-septate, scars indistinct or lacking	75–170 × 2–3.5, indistinctly multi-septate
*C. vignigena* ^f^	Pale to medium brown, 8–20 mm	Small to well-developed (up to 60 μm diam.)	40–130 × 5–7(–10), 0–3-septate	(35–)45–70(–150) × (2.5–)4–6(–10), (3–)4–7(–14) septa

^a^Hsieh and Goh (1990), ^b^Crous and Braun (2003), ^c^Chiddarwar (1959), ^d^Spegazzini (1910), ^e^Chupp (1954), ^f^Groenewald et al. (2013).

*C. canescens* causes different leaf spots and caespituli, develops small or no stromata and paler conidiophores that are uniform in colour with often monoblastic, mostly uniform conidiogenous cells (Chupp 1954; Hsieh and Goh 1990) versus irregularly shaped conidiophores with polyblastic, beaked conidiogenous cells in *C. rhynchophora*. The distinctness is also confirmed by molecular data. *C. canscorina* forms shorter conidiophores [29.8–85.0 µm versus (12.5–)26–160(–200) μm in *C. rhynchophora*] and conidia [31.2–89.9 × 3–3.4 μm versus (28–)40–265(–280) μm in *C. rhynchophora*] with pale brown and 1–3-septate conidiophores (Chiddarwar 1959). *C. caracallae* has densely fasciculate, unbranched, shorter and wider conidiophores [40–80 × 5–6 µm versus (12.5–)26–160(–200) μm in *C. rhynchophora*] and shorter conidia [50–75 μm versus (28–)40–265(–280) μm of *C. rhynchophora*] with 3–5 septa (Spegazzini 1910). *C. kikuchii* has unbranched conidiophores and longer conidia [50–375 μm versus (28–)40–265(–280) μm in *C. rhynchophora*] that are 0–22-septate (Hsieh and Goh 1990). *C. longispora* has shorter and narrower conidiophores [5–30 × 1.5–3 µm versus (12.5–)26–160(–200) × (3.5–)4–5(–5.5) μm in *C. rhynchophora*] and shorter conidia (75–170 µm versus (28–)40–265(–280) in *C. rhynchophora*] (Chupp 1954). *C. vignigena* produces pale brown and wider conidiophores [5–7(–10) µm versus (3.5–)4–5(–5.5) μm in *C. rhynchophora*] that are 0–3-septate and shorter as well as wider conidia [(35–)45–70(–150) × (2.5–)4–6(–10) μm versus (28–)40–265(–280) × (3–)3.5–4.5(–5) μm of *C. rhynchophora*] (Groenewald et al. 2013).

In the multi-gene (Fig. 1) and the ITS tree (see Suppl. material 3), *C. rhynchophora* forms part of a polytomy with a relatively large genetic distance (branch length) in relation to other sequences considered in the analysis. According to a MegaBLAST search using the *tef1* sequence, the closest matches in NCBI’s GenBank nucleotide database were *Cercospora beticola* Sacc. on *Tetragonia tetragonoides* (Pall.) Kuntze (Aizoaceae) from Brazil (GenBank MN517124; Identities 272 / 279, i.e., 97%), *Cercospora kikuchii* on *Platostoma palustre* (Blume) A.J. Paton (Lamiaceae) from Taiwan (GenBank LC488192; Identities 272 / 279, i.e., 97%) and *Cercospora* sp. RF5 on *Brunfelsia hopeana* (Hook.) Benth. (Solanaceae) from Thailand (GenBank AB863025; Identities 272 / 279, i.e., 97%).

**Figure 9.**
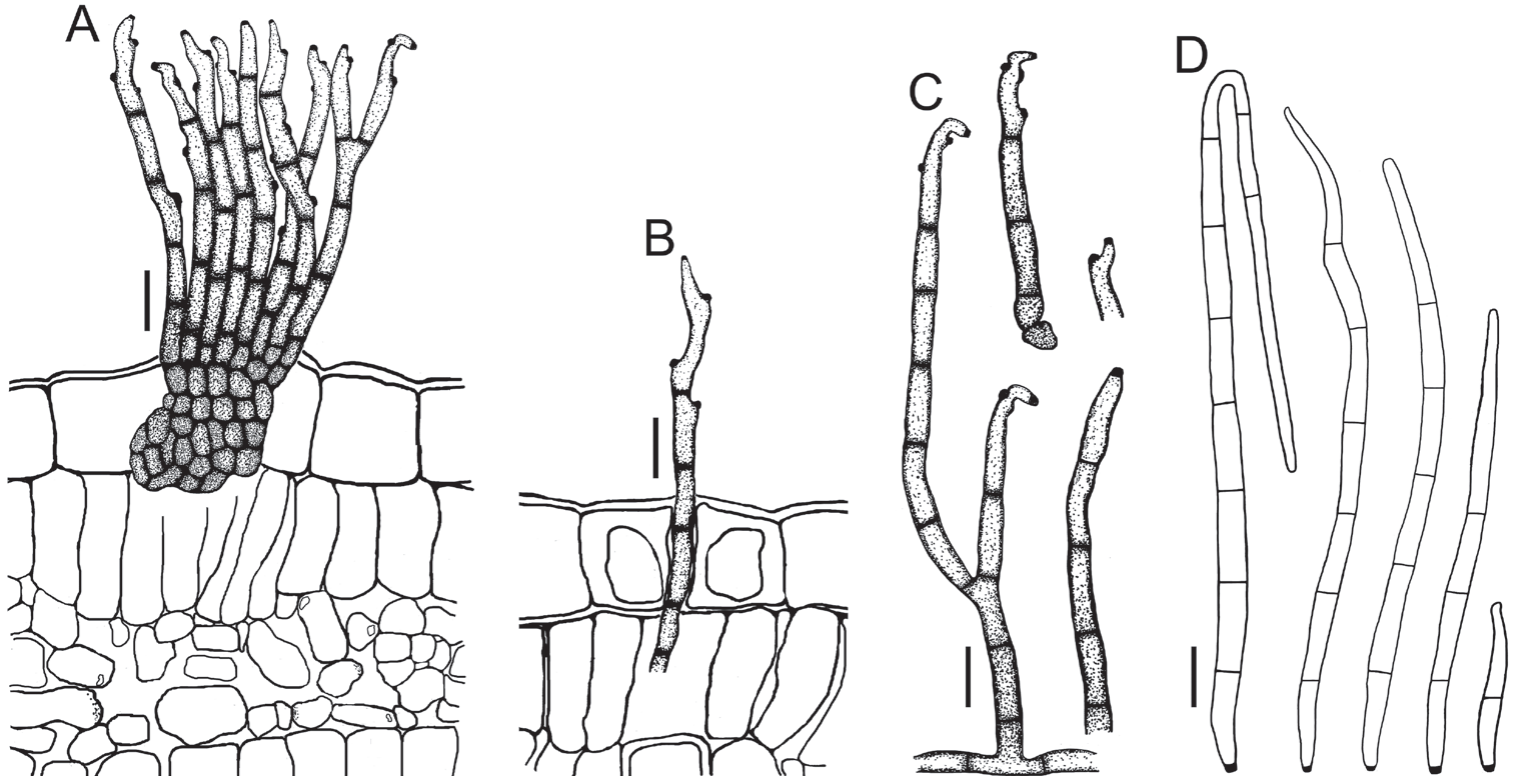
*Cercospora rhynchophora* on *Vigna unguiculata* (YMM03B) **A** fascicle of conidiophores growing out from a developed stroma embedded in the mesophyll **B** conidiophore penetrating through a stomatal opening **C** solitary conidiophores arising from external hyphae **D** conidia. Scale bars: 20 μm (**A, B**); 15 μm (**C, D**).

#### Cercospora

Taxon classificationFungiMycosphaerellalesMycosphaerellaceae

sp. YMM297B on Phaseolus lunatus L.

76794084-DC54-5B7D-8115-A2890DB9B329

Fig. 10

##### Description.

***Leaf spots*** almost lacking to well-developed, amphigenous, subcircular to irregularly angular, 2.5–8 mm diam., reddish brown, later dark brown by abundant caespituli, finally sometimes greyish brown to dark reddish brown, surrounded by dark margins, often with diffuse whitish centres. ***Caespituli*** amphigenous, greyish brown to dark brown. ***Mycelium*** mainly internal. External hyphae branched, 2–3(–4) μm wide, septate, olivaceous brown to brown, smooth. **Stromata** lacking or small, up to 20 μm diam., immersed in the mesophyll or in substomatal cavities, subcircular to irregular, olivaceous brown to darker brown. ***Conidiophores*** in small and loose fascicles, breaking through the adaxial epidermis of the leaves or penetrating through stomatal openings, sometimes solitarily arising through stomatal openings, erect, straight to sinuous, or somewhat geniculate, unbranched, (13–)17.5–195(–220) × (3.5–)4–5 μm, with 2–6(–8) septa each, occasionally slightly constricted and darker at the septa, brown to dark brown. ***Conidiogenous cells*** integrated, terminal, mainly monoblastic; loci 2–3.5 μm wide, thickened and darkened. ***Conidia*** solitary, narrowly obclavate to subacicular, straight to curved, (27–)36–148(–164) × (2.5–)3–4(–4.5) μm, with 2–7(–9) somewhat indistinct septa each, hyaline to sub-hyaline, smooth, apex subacute or acute, base truncate to short obconically truncate, 2–3(–3.5) μm wide, hila thickened and darkened.

##### Specimen examined.

Benin. Borgou: Parakou, Tankaro, c. 360 m a.s.l., 9°23'01"N, 2°30'36"E, on *Phaseolus lunatus*, 20 Sep 2019, Y. Meswaet and R. Dramani, YMM297B (M-0312654; UNIPAR).

##### Notes.

The infection of leaves of *Phaseolus lunatus* by *Cercospora* sp. YMM297B was associated with the infection by *Pseudocercospora griseola*. Among the *Cercospora* spp. known on *Phaseolus* and *Vigna*, *C. olivascens* is morphologically close to *Cercospora* sp. YMM297B. *C. olivascens*, however, differs from *Cercospora* sp. YMM297B by hypophyllous caespituli, no external hyphae, conidiophores that are up to five times geniculate and paler (Saccardo 1878; Chupp 1954), as well as hyaline conidia. The present specimen from Benin presents amphigenous caespituli, external hyphae, less geniculate and brown to dark brown conidiophores and often sub-hyaline conidia. *C. olivascens* also differs from the present species by being originally described from *Aristolochia clematitis* (Aristolochiaceae). According to Chupp (1954), this species was wrongly reported on *Phaseolus vulgaris* by Saccardo (1886). This was confirmed by Crous and Braun (2003). In the ITS phylogeny (see Suppl. material 3), *Cercospora* sp. YMM297B forms part of a polytomy with a relatively large genetic distance (branch length) in relation to other sequences considered in the analysis. In the *tef1* phylogeny (see Suppl. material 4), it is not possible to distinguish this collection from several other *Cercospora* spp. As the description and sequence data are obtained only from a single specimen, the data are not sufficient for a final conclusion and the description as a new species. A reliable species characterisation is not possible until more collections become available.

**Figure 10.**
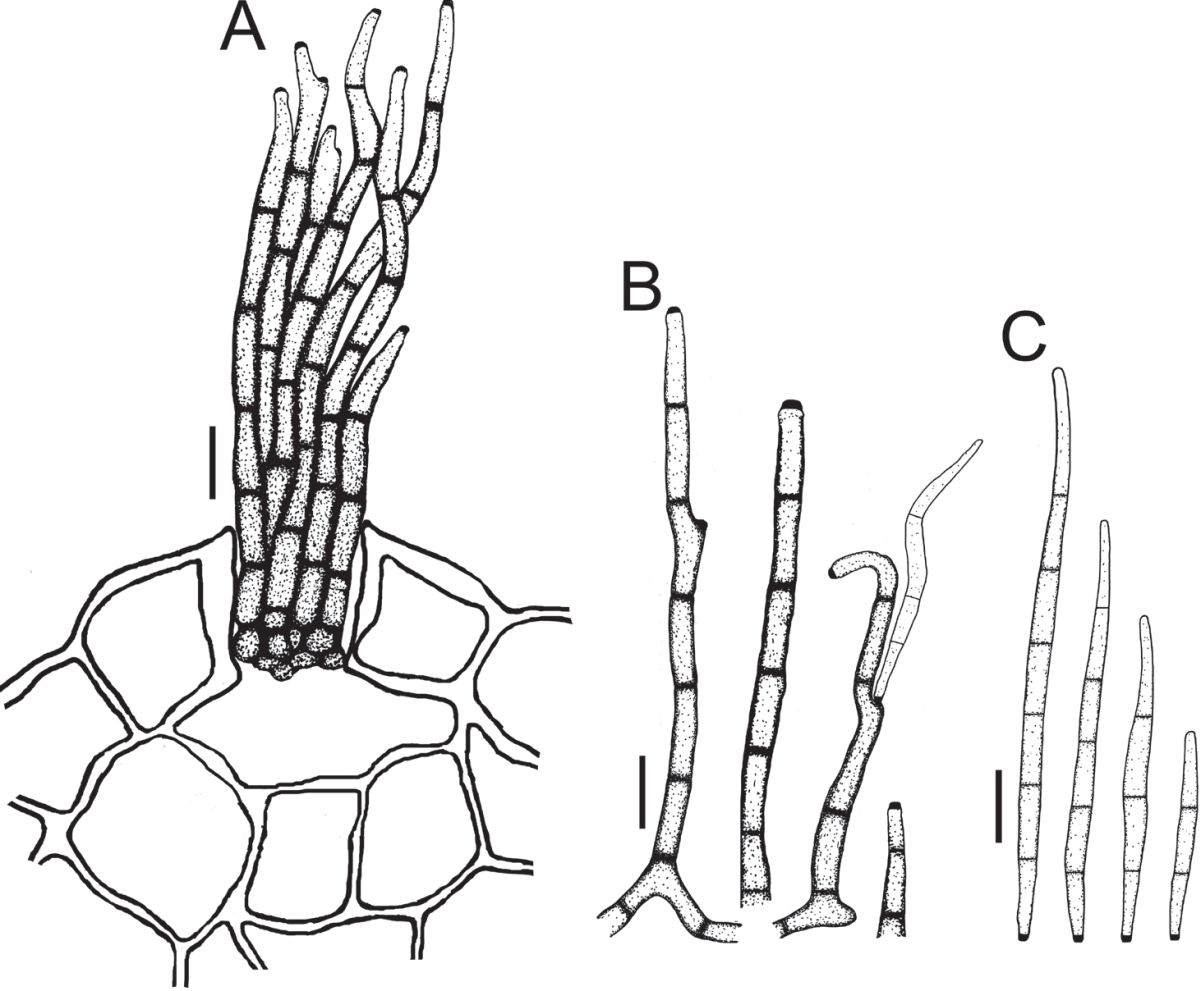
*Cercospora* sp. on *Phaseolus lunatus* (YMM297B) **A** fascicle of conidiophor**e**s emerging through a stomatal opening **B** solitary conidiophores **C** conidia. Scale bars: 15 μm (**A**); 10 μm (**B, C**).

#### Cercospora tentaculifera

Taxon classificationFungiMycosphaerellalesMycosphaerellaceae

Y.Meswaet, Mangelsdorff, Yorou & M.Piepenbr. sp. nov.

5CFA5669-325A-5B1F-83BE-0E825609FA0C

839173

Figs 2H , 11

##### Type.

Benin. Borgou: Parakou, c. 372 m a.s.l., 9°21'43"N, 2°36'04"E, on *Vigna unguiculata* (L.) Walp. (Fabaceae), 02 August 2017, Y. Meswaet, M. Piepenbring, N. S. Yorou and participants of the summer school 2017, YMM75 (***Holotype***: M-0312655; ***Isotype***: UNIPAR). ***Ex holotype sequences*.**MW834448 (SSU), MW834440 (ITS), MW848614 (*tef1*).

##### Etymology.

The epithet *tentaculifera* refers to the ramified and flexible hyphae.

##### Diagnosis.

*Cercospora tentaculifera* differs from other *Cercospora* spp. on *Vigna* and *Phaseolus* in causing inconspicuous or no leaf spots, well-developed external hyphae, mainly adaxial caespituli and up to 435 μm long conidiophores that are constricted at the septa.

##### Description.

***Leaf spots*** almost lacking or pale brown with reddish brown discolorations. ***Caespituli*** amphigenous, mostly epiphyllous, scattered, greyish brown to dark brown. ***Mycelium*** internal and external. External hyphae branched, 2–3.5(–4) μm wide, septate, olivaceous brown to brown, smooth. ***Stromata*** lacking or formed by few substomatal swollen hyphal cells, immersed in the mesophyll or in substomatal cavities. ***Conidiophores*** in small, loose fascicles formed by up to approx. 8 conidiophores, breaking through the adaxial epidermis of the leaves or penetrating through stomatal openings, solitary when arising from external hyphae, erect, straight, curved or slightly 1–2 times geniculate, often constricted at septa, rarely branched, (32.5–)40–400(–435) × (3–)3.5–4.5(–5) μm, (2–)3–8(–10)-septate, brown to dark brown. ***Conidiogenous cells*** terminal, rarely subterminal, mostly monoblastic or with few conidiogenous loci; loci mainly apical, sometimes located on the shoulders of geniculations, 2–2.5(–3.5) μm wide, thickened and darkened, refractive, often subcircular or rarely flattened. ***Conidia*** solitary, acicular to narrowly obclavate, straight to curved, (29–)38–188(–240) × (2.5–)3–3.5(–4.5) μm, 1–9-septate, hyaline, smooth, tip acute, base truncate to short obconically truncate, 2.5–3(–3.5) µm wide, hila thickened and darkened.

##### Additional specimen examined.

Benin. Borgou: Parakou, agricultural research site of the University of Parakou, c. 360 m a.s.l., 9°20'10"N, 2°38'53"E, on *Phaseolus vulgaris*, 20 Aug 2017, Y. Meswaet and A. Tabé, YMM130 (***Paratypes***: M-0312656; UNIPAR).

##### Herbarium specimens examined for comparison.

See Cercospora aff. canescens.

##### Hosts and distribution.

Known on *Phaseolus vulgaris* and *Vigna unguiculata* (Fabaceae) from Benin.

##### Notes.

Thirteen *Cercospora* species have previously been recorded on species of *Vigna* or *Phaseolus* (Tables 4, 5).

Among these, *C. apii*, *C. canescens* and *C. phaseolicola* have a morphology similar to the present collections, particularly by relatively long conidiophores (Tables 4, 5). *C. apii*, however, differs from the present species in causing distinct leaf spots (brown to fairly dark in colour with darker margin), the place of sporulation (caespituli more abundant on the abaxial surface of leaves versus on the adaxial surface of leaves in the case of *C. tentaculifera*), paler and shorter conidiophores [20–300 μm versus (32.5–)40–400(–435) μm in *C. tentaculifera*] that are occasionally arising from developed (up to 50 μm diam.) stromata and somewhat longer and wider conidia [25–315 × 3–6 μm versus (29–)38–188(–240) × (2.5–)3–3.5(–4) μm in *C. tentaculifera*] (Hsieh and Goh 1990). *C. canescens* differs from *C. tentaculifera* in causing different leaf spots and sporulation, producing dense fascicles, paler and shorter conidiophores [20–200 µm versus (32.5–)40–400(–435) µm in *C. tentaculifera*] and somewhat longer conidia [25–300 μm versus (29–)38–188(–240) μm in *C. tentaculifera*] (Hsieh and Goh 1990). *C. phaseolicola* differs from *C. tentaculifera* in causing zonate leaf spots and producing only internal hyphae, hardly geniculate, much longer and wider conidiophores [300–600 × 4–7 µm, occasionally up to 10 μm wide versus (32.5–)40–400(–435) × (3–)3.5–4.5(–5) µm] (Braun et al. 1999).

**Table 5.** Comparison of *Cercospora tentaculifera* (YMM75) on *Vigna unguiculata* and *Phaseolus vulgaris* with *Cercospora* species known from *Phaseolus* spp. based on literature ^a–g^.

*Cercospora* species	*Leaf spots*, colour, size	Stromata	Conidiophore size (in μm), branching, septa, colour	Conidium sizes (in μm), septa
***C. tentaculifera* (YMM75)**	Almost absent	Small or lacking	(32.5–)40–400(–435) × (3–)3.5–4.5(–5), rarely branched, (2–)3–8(–10)-septate, brown to dark brown	(29–)38–188(–240) × (2.5–)3–3.5(–4.5), 1–9 septa
*C. albida* ^a^	Almost absent	Small or lacking	10–60 × 3–6, branched, 1–2-septate	(30–)50–90(–125) × (1.5–)2–3.5(–4), 0–6 septa
*C. canescens* ^b^	3–15 mm	Often small	20-200 × 3–6.5, rarely branched, multi-septate, pale to medium dark brown	25–300 × 2.5–5.5, indistinctly multi-septate
*C. caracallae* ^c^	Present	Present	40–80 × 5–6, unbranched	50–75 × 3–4, 3–5 septa
*C. kikuchii* ^b^	Present	Small	45–200 × 3–6.5, unbranched, multi-septate	50–375 × 2.5–5, indistinctly multi-septate
*C. olivascens* ^d^	Present	Small	50–200 × 4–5.5, unbranched, multi-septate	35–150 × 4–5.5, 3–9 septa
*C. phaseoli-lunati* ^e^	Present	Present	20–100 × 2.5–5(–6), usually pluri-septate	(20–)30–100 × 1–3, pluri-septate
*C. phaseolicola* ^f^	Present	Absent	300–600 × 4–7(–10), branched, pluri-septate	50–200 × 3–5, pluri-septate
*C. phaseolina* ^g^	Present	No information	50–80 × 4–5, unbranched	20–45 × 3–3.5, 1–3 septa
*C. zonata* ^d^	Present	Lacking or slightly developed	10–80 × 3–5, mostly 10–40, 0–2-septate, unbranched	40–125 × 2.5–4.5, usually 3septa

^a^Braun (1995b), ^b^Hsieh and Goh (1990), ^c^Spegazzini (1910)^d^Chupp (1954), ^e^Crous and Braun (2005), ^f^Braun et al. (1999), ^g^Spegazzini (1881).

Based on the present phylogenies, it is not possible to distinguish this species from many other *Cercospora* spp. included in this study. Nevertheless, we propose this species as new to science based on a unique combination of morphological characteristics.

**Figure 11.**
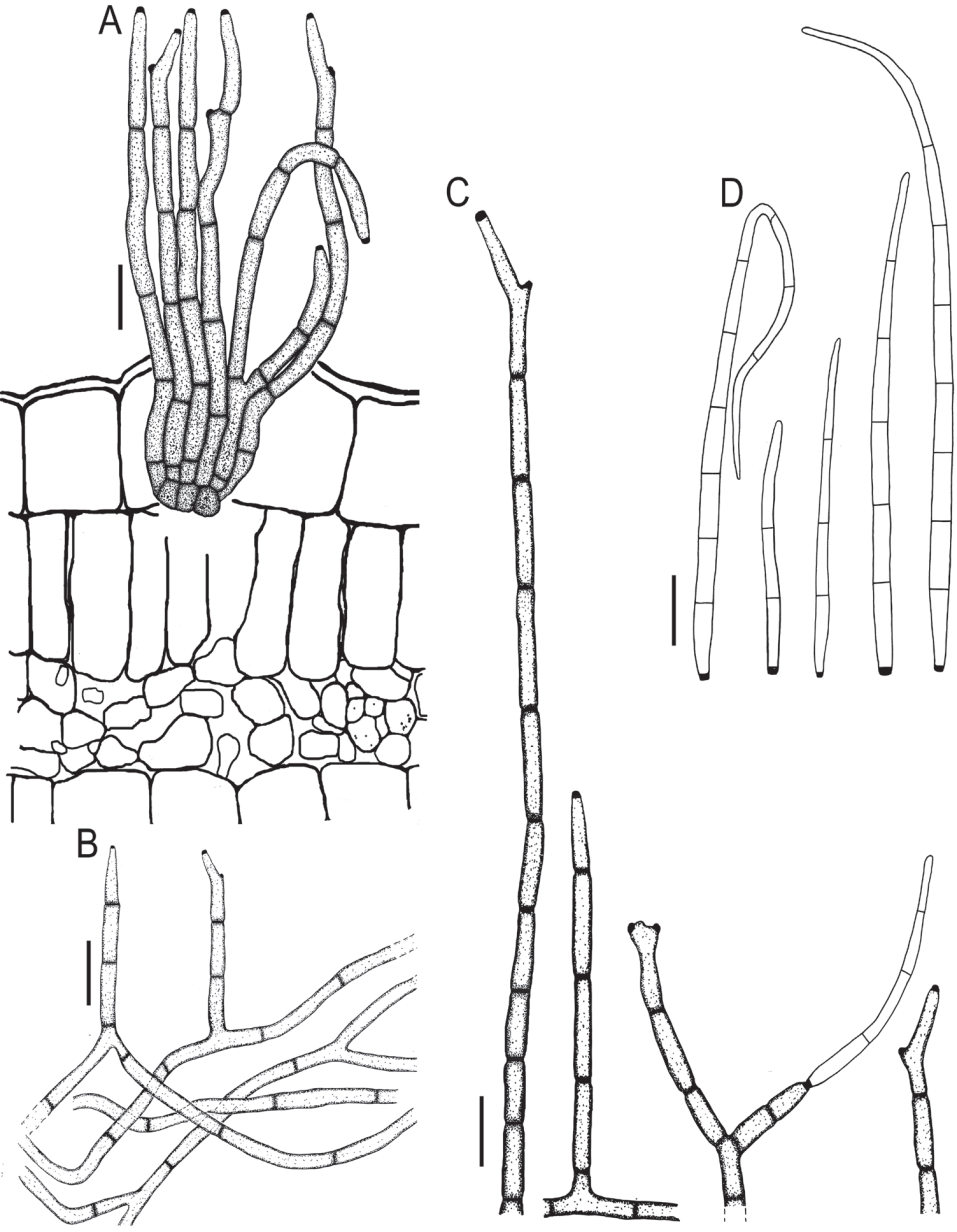
*Cercospora tentaculifera* on *Vigna unguiculata* (YMM75) **A** fascicle of conidiophores growing out from a small stroma immersed in the epidermis **B** external hyphae **C** solitary conidiophores **D** conidia. Scale bars: 20 μm (**A**); 12 μm (**B**); 15 μm (**C, D**).

#### Cercospora vignae-subterraneae

Taxon classificationFungiMycosphaerellalesMycosphaerellaceae

Y.Meswaet, Mangelsdorff, Yorou & M.Piepenbr. sp. nov.

ADABD0A0-02FA-540B-BBE9-8BD842C15413

839174

Figs 2I , 12

##### Type.

Benin. Borgou: Parakou, c. 394 m a.s.l., 9°21'25"N, 2°36'45"E, on *Vigna subterranea* (L.) Verdc. (Fabaceae), 17 Sep 2019, Y. Meswaet and R. Dramani, YMM293 (***Holotype***: M-0312657; ***Isotype***: UNIPAR). ***Ex holotype sequences*.**MW834446 (SSU), MW834438 (ITS), MW848618 (*tef1*).

##### Etymology.

The epithet *vignae-subterraneae* refers to the host species, *Vigna subterranea*.

##### Diagnosis.

*Cercospora vignae-subterraneae* differs from all other *Cercospora* spp. known on *Vigna* spp. in causing often necrotic leaf spots with a pale to white greyish centre, mostly hypophyllous caespituli, external hyphae, flat conidiogenous loci and shorter conidia [(19–)26.5–100(–110.5) µm].

##### Description.

***Leaf spots*** amphigenous, circular or subcircular to irregularly angular, 2–6.5 mm diam., often limited by veins, brown to greyish brown, later necrotic with a pale to white greyish centre, surrounded by a darker margin, the outermost ring mostly darker than the inner margins. ***Caespituli*** amphigenous, but mostly hypophyllous, greyish brown to dark brown. ***Mycelium*** internal and external. External hyphae branched, 2–3(–3.5) μm wide, septate, olivaceous brown to brown, smooth. ***Stromata*** lacking or small, immersed in the mesophyll or in substomatal cavities. ***Conidiophores*** in small to large, loose to dense fascicles or solitary, arising through stomatal openings or breaking through the epidermis, erect, subcylindrical, sinuous or somewhat geniculate, simple or rarely branched, (28–)35.5–278(–340) × (3.5–)4–5 μm, 2–6-septate, smooth, brown to dark brown with slightly paler tips. ***Conidiogenous cells*** terminal, usually monoblastic, rarely polyblastic; loci conspicuous, often flat, (1.5–)2–3 μm wide, darkened and thickened. ***Conidia*** solitary, narrowly obclavate to subacicular, straight to curved, (19–)26.5–100(–110.5) × (2.5–)3–4 μm, (2–)3–6-septate, hyaline, smooth, apex subacute or acute, base truncate to short obconically truncate, (1.5–)2–2.5(–3) µm wide, hila thickened and darkened.

##### Additional specimen examined.

Benin. Alibori: Gogounou, c. 333 m a.s.l., 10°50'35"N, 2°49'42"E, on *Vigna subterranea* Verdc., 2 Sep 2017, Y. Meswaet and A. Tabé, YMM180 (***Paratypes***: M-0312658; UNIPAR).

##### Herbarium specimens examined for comparison.

See Cercospora aff. canescens.

##### Host and distribution.

On *Vigna subterranea* (Fabaceae) in Benin.

##### Notes.

Seven species of *Cercospora* have previously been recorded on *Vigna* spp. (Table 4) (Farr and Rossman 2021). However, no species of *Cercospora* is known to occur on *Vigna subterranea* (Farr and Rossman 2021), a plant species native to West Africa and cultivated mainly in the warm tropics of sub-Saharan Africa (Hepper 1963). Morphologically, *C. vignae-subterraneae* is distinct from all seven species of *Cercospora* mentioned above (Table 4). *C. apii* differs from *C. vignae-subterraneae* by paler conidiophores occasionally arising from a developed stroma of up to 50 μm diam. and above all, longer and wider conidia [25–300 × 3–6 μm versus (19–)26.5–100(–110.5) × (2.5–)3–4 μm in *C. vignae-subterraneae*] that are (0–)3–25(–30)-septate (Hsieh and Goh 1990; Crous and Braun 2003). *C. canescens* causes different leaf spots and caespituli, paler and shorter conidiophores [20–200 µm versus (28–)35.5–278(–340) μm in *C. vignae-subterraneae*] as well as longer conidia [25–300 μm versus (19–)26.5–100(–110.5) μm of *C. vignae-subterraneae*] (Hsieh and Goh 1990). *C. canscorina* forms well-developed stromata as well as paler and shorter conidiophores [29.8–85 µm versus (28–)35.5–278(–340) μm in *C. vignae-subterraneae*] with 1–3 septa (Chiddarwar 1959).

*C. caracallae* has densely fasciculate, unbranched and shorter conidiophores [40–80 µm versus (28–)35.5–278(–340) μm in *C. vignae-subterraneae*] and slightly shorter conidia [50–75 μm versus (19–)26.5–100(–110.5) μm in *C. vignae-subterraneae*] (Spegazzini 1910). *C. kikuchii* has unbranched and shorter conidiophores [45–200 × 3–6.5 µm versus (28–)35.5–278(–340) μm in *C. vignae-subterraneae*] and larger conidia [50–375 μm versus (19–)26.5–100(–110.5) μm in *C. vignae-subterraneae*] with up to 22 septa (Hsieh and Goh 1990). *C. longispora* has unbranched, shorter and narrower conidiophores [5–30 × 1.5–3 µm versus (28–)35.5–278(–340) × (3.5–)4–5 μm in *C. vignae-subterraneae*] with inconspicuous conidiogenous loci and somewhat longer conidia [75–170 × 2–3.5 µm versus (19–)26.5–100(–110.5) μm in *C. vignae-subterraneae*] (Chupp 1954). *C. vignigena* has paler, shorter and wider conidiophores [40–130 × 5–7(–10) µm versus (28–)35.5–278(–340) × (3.5–)4–5 μm in *C. vignae-subterraneae*] that are 0–3-septate, and wider conidia [(2.5–)4–6(–10) μm versus (2.5–)3–4 μm of *C. vignae-subterraneae*) (Mulder and Holliday 1975).

In the multi-gene (Fig. 1) and in the ITS phylogeny (see Suppl. material 3), *C. vignae-subterraneae* forms part of a polytomy with a relatively large genetic distance (branch length) in relation to other sequences considered in the analysis. In the *tef1* phylogeny (see Suppl. material 4), it is not possible to distinguish *C. vignae-subterraneae* from other *Cercospora* spp. Based on the results of our comparative study, we propose *C. vignae-subterraneae* as a species new to science.

**Figure 12.**
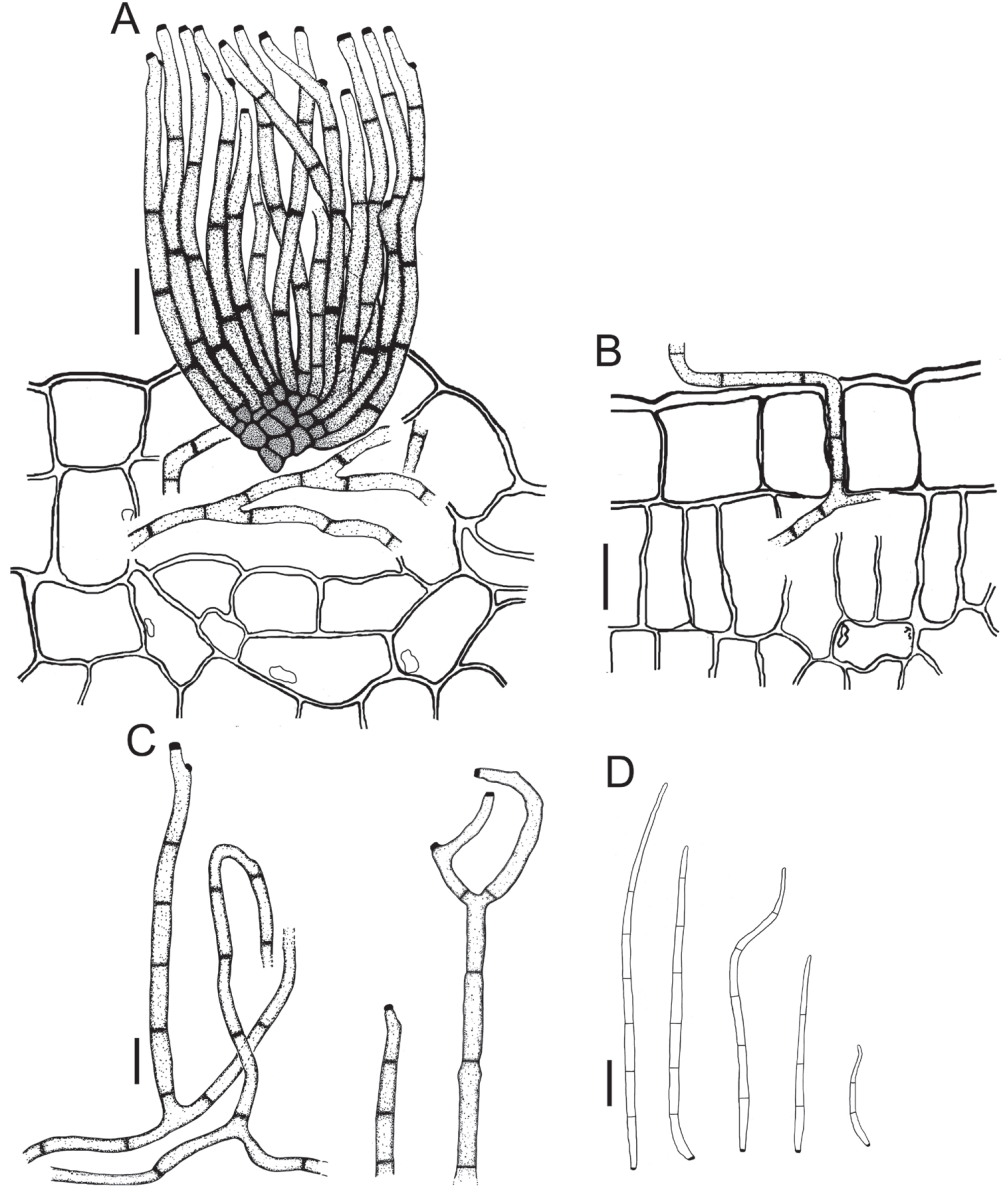
*Cercospora vignae-subterraneae* on *Vigna subterranea* (YMM293) **A** fascicle of conidiophores **B** external hypha penetrating through a stomatal opening **C** solitary conidiophores arising from external hyphae **D** conidia. Scale bars: 20 μm (**A, B**); 12 μm (**C**); 10 μm (**D**).

#### Cercospora zorniicola

Taxon classificationFungiMycosphaerellalesMycosphaerellaceae

Y.Meswaet, Mangelsdorff, Yorou & M.Piepenbr. sp. nov.

D4471312-8E68-5DD6-A7D5-4994E8C8395D

839175

Figs 2J , 13

##### Type.

Benin. Collines: Glazoué, c. 189 m a.s.l., 7°58’ 25"N, 2°14'24"E, on *Zornia glochidiata* DC. (Fabaceae), 22 Sep 2019, Y. Meswaet, A. Tabé and M. Piepenbring, YMM299 (***Holotype***: M-0312659; ***Isotype*s**: UNIPAR) **. *Ex holotype sequences*.**MW848616 (*tef1*).

##### Etymology.

The epithet *zorniicola* refers to the host genus *Zornia* and “-*cola*” (lat. *colere* = to dwell).

##### Diagnosis.

**Figure 13.**
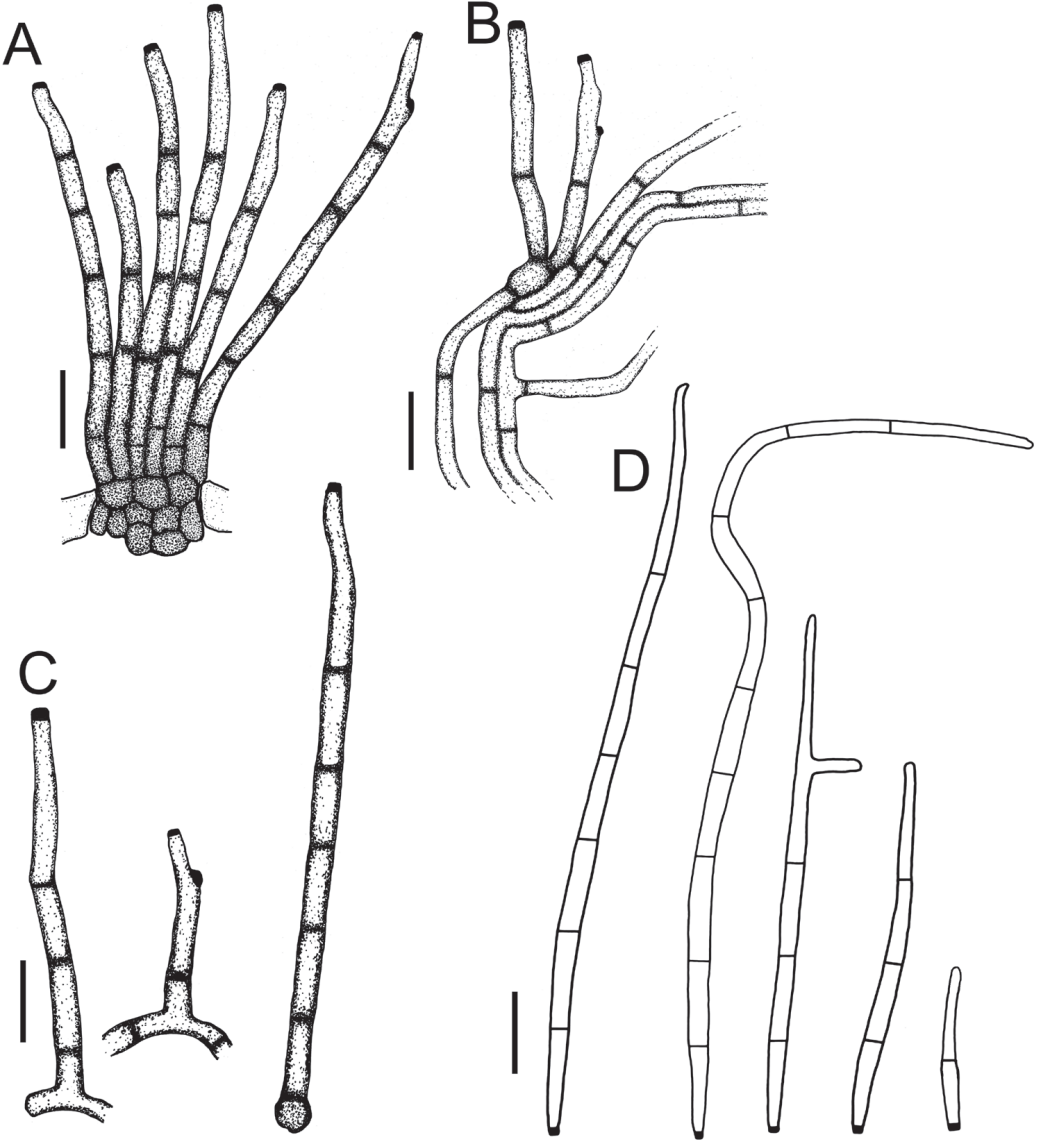
*Cercospora zorniicola* on *Zornia glochidiata* (YMM299) **A** fascicle of conidiophores growing out from a small stroma **B** external hyphae with two conidiophores **C** solitary conidiophores arising from external hyphae **D** conidia. Scale bars: 15 μm (**A**); 12 μm (**B**); 10 μm (**C, D**).

*Cercospora zorniicola* is characterised by external hyphae, unbranched conidiophores that are uniform in colour and width, with mostly monoblastic conidiogenous cells (Fig. 13).

##### Description.

***Leaf spots*** almost lacking or brown to dark brown discolorations, amphigenous, 0.5–2 mm diam., often located along the main veins, surrounded by a yellow discoloration of undefined size and shape. ***Caespituli*** amphigenous, greyish brown to dark brown. ***Mycelium*** internal and external. External hyphae 2–3 μm wide, septate, branched, subhyaline to pale olivaceous, smooth. ***Stromata*** lacking or formed by few substomatal aggregated swollen hyphal cells, up to 22 μm wide, in substomatal chambers or embedded in the mesophyll, dark brown. ***Conidiophores*** in small, loose fascicles of up to approx. 14 conidiophores, arising from internal hyphae breaking through the adaxial epidermis of the leaves, or penetrating through stomatal openings, occasionally solitary arising from external hyphae, erect, straight, subcylindrical to geniculate, unbranched, (15–)24.5–134(–158) × 3.5–4.5 μm, 1–5(–6)-septate, brown to dark brown, often uniform in colour and width. Conidiogenous cells usually monoblastic, rarely polyblastic; loci 1.5–3 μm wide, thickened and darkened. ***Conidia*** solitary, acicular to narrowly obclavate, straight to curved, (15–)27.5–182.5(–200) × (2–)2.5–3.5(–4) μm, 1–8(–12)-septate, hyaline, tip acute, base truncate to short obconically truncate, 1.5–3 µm wide, hila thickened and darkened.

##### Additional specimens examined.

Benin. Borgou: Parakou, on the way to N’Dali, c. 367 m a.s.l., 9°27'53"N, 2°37'43"E, on *Zornia glochidiata*, 17 Sep 2019, Y. Meswaet and R. Dramani, YMM13 (***Paratypes***: M-0312660; UNIPAR). Benin. Borgou: Parakou, c. 391 m a.s.l., 9°22'56"N, 2°37'33"E, same host, 29 Aug 2019, Y. Meswaet and A. Tabé, YMM233 (M-0312661).

##### Notes.

The genus *Zornia* comprises 80 species mainly distributed in tropical regions of the world (Fortuna-Perez et al. 2013). No species of *Cercospora* are currently known on hosts belonging to *Zornia* (Farr and Rossman 2021). *Pseudocercospora zorniae* (J.M. Yen & Gilles) Deighton (≡ *Cercospora zorniae* J.M. Yen & Gilles) is the only known species of cercosporoid fungi infecting species of *Zornia*.

In the multi-gene phylogeny (Fig. 1), *Cercospora zorniicola* grouped closely, but with poor support, with isolates of Cercospora cf. citrullina (MUCC 576) on *Citrullus lanatus* (Thunb.) Matsum. & Nakai (Cucurbitaceae) and *C. kikuchii* on *Glycine max*, *Phaseolus* spp., *Cyamopsis tetragonoloba* (L.) Taub., *Vigna* and other Fabaceae hosts (Mulder and Holliday 1975; Groenewald et al. 2013). However, morphologically, *C. zorniicola* is clearly distinct from C. cf. citrullina by external hyphae, unbranched, darker and longer conidiophores [(15–)24.5–134(–158) μm] and somewhat longer conidia [(15–)27.5–182.5(–200) μm], while C. cf. citrullina has pale to pale brown and short conidiophores (50–86 μm) and shorter conidia (40–130 μm) (Groenewald et al. 2013). *C. zorniicola* differs from *C. kikuchii* in having external hyphae, darker and shorter conidiophores [(15–)24.5–134(–158) μm] and shorter conidia [(15–)27.5–182.5(–200) μm], while *C. kikuchii* has paler and longer conidiophores (45–200 μm) and above all, much longer conidia (50–375 µm) with numerous indistinct septa (Mulder and Holliday 1975; Hsieh and Goh 1990). In the phylogeny based on *tef1* molecular sequence data, it is not possible to distinguish *C. zorniicola* from other *Cercospora* spp. (see Suppl. material 4).

Based on a MegaBLAST search in the NCBI GenBank nucleotide database using the *tef1* sequence data of *C. zorniicola*, the closest matches were Cercospora aff. canescens on *Dioscorea rotundata* Poir. (Dioscoreaceae) from Ghana (GenBank JX143316; Identities 294 / 300, i.e., 98%), Cercospora cf. coreopsidis W.W. Ray on *Coreopsis lanceolata* L. (Asteraceae) form South Korea (GenBank JX143344; Identities 293 / 300, i.e., 97%) and *Cercospora nicotianae* on *Nicotiana tabacum* (Solanaceae) from China (GenBank MK881748; Identities 292 / 300, i.e., 97%). This species is proposed to be new to science based on a distinct combination of morphological characteristics and because no other species of *Cercospora* is currently known on a species of this host genus.

#### Nothopassalora personata

Taxon classificationFungiMycosphaerellalesMycosphaerellaceae

(Berk. & M.A. Curtis) U.Braun, C. Nakash., Videira & Crous, Studies in Mycology 87: 333. 2017.

2D60269B-BAD7-5AD9-89B5-6ABE855016BA

822766

Figs 14A, B , 15

##### Basionym.

*Cladosporium personatum* Berk. & M.A. Curtis, Grevillea 3 (27): 106 (1875).

##### Type.

USA. South Carolina: Santee River, on *Arachis hypogaea* (Fabaceae), (no date), Ravenel 1612 (***Holotype*** K n.v.; ***Isotype*** IMI 104552, n.v.; ***Epitype*** CBS H-22946, n.v.).

For more synonyms see Crous and Braun 2003; Videira et al. 2017 or MycoBank.

##### Description.

***Leaf spots*** amphigenous, subcircular to irregularly angular, 2–8 mm diam., reddish brown, later dark brown by abundant caespituli, finally sometimes greyish brown to blackish brown, margin indefinite. ***Caespituli*** amphigenous, greyish brown to dark brown. ***Mycelium*** mainly internal. **Stromata** small to well-developed, up to 48 μm diam., immersed in the mesophyll or in substomatal chambers, subcircular to irregular, brown to dark brown. ***Conidiophores*** in moderately dense to dense fascicles, arising from stromata, breaking through the adaxial epidermis of the leaves or penetrating through stomatal openings, or solitarily arising through stomatal openings, cylindrical, straight to sinuous or geniculate, conically truncate at the apex, unbranched, (12.5–)20–55.5(–58) × 5–7 μm, 1–3(–4)-septate, pale brown to brown, paler towards the apex. Conidiogenous loci 2.5 μm wide, thickened and darkened. ***Conidia*** solitary, cylindrical to long-obclavate with round apex, straight to curved, (14–)23–68(–80) × (5–)5.5–8(–9) μm, 2–6-septate, pale brown to olivaceous brown, base obconically truncate, 2–3 μm wide, hila thickened and darkened.

##### Specimens examined.

Benin. Donga: Taneka-Koko, c. 441 m a.s.l., 9°51'30"N, 1°29'34"E, on *Arachis hypogaea*, 29 Jul 2017, Y. Meswaet, M. Piepenbring N. S. Yorou and participants of the summer school 2017, YMM49A (M-0312662; UNIPAR). Benin. Borgou: Parakou, c. 354 m a.s.l., 9°20'02"N, 2°38'48"E, same host, 27 Aug 2019, Y. Meswaet and R. Dramani, YMM224A (M-0312663). Benin. Borgou: Parakou, Songhai (farm school), c. 333 m a.s.l., 9°24'42"N, 2°41'24"E, same host, 30 Aug 2019, Y. Meswaet and A. Tabé, YMM247 (M-0312664). Benin. Borgou: Commune of Nikki, Tontarou, c. 452 m a.s.l., 9°50'23"N, 3°14'59"E, same host, 19 Sep 2019, Y. Meswaet, A. Tabé and M. Piepenbring, YMM295 (M-0312665).

##### Herbarium specimens examined for comparison.

*Nothopassalora personata*. On *Arachis* sp.: Democratic Republic of the Congo (Zaire). Kindu, 28 Jan 1920, Shantz H. L., 628 (BPI 439440 as *Cercospora personata* Berk. & M.A. Curtis). On *Arachis* sp.: Guinea. 5 Sep 1964, Litzenberger S. C., 91 (BPI 439443 as *C. personata*). On *A. hypogaea*: Indonesia, Java, Tegal, 28 Jan 1920, Raciborski, s.n (BPI 407235 “type?” of *Septogloeum arachidis* Racib.).

##### Hosts and distribution.

On *Arachis glabrata* Benth., *A. hypogaea* (Fabaceae), known in tropical regions where the host is cultivated, including Afghanistan, Angola, Argentina, Australia, Azerbaijan, Bangladesh, Barbados, Benin, Bermuda, Bhutan, Bolivia, Brazil, Brunei, Burkina Faso, Cambodia, Canada, China, Cambodia, Cameroon, Chad, Colombia, Congo, Cuba, Dominican Republic, Egypt, EI Salvador, Ethiopia, Fiji, French Polynesia, Gabon, Gambia, Georgia, Ghana, Greece, Guam, Guatemala, Guinea, Guyana, Haiti, Honduras, Hong Kong, India, Indonesia, Iran, Iraq, Israel, Ivory Coast, Jamaica, Jordan, Kenya, Korea, Laos, Lesser Antilles, Liberia, Libya, Madagascar, Malawi, Malaysia, Mali, Mauritius, Mexico, Morocco, Mozambique, Myanmar, Nepal, New Caledonia, Nicaragua, Niger, Nigeria, Pakistan, Panama, Papua New Guinea, Paraguay, Peru, Philippines, Puerto Rico, Russia, Saint Vincent and the Grenadines, Senegal, Sierra Leone, Singapore, Solomon Islands, Somalia, South Africa, Spain, Sri Lanka, Sudan, Suriname, Taiwan, Tanzania, Thailand, Togo, Tonga, Trinidad and Tobago, Turkmenistan, Turkey, Uganda, Uruguay, USA, Uzbekistan, Venezuela, Vietnam, Zambia, Zimbabwe (Yen and Lim 1980; Hsieh and Goh 1990; Shin and Kim 2001; Crous and Braun 2003; Farr and Rossman 2021).

**Figure 14.**
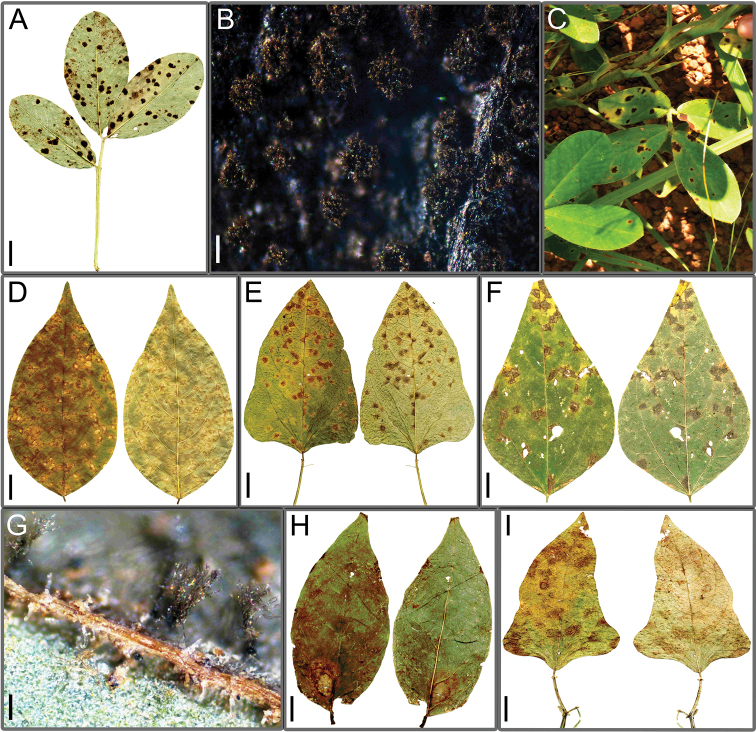
Leaf spot symptoms associated with cercosporoid fungi **A, B***Nothopassalora personata* on *Arachis hypogaea* (YMM49A) **B** close-up of lesions with caespituli **C***Passalora arachidicola* on *Arachis hypogaea* (YMM49B) **D***Pseudocercospora bradburyae* on *Centrosema pubescens* (YMM275) **E***Pseudocercospora cruenta* on *Phaseolus* sp. (YMM288) **F, G***Pseudocercospora griseola* on *Phaseolus lunatus* (YMM297A) **G** close-up of lesions with sporulation **H***Pseudocercospora sennicola* on *Senna occidentalis* (YMM12) **I***Pseudocercospora tabei* on *Vigna unguiculata* (YMM220). Scale bars: 15 mm (**A, D, E, F, I**); 100 μm (**B, G**); 12 mm (**D, H**).

##### Notes.

*Nothopassalora personata* and *Passalora arachidicola* (Hori) U.Braun are the agents of the two major foliar diseases of *Arachis hypogaea* worldwide (Jenkins 1938; Kokalis-Burelle et al. 1997; Videira et al. 2017). During the collecting activities in Benin, we observed that both, *N. personata* and *P. arachidicola*, are present wherever *A. hypogaea* is grown and mixed infections are common. In addition, *N. personata* is occasionally associated with *Puccinia* sp. *N. personata* often predominates and is more destructive than *P. arachidicola*. *N. personata* differs from *P. arachidicola*, in forming wider conidiophores (5–7 μm) as well as cylindrical and wider conidia [(5–)5.5–8(–9) µm], while *P. arachidicola* forms narrower conidiophores [(3.5–)4–5 µm)] and conidia (3.5–4.5 μm). *N. personata* and *P. arachidicola* on *A. hypogaea* were previously reported from Benin (Crous and Braun 2003), but this is the first report of these pathogens in Benin including details of the two species.

**Figure 15.**
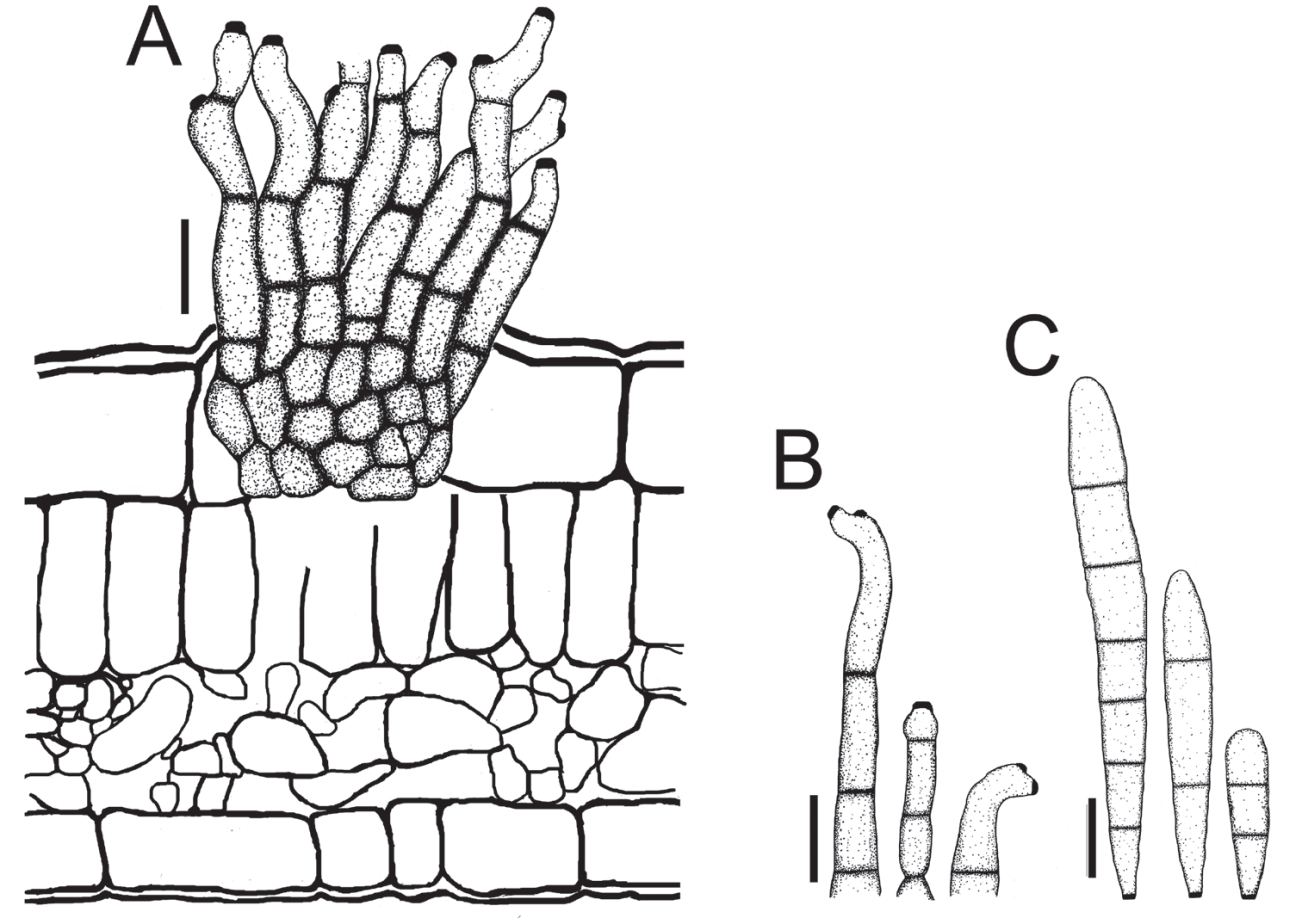
*Nothopassalora personata* on *Arachis hypogaea* (YMM49A) **A** fascicle of conidiophores growing out from a developed stroma embedded in the epidermis **B** solitary conidiophores **C** conidia. Scale bars: 15 μm (**A**); 12 μm (**B, C**).

#### Passalora arachidicola

Taxon classificationFungiMycosphaerellalesMycosphaerellaceae

(Hori) U.Braun, New Zealand J. Bot. 37: 303. 1999.

9B59EDBB-2919-5E66-8725-548783FF14B0

459582

Figs 14C , 16

##### Basionym.

*Cercospora arachidicola* Hori, Rep. (Annual) Nishigahara Agric. Exp. Sta. Tokyo: 26. 1917.

##### Type.

Japan. Tokyo, Experiment Station, on *Arachis hypogaea* (Fabaceae), (no date), S. Hori. s.n. (***Holotype*** HIRO, n.v.).

For more synonyms see Crous and Braun (2003) or MycoBank.

##### Description.

***Leaf spots*** amphigenous, subcircular to angular-irregular, 2.5–9.5 mm diam., greyish brown to medium dark brown, occasionally limited by veins, margin indefinite. ***Caespituli*** epiphyllous, whitish brown to greyish brown. ***Mycelium*** mainly internal. Internal hyphae pale brown, smooth, 1.5–3 μm wide. ***Stromata*** small, up to approx. 32 μm diam., embedded in the mesophyll or in substomatal chambers, subcircular to irregular, brown to dark brown. ***Conidiophores*** in small, loose to moderately dense fascicles, arising from internal hyphae or stromata, or solitary, arising through stomatal openings, erect, straight to sinuous or geniculate, simple, (11.5–)14–42.5(–53) × (3.5–)4–5 μm, 0–5-septate, smooth, olivaceous brown to slightly dark brown, paler towards the tips. Conidiogenous loci 2–2.5(–3) μm wide, thickened and darkened. ***Conidia*** solitary, narrowly obclavate to subacicular, straight to slightly curved, (16–)23–76.5(–88) × 3.5–4.5 μm, 2–5-septate, olivaceous brown, apex subacute or acute, base truncate to short obconically truncate, 2–2.5(–3.5) μm wide, hila thickened and darkened.

##### Specimens examined.

Benin. Donga: Taneka-Koko, c. 441 m a.s.l., 9°51'30"N, 1°29'34"E, on *Arachis hypogaea*, 29 Jul 2017, Y. Meswaet, M. Piepenbring, N. S. Yorou and participants of the summer school 2017, YMM49B (M-0312666; UNIPAR). Benin. Borgou: Parakou, c. 354 m a.s.l., 9°20'02"N, 2°38'48"E, same host, 27 Aug 2019, Y. Meswaet and R. Dramani, YMM224B (M-0312667).

##### Herbarium specimens examined for comparison.

*Passalora arachidicola*. On *Arachis* sp.: Guinea. Labe, 29 Jul 1964, Litzenberger S. C. 55 (BPI 432987 as *Cercospora arachidicola*). On *Arachis* sp.: Guinea. Dubreka, 25 Jul 1964, Litzenberger S. C. 39 (BPI 432989 as *C. arachidicola*). On *Arachis* sp.: Guinea. Beyla, 2 Aug 1964, Litzenberger S. C. 47 (BPI 432990A as *C. arachidicola*). On *Arachis* sp.: Guinea. Kissidougou, 4 Aug 1964, Litzenberger S. C. 28 (BPI 432991 as *C. arachidicola*). On *Arachis* sp.: Guinea. Dabola, 4 Aug 1964, Litzenberger S. C. 26 (BPI 432992 as *C. arachidicola*).

##### Host and distribution.

On *Arachis hypogaea* (Fabaceae) known worldwide where the host is cultivated, including Afghanistan, Angola, Argentina, Australia, Bangladesh, Benin, Bolivia, Brazil, Brunei, Burkina Faso, China, Cuba, Cambodia, Cameroon, Colombia, Comoros, Democratic Republic Congo, Cuba, Dominican Republic, El Salvador, Fiji, Gabon, Gambia, Ghana, Guatemala, Guinea, Guyana, Hong Kong, India, Indonesia, Ivory Coast, Jamaica, Japan, Kenya, Korea, Laos, Lebanon, Libya, Madagascar, Malawi, Malaysia, Mali, Mauritius, Mexico, Mozambique, Myanmar, Nepal, New Caledonia, Nicaragua, Niger, Nigeria, Pakistan, Panama, Papua New Guinea, Philippines, Puerto Rico, Malaysia, Senegal, Sierra Leone, Solomon Islands, Somalia, South Africa, Sudan, Suriname, Taiwan, Tanzania, Thailand, Togo, Uganda, USA, Uruguay, Venezuela, Vietnam, Zambia, Zimbabwe (Chupp 1954; Hsieh and Goh 1990; Shin and Kim 2001; Crous and Braun 2003; Farr and Rossman 2021).

##### Notes.

*Passalora arachidicola* was placed into the genus *Passalora* by Braun (1999) based on morphological characteristics that are confirmed in the context of the present study. Crous et al. (2009b, 2009c, 2013a) showed that the genus *Passalora* is paraphyletic or polyphyletic. Therefore, the present species most probably does not belong to *Passalora*. However, we refrain from drawing taxonomic conclusions here because a revision of the genus *Passalora* is beyond the scope of the present study.

**Figure 16.**
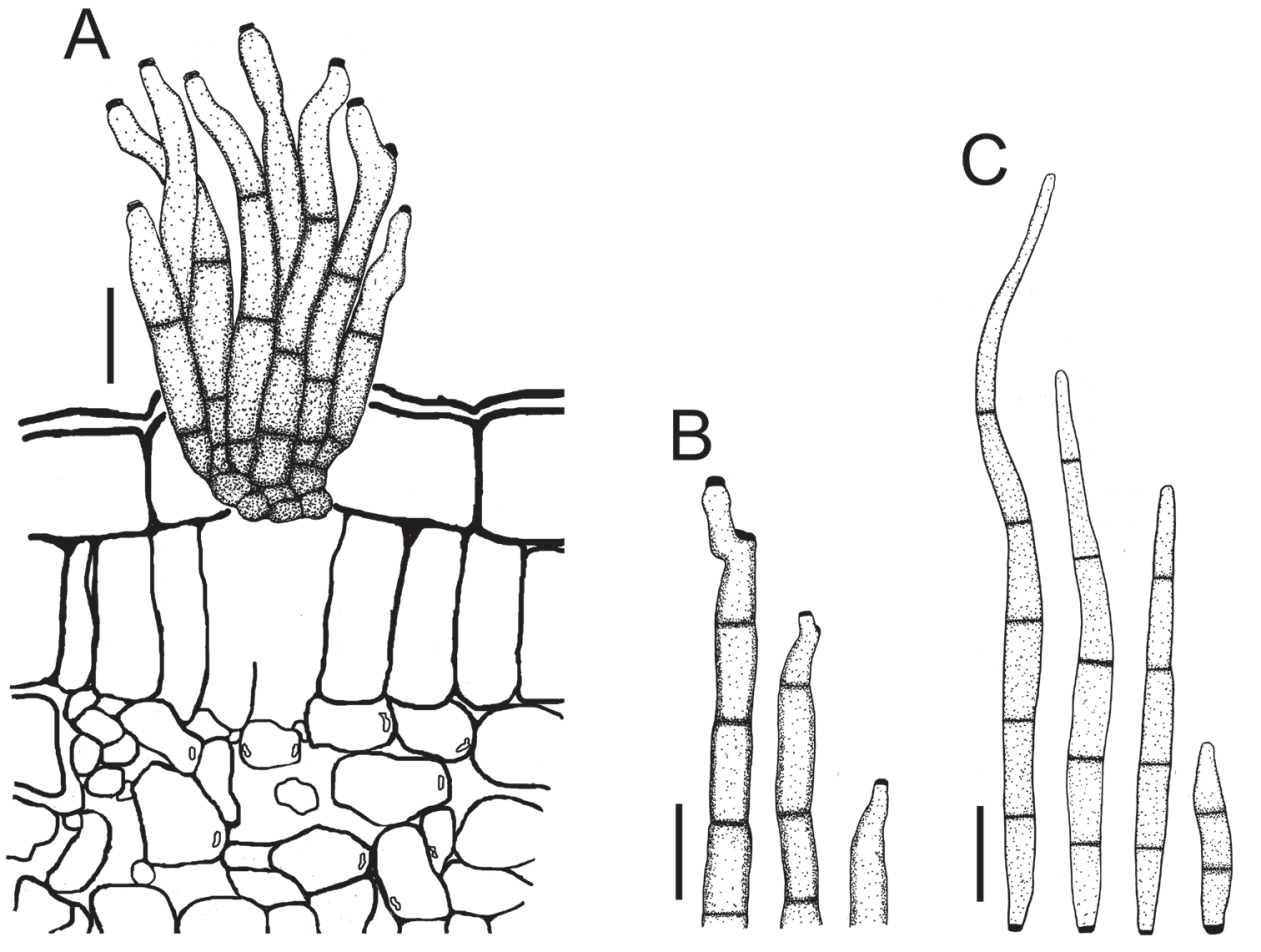
*Passalora arachidicola* on *Arachis hypogaea* (YMM49B) **A** fascicle of conidiophores growing out from a small stroma embedded in the epidermis **B** solitary conidiophores **C** conidia. Scale bars: 15 μm (**A**); 10 μm (**B, C**).

#### Pseudocercospora bradburyae

Taxon classificationFungiMycosphaerellalesMycosphaerellaceae

(E. Young) Deighton, Mycological Papers 140: 140. 1976

50B23A11-1F0F-5D80-8BF1-D28A2EAEE146

321522

Figs 14D , 17

##### Basionym.

*Cercospora bradburyae* E. Young, Mycologia 8 (1): 46 (1916).

##### Type.

Puerto Rico. Rosario, on *Centrosema pubescens* (as *Bradburya pubescens* (Benth.) Kuntze (Fabaceae), 15 Feb 1913, F. L. Stevens 446 (***Holotype***: ILL!).

For more synonyms see Crous and Braun 2003 or MycoBank.

##### Description.

***Leaf spots*** amphigenous, subcircular to irregularly angular, (2.5–)4–8.5 mm diam., limited by veins, reddish brown to brown, with indefinite margins. ***Caespituli*** mainly epiphyllous, olivaceous brown to slightly dark brown. ***Mycelium*** internal and external. External hyphae branched, 2.5–3.5 μm wide, septate, olivaceous brown to brown, smooth. ***Stromata*** lacking or small, about 10–18 μm diam., immersed in the mesophyll or in substomatal chambers. ***Conidiophores*** often in small, loose to slightly dense fascicles of up to approx. 10 conidiophores, arising from stromata or breaking through the adaxial epidermis of the leaves, occasionally solitary arising from external hyphae, straight to sinuous or somewhat geniculate, rarely branched, (11–)13–44(–48.5) × (3.5–)4–5 μm, 0–3(–4)-septate, smooth, olivaceous brown to brown, paler towards the tips. ***Conidiogenous cells*** terminal, 10–15 μm long; loci inconspicuous to distinctly denticle-like, not thickened and not darkened, 1.5–3 μm wide. ***Conidia*** solitary, narrowly obclavate to subacicular, straight to curved, (30–)38–110(–130) × (2.5–)3–4(–4.5) μm, 3–9-septate, olivaceous brown, smooth, apex subacute to rounded and slightly narrower, base truncate to obconically truncate, 1.5–3 μm wide, hila not thickened and not darkened, occasionally somewhat refractive.

##### Specimens examined.

Benin. Borgou: N’Dali, c. 380 m a.s.l., 9°52'33"N, 2°41'20"E, on *Centrosema pubescens*, 31 Aug 2019, Y. Meswaet and A. Tabé, YMM275 (M-0312668; UNIPAR). Same locality and host, 1 Sep 2019, Y. Meswaet and A. Tabé, YMM275B (M-0312669).

##### Herbarium specimens examined for comparison.

*Pseudocercospora bradburyae*. On *Centrosema pubescens* (as *Bradburya pubescens*): Puerto Rico. Rosario, 15 Feb 1913, Stevens F. L. 446 (ILL14818 ***Holotype*** of *Cercospora bradburyae*). Puerto Rico. Mayagüez, 31 Oct 1913, Stevens F. L. 3930 (ILL10600 ***Paratype***). Puerto Rico. San Germán, 12 Dec 1913, Stevens F. L. 5833 (ILL10606 ***Paratype***). Puerto Rico. Dos Bocas, below Utuado, 30 Dec 1913, Stevens F. L. 6558(ILL10603 ***Paratype***). Puerto Rico. Hormigueros, 14 Jan 1914, Stevens F. L. 225a (ILL10609 ***Paratype***). Guinea. Kindia, May 1963, Kranz J, 2795 (BPI 1112168).

##### Host and distribution.

On *Centrosema acutifolium* Benth., *C. arenarium* Benth., *C. brasilianum* (L.) Benth., *C. macrocarpum* Benth., *C. plumieri* Benth., *C. pubescens*, *C. virginianum* (L.) Benth, *Centrosema* spp. (Fabaceae) from Australia, Barbados, Bolivia, Brazil, Brunei, Cambodia, China, Colombia, Costa Rica, Cuba, Dominican Republic, Ecuador, Fiji, Hong Kong, Ghana, Guinea, Indonesia, Jamaica, Malaysia, Mexico, Micronesia, Mona Island, New Caledonia, Nigeria, Niue, Palau, Papua New Guinea, Peru, Philippines, Puerto Rico, Solomon Islands, St. Thomas, South Africa, Taiwan, Thailand, Togo, Tonga, Trinidad and Tobago, Vanuatu, Venezuela, Virgin Islands (Chupp 1954; Ellis 1976; Hsieh and Goh 1990; Crous and Braun 2003; Farr and Rossman 2021). This species is reported here for the first time for Benin.

##### Notes.

Three species of *Pseudocercospora*, namely *Ps. bradburyae*, *Ps. centrosematicola* (J.M. Yen & Lim) J.M. Yen and *Ps. clitoriae* (G.F. Atk.) Deighton are known on *Centrosema* spp. (Chupp 1954; Farr and Rossman 2021). The present specimens from Benin differ from *Ps. clitoriae* by having often small fascicles formed by up to approx. 10 conidiophores and longer conidiophores [(11–)13–44(–48) µm] and wider conidia [(2.5–)3–4(–4.5) µm], while *Ps. clitoriae* has large, dense fascicles formed by 40 or more conidiophores, shorter conidiophores [8–15(–22) µm] and narrower conidia (2.5–3 µm) (Chupp 1954; Deighton 1976). Based on the descriptions made by Chupp (1954), Hsieh and Goh (1990), Young (1916) and the re-examination of the type specimen of *Ps. bradburyae*, the present specimen from Benin agrees well with *Ps. bradburyae*. In the *tef1* phylogeny (see Suppl. material 4), *Ps. bradburyae* grouped with low support with isolates of *Ps. humuli* on *Humulus lupulus* (Cannabaceae) from Japan, *Ps. cercidicola* on *Cercis chinensis* (Fabaceae) from Japan and *Ps. abelmoschi* on *Hibiscus syriacus* (Malvaceae) from South Korea.

**Figure 17.**
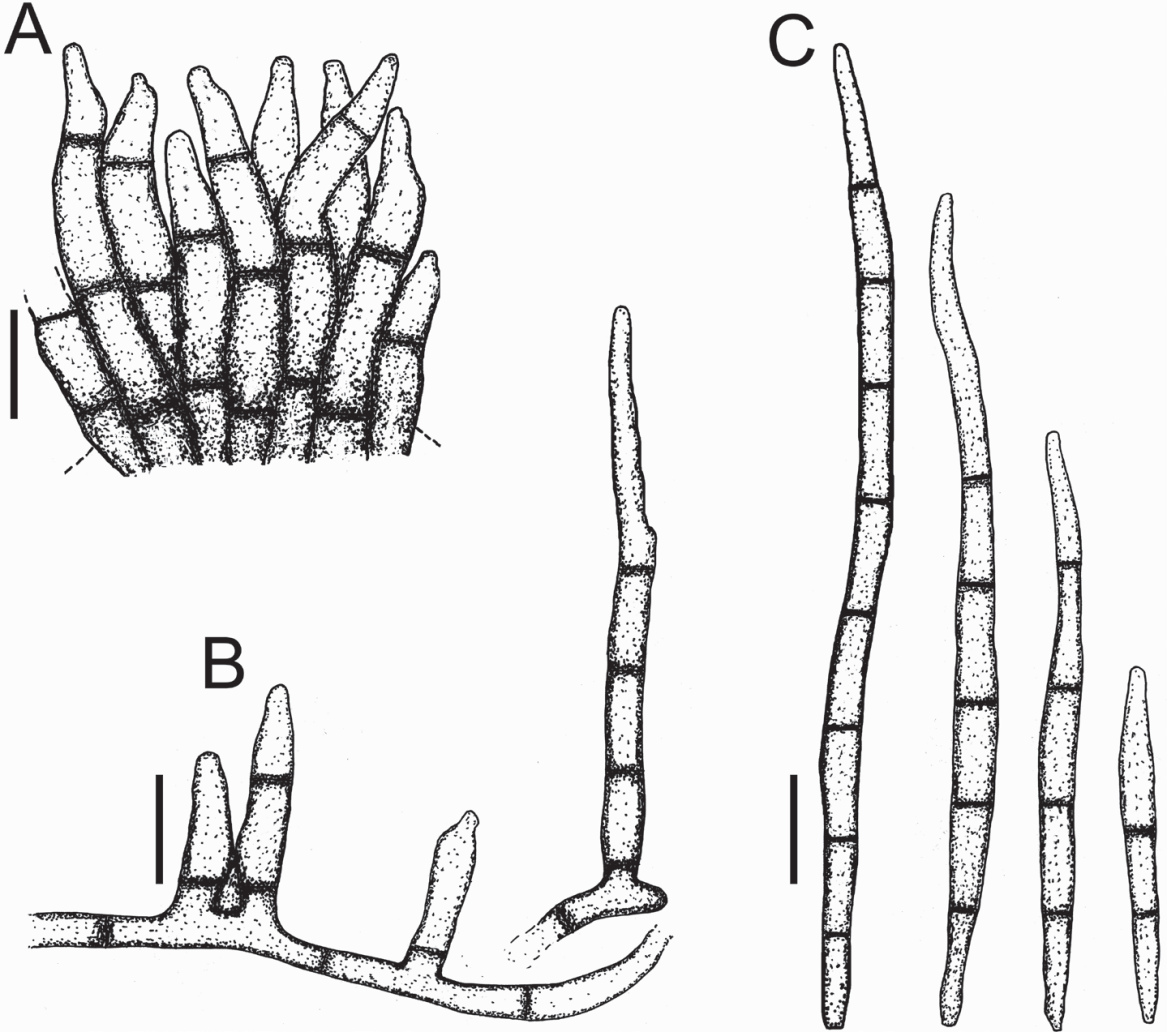
*Pseudocercospora bradburyae* on *Centrosema pubescens* (YMM275) **A** fascicle of conidiophores **B** solitary conidiophores arising from external hyphae **C** conidia. Scale bars: 15 μm (**A**); 10 μm (**B, C**).

#### Pseudocercospora cruenta

Taxon classificationFungiMycosphaerellalesMycosphaerellaceae

(Sacc.) Deighton, Mycol. Pap. 140: 142. 1976

8475F659-D93F-5BF1-98B0-CF500449769D

321556

Figs 14E , 18

##### Basionym.

*Cercospora cruenta* Sacc., Michelia 2:149 (1880).

##### Type.

USA. South Carolina: (no further information on the locality), on *Phaseolus* sp. (Fabaceae), (no date), Ravenel 2156 (***Holotype***: PAD, n.v.).

For more synonyms see Crous and Braun (2003) or MycoBank.

##### Description.

***Leaf spots*** amphigenous, subcircular to irregularly angular, (2.5–)4–8.5 mm diam., limited by veins, reddish brown to dark brown, with an indefinite margin. ***Caespituli*** amphigenous, denser, darker olivaceous to almost sooty on the abaxial surface of the leaves than on the adaxial side. ***Mycelium*** internal and external. External hyphae branched, 2.5–3.5 μm wide, septate, olivaceous brown to brown, smooth. ***Stromata*** lacking or small, 8–14 μm diam., immersed in the mesophyll or in substomatal cavities, subcircular to irregular, brown to dark brown. ***Conidiophores*** in small, loose, moderately large and dense fascicles formed by up to approx. 10 conidiophores, arising from stromata, breaking through the adaxial epidermis of the leaves or penetrating through stomatal openings, sometimes solitary, arising from external hyphae, straight to sinuous or somewhat geniculate, rarely branched, (12–)15.5–54(–58.5) × (3.5–)4–5 μm [in YMM125 up to 120 µm long], 1–3-septate, smooth, olivaceous brown to brown, paler towards the tips. ***Conidiogenous cells*** terminal or subterminal, a conidiophore can be reduced to a single conidiogenous cell; loci 2–2.5 μm wide, not thickened and not darkened. ***Conidia*** solitary, narrowly obclavate to subacicular, straight to curved, (30.5–)42–132(–154) × (3–)3.5–4.5(–5) μm, 2–10-septate, olivaceous brown, smooth, apex subacute to rounded and slightly narrower than the rest of the conidiophore, up to 2.5 μm wide, base truncate to obconically truncate, 2–2.5(–3) μm wide, hila not thickened and not darkened.

##### Specimens examined.

Benin. Borgou: Parakou, c. 353 m a.s.l., 9°20'02"N, 2°38'48"E, on *Phaseolus* sp., 12 Sep 2019, Y. Meswaet and A. Tabé, YMM288 (M-0312670, UNIPAR). Benin. Atlantique: Commune of Allada, Sékou, c. 84 m a.s.l., 6°38'18"N, 2°13'09"E, on *Vigna unguiculata*, 15 August 2017, Y. Meswaet and A. Tabé, YMM125 (M-0312671; UNIPAR). Benin. Borgou: Parakou, c. 385 m a.s.l., 9°20'34"N, 2°36'39"E, same host, 14 Sep 2019, Y. Meswaet and R. Dramani, YMM03A (M-0312672). Borgou: Parakou, c. 394 m a.s.l., 9°21'25"N, 2°36'45"E, same host, 17 Sep 2019, Y. Meswaet and R. Dramani, YMM294B (M-0312673). Benin. Borgou: Parakou, c. 363 m a.s.l., 9°20'29"N, 2°37'28"E, same host, 21 Sep 2019, Y. Meswaet and A. Tabé, YMM04 (M-0312674).

##### Herbarium specimens examined for comparison.

*Pseudocercospora cruenta*. On *Vigna unguiculata*: USA. Mississippi: Starkville, Sep 1888, Tracy S. M. s.n. (BPI 435817 ***Paratype*** of *Cercospora dolichi* Ellis & Everh.); On *Phaseolus* sp.: USA. South Carolina: Aiken, no date, Ravenel H. W. s.n (BPI 439619 ***Paratype*** of *C. phaseolorum* Cooke). *Pseudocercospora stizolobii* (Syd. & P. Syd.) Deighton. On *Mucuna* sp.: Philippines. Los Baños, 6 Apr 1913, Raimundo M. B. 892 (BPI 441666 ***Holotype*** of *C. stizolobii* Syd. & P. Syd.).

##### Hosts and distribution.

On *Calopogonium* sp., *Canavalia ensiformis* (L.) DC., *C. gladiata* (Jacq.) DC., *C. maritima* Thouars, *Canavalia* sp., *Cassia lathyroides* L., *Cicer arietinum* L., *Clitoria ternatea* L., *Dolichos biflorus* L., *D. lablab* L., *Dolichos* sp., *Glycine max*, *Glycine* sp., *Lablab niger* Medik., *L. purpureus* (L.) Sweet, *Mucuna capitata* Wight & Arn., *M. deeringiana* (Bort) Merr., *Phaseolus aconitifolius* Jacq., *P. adenanthus* G. Mey., *P. aureus* Roxb., *P. calcaratus* Roxb., *P. coccineus* L., *P. lathyroides* L., *P. lunatus*, *P. radiatus* L., *P. sublobatus* Roxb., *P. vulgaris*, *Psophocarpus tetragonolobus* (L.) DC., *Pueraria* sp., *Strophostyles helvola* (L.) Elliott, *Vicia faba* L., *Vigna antillana* (Urb.) Fawc. & Rendle, *V. catjang* (Burm.f.) Walp., *V. cylindrica* (L.) Skeels, *V. luteola* (Jacq.) Benth., *V. marina* (Burm.) Merr., *V. mungo* (L.) Hepper, *V. repens* (L.) Kuntze, *V. sesquipedalis* (L.) Fruwirth, *V. sinensis* (L.) Savi ex Hausskn., *V. unguiculata* (L.) Walp., and further species in other genera of Fabaceae. It is widespread in warmer regions, including Afghanistan, Angola, Argentina, Australia, Azerbaijan, Bangladesh, Barbados, Bolivia, Brazil, Brunei, Cambodia, Canada, China, Colombia, Cuba, Dominican Republic, Egypt, EI Salvador, Ethiopia, Fiji, Ghana, Grenada, Guatemala, Guyana, Haiti, Honduras, Hong Kong, India, Indonesia, Iran, Iraq, Italy, Jamaica, Japan, Korea, Liberia, Malawi, Malaysia, Mauritius, Mexico, Mozambique, Myanmar, Nepal, New Caledonia, Niger, Nigeria, Pakistan, Panama, Papua New Guinea, Peru, Philippines, Puerto Rico, Russia, Rwanda, Saint Lucia, Saint Vincent and the Grenadines, Samoa, Saudi Arabia, Senegal, Sierra Leone, Singapore, Solomon Islands, Somalia, South Africa, Sri Lanka, Sudan, Suriname, Taiwan, Tanzania, Thailand, Togo, Tonga, Trinidad and Tobago, Uganda, USA, Venezuela, Virgin Islands, Zambia, Zimbabwe. (Saccardo 1886; Mulder and Holliday 1975; Ellis 1976; Yen and Lim 1980; Shin and Kim 2001; Crous and Braun 2003; Farr and Rossman 2021).

##### Notes.

Except for the presence of external hyphae and mostly slightly shorter conidiophores, the present specimen from Benin is morphologically identical to *Ps. cruenta* as known by literature (Chupp 1954; Deighton 1976). This identification is confirmed by results obtained by phylogenetic analyses based on *tef1* sequence data (see Suppl. material 4). *Ps. cruenta* is a well-known pathogen causing leaf spot diseases on species of *Vigna* and allied genera. It can cause serious yield losses of up to 40% in cowpea (Sivanesan 1990). *Ps. cruenta* is cited here for the first time for Benin.

**Figure 18.**
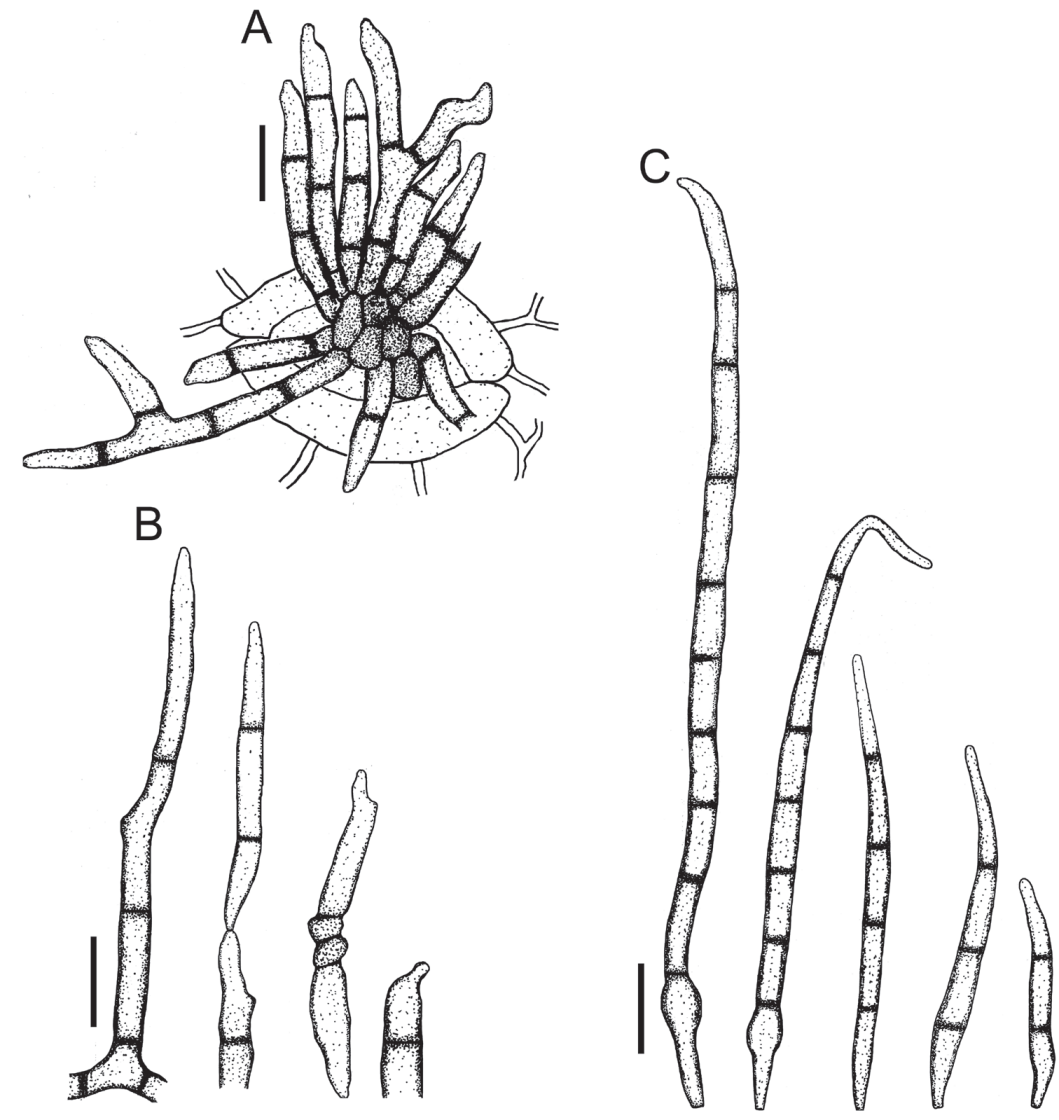
*Pseudocercospora cruenta* on *Phaseolus* sp. (YMM288) **A** fascicle of conidiophores protruding from a stomatal opening **B** solitary conidiophores **C** conidia. Scale bars: 15 μm (**A, C**); 10 μm (**B**).

#### Pseudocercospora griseola

Taxon classificationFungiMycosphaerellalesMycosphaerellaceae

(Sacc.) Crous & U.Braun, Studies in Mycology 55: 169. 2006

4371C440-2750-5821-97CB-CCA31604E323

500855

Figs 14F, G , 19

##### Basionym.

*Isariopsis griseola* Sacc., Michelia 1: 273. 1878.

For synonyms see Crous and Braun (2003), Crous et al. (2006) or MycoBank.

##### Type.

Italy. Selva, on *Phaseolus vulgaris* L. (Fabaceae), Aug 1877, Saccardo, Mycotheca Veneta 1247 (***Lectotype***: HAL, designated by Videira et al. 2017: 401, MBT378593, n.v.; ***Epitype***: CBS H-19683, designated by Videira et al. 2017: 401, MBT378594, n.v.).

For illustrations see: Saccardo (1881), Fragoso (1927), Deighton (1990), Shin and Kim (2001) or Crous et al. (2006).

##### Description.

***Leaf spots*** amphigenous, subcircular to irregularly angular, 2.5–7(–9.5) mm diam., reddish brown to dark brown or sometimes greyish brown to dark reddish brown, surrounded by a narrow darker margin. ***Caespituli*** amphigenous, mainly hypophyllous, reddish brown to olivaceous brown. ***Mycelium*** internal and external. External hyphae branched, 2.5–3 μm wide, septate, olivaceous brown to brown, smooth. ***Conidiophores*** in dense synnematous fascicles, synnemata up to 250 µm high, 20–40(–65) µm wide, emerging through stomatal openings or erumpent, or conidiophores solitary, arising from external hyphae, straight to sinuous or somewhat geniculate, 3–5(–6.5) μm wide, 1–6-septate, smooth, olivaceous brown to brown. Conidiogenous loci not thickened and not darkened, rather inconspicuous. ***Conidia*** solitary, narrowly obclavate to subacicular, straight to curved, (22–)30–78(–83) × (4.5–)5–7 μm, 2–6-septate, olivaceous brown, smooth, apex subacute to rounded, base truncate to obconically truncate, (2.5–)3–4(–4.5) µm wide, hila not thickened and not darkened.

##### Specimen examined.

Benin. Borgou: Parakou, Tankaro, c. 360 m a.s.l., 9°23'01"N, 2°30'36"E, on *Phaseolus lunatus*, 20 Sep 2019, Y. Meswaet and R. Dramani, YMM297A (M-0312675; UNIPAR).

##### Herbarium specimens examined for comparison.

*Pseudocercospora griseola*. On *Phaseolus* sp.: USA. Pennsylvania: West Chester, Gardens, Sep 1880, W. T. Harris 1363 (NY 00937289 ***Holotype*** of *Graphium laxum*). On *Phaseolus* sp.: USA. Pennsylvania: West Chester, Gardens, Sep 1880, W. T. Harris s.n (BPI 448758 ***Paratype*** of *G. laxum*). On *Phaseolus* sp.: USA. New Jersey: Newﬁeld, 27 Sep 1894, Ellis, s.n (BPI 435104 ***Paratype*** of *Cercospora columnaris* Ellis & Everh.). On *P. vulgaris*: Italy. Venetia, Selva, Aug 1877, Sacc. Mycoth. Ven. s.n (BPI 449390, ***isolectotype*** of *Isariopsis griseola*).

##### Hosts and distribution.

On *Lablab purpureus* (L.) Sweet (as *Lablab niger* Medik.), *Lathyrus odoratus* L., *Macroptilium atropurpureum* (DC.) Urb., *Phaseolus acutifolius* A. Gray, *P. coccineus* L., *P. lunatus*, *P. vulgaris*, *Vigna angularis* (Willd.) Ohwi & H. Ohashi, *V. mungo*, *V. radiata*, *V. umbellata* (Thunb.) Ohwi & H. Ohashi (as *P. pubescens* Blume), *V. unguiculata* (L.) Walp. (Fabaceae) from worldwide, including Angola, Argentina, Armenia, Australia, Austria, Bhutan, Brazil, Bulgaria, Burundi, Cameroon, Canada, China, Colombia, Costa Rica, Croatia, Cuba, Democratic Republic Congo, Dominican Republic, Ecuador, El Salvador, Ethiopia, Fiji, Georgia, Germany, Ghana, Great Britain, Greece, Guatemala, Haiti, Hungary, Jamaica, Japan, India, Indonesia, Iran, Ireland, Israel, Italy, Ivory Coast, Jamaica, Japan, Kenya, Korea, Laos, Latvia, Malawi, Madagascar, Malaysia, Mauritius, Mexico, Mozambique, Nepal, Netherlands, Netherlands Antilles, New Caledonia, New Zealand, Nicaragua, Nigeria, Norfolk Island, Panama, Papua New Guinea, Paraguay, Peru, Philippines, Poland, Portugal, Puerto Rico, Reunion (France), Romania, Russia, Rwanda, Saint Helena (British), Senegal, Sierra Leone, Singapore, Slovenia, Solomon Islands, Somalia, South Africa, Spain, Sudan, Suriname, Swaziland, Switzerland, Taiwan, Tanzania, Thailand, Trinidad and Tobago, Turkey, Uganda, Ukraine, U.S.A., Vanuatu, Venezuela, Virgin Islands, Zambia, Zimbabwe (Crous and Braun 2003; Crous et al. 2006; Farr and Rossman 2021). *Ps. griseola* is reported here for the first time for Benin.

##### Notes.

Four species of *Pseudocercospora*, namely *Ps. cruenta*, *Ps. glycines* (Cooke) Deighton, *Ps. griseola* and *Ps. stizolobii* are known agents of leaf spot diseases on *Phaseolus* spp. (Farr and Rossman 2021). The present *Pseudocercospora* sp. is phylogenetically (Fig. 1) and morphologically well distinguished from *Ps. cruenta*, *Ps. glycines* and *Ps. stizolobii* (Crous et al. 2006) by forming synnematous fascicles, longer and broader conidiophores and broader conidia. The morphology of this collection from Benin on *P. lunatus* fits well with the description of *Ps. griseola*.

Angular leaf spot (ALS) caused by *Ps. griseola* is a serious disease of common bean (*P. vulgaris*) all around the world (Ddamulira et al. 2014). It is reported for about 80 countries, where it can cause 45% to 80% losses of yield under conditions favourable for the fungus (Guzmán et al. 1999). The disease is also a major problem for bean production (50–60% of yield losses) in Africa, mainly in the Great Lakes Regions (Kenya, Uganda, Tanzania and Rwanda) where bean growing is popular (Golato and Meossi 1972; Wortmann et al. 1998; Aggarwal et al. 2004). According to Guzmán et al. (1995) and Crous et al. (2006), the species includes two major intraspecific groups, Ps. griseola f. griseola (Andean) and Ps. griseola f. mesoamericana (Middle-American) (Crous et al. 2006). Based on ITS sequence data (see Suppl. material 3), the present isolate from Benin clusters with Ps. griseola f. mesoamericana.

**Figure 19.**
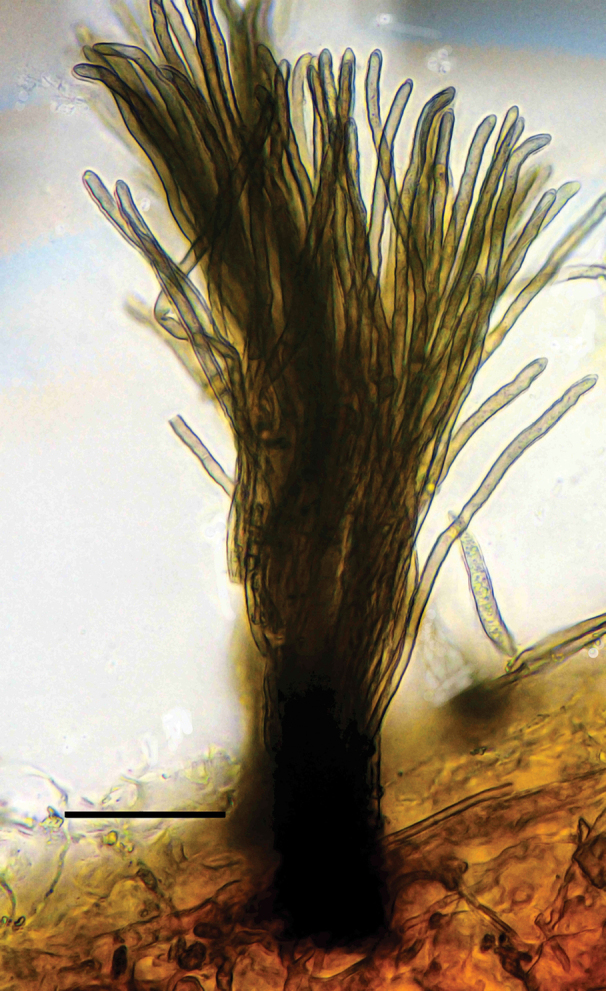
Synnematous fascicle of conidiophores of *Pseudocercospora griseola* on *Phaseolus lunatus* (YMM297A). Scale bar: 50 μm.

#### Pseudocercospora sennicola

Taxon classificationFungiMycosphaerellalesMycosphaerellaceae

Y.Meswaet, Mangelsdorff, Yorou & M.Piepenbr. sp. nov.

1DCC7938-F46A-5AB1-95DF-550CBDB7C9B7

839176

Figs 14H , 20

##### Type.

Benin. Atlantique: Cotonou, University of Abomey-Calavi, c. 9 m a.s.l., 6°24'45"N, 2°20'41"E on *Senna occidentalis* (L.) Link (Fabaceae), 23 Sep 2019, Y. Meswaet and A. Tabé, YMM12 (***Holotype***: M-0312676; ***Isotype***: UNIPAR). ***Ex holotype sequences*.**MW834444 (SSU), MW834432 (LSU), MW850550 (ITS).

##### Etymology.

The epithet *sennicola* refers to the host genus *Senna* and -*cola* (lat. *colere* = to dwell).

##### Diagnosis.

*Pseudocercospora sennicola* differs from other *Pseudocercospora* spp. known on *Senna* spp. by causing often inconspicuous spots and the combination of branched and relatively long conidiophores [16.5–)20.5–92(–98) µm] and relatively short and wide conidia [(16–)22–54.5(–65) × 3–4.5(–5) μm] that are often constricted at the septa (Table 6).

**Table 6.** Comparison of *Pseudocercospora sennicola* on *Senna occidentalis* (YMM12) with *Pseudocercospora* species known from *Senna* spp. based on literature ^a–g^.

*Cercospora* species	*Leaf spots*, colour, size	Stromata	Conidiophore size (in μm), branching, septa	Conidium sizes (in μm), septa
*Pseudocercospora sennicola* (YMM12)	Often lacking or indistinct	Lacking to slightly-developed	(16.5–)20.5–92(–98) × (3–)3.5–4.5, branched, 2–6(–8)- septate	(16–)22–54.5(–65) × 3–4.5(–5) μm, 2–6 septa, slightly constricted at the septa
*Ps. angustata* ^ab^	Brownish to dingy grey, 0.5–3 mm.	Small	10–50 × 2–3.5, unbranched, rarely septate	15–75 × 2–4 µm, 3–7 septa
*Ps. cassiae-alatae* ^c^	Present	Small	3–45 × 2.5–3.5, 0–6-septate	15–90 × 1.5–2 µm, 1–10 septa
*Ps. cassiae-fistulae* ^d^	Greyish brown to dark, 0.5–2 mm.	Well-developed	10–30 × 2.5–5, unbranched, 0–2-septate	25–65 × 3–4 µm, 2–8 distinct septa
*Ps. cassiae-occidentalis* ^c^	Indistinct	Absent	60–130 × 4–5, unbranched, 2–6-septate	62–100 × 3.5–4.8, 3–6 septa
*Ps. cassiae-siameae* ^be^	Present	Present	15.3–27.2 × 3.4–4.2, 0–1-septate	28.9–93.5 × 3.4–4.2, 2–8 septa
*Ps. nigricans* ^bdf^	Yellowish discoloration to greyish brown, 2–3 mm wide	Small	15–125 × 3–5, branched, 1–3-septate	20–80 × 3–5, 1–10 septa
*Ps. sennae-multijugae* ^g^	Grey brown, 2–18 mm in diam.	Well-developed (5–67 µm diam.)	11–81 × 3–4, unbranched, 0–2-septate	75–170 × 2–3.5, 2–7 septa
*Ps. singaporensis* ^c^	Yellowish to brownish grey, 0.5–4 mm in diam.	Absent	31–77 × 4.5–5.5, branched, 0–2(–4)-septate.	30–67 × 3.5, 3 (rarely 1 or 4) septa
*Ps. taichungensis* ^d^	Greyish brown, 1–5 mm wide	Well-developed	10–25 × 1–4.3, unbranched, 0–2-septate	20–55–100 × 1.5–3, 1–6 indistinct septa

**^a^**Lenné (1990), ^b^Deighton (1976), ^c^Yen and Lim (1980), ^d^Hsieh and Goh 1990, ^e^Chiddarwar (1959), ^f^ Cooke and Massee (1883-1884), ^g^Silva et al. (2016)

##### Description.

***Leaf spots*** lacking or indistinct to pale brown discolorations, amphigenous, subcircular to irregularly angular, (2–)4.5–10.5 mm diam., occasionally surrounded by a darker margin. ***Caespituli*** amphigenous, loose, olivaceous brown. ***Mycelium*** internal and external. External hyphae branched, 2.5–3.5 μm wide, septate, olivaceous brown to brown, smooth. ***Stromata*** lacking to slightly developed, in substomatal cavities or partly embedded in the mesophyll, 10–20 μm diam., brown to dark brown. ***Conidiophores*** in small, loose fascicles of up to approx. 10 conidiophores, arising from internal hyphae or hyphal aggregations, breaking through the adaxial epidermis of the leaves or penetrating through stomatal openings, or solitary, arising from external hyphae, erect to decumbent, flexuous, simple or occasionally branched, subcylindrical to somewhat clavate, geniculate-sinuous, slightly narrower towards the tips, (16.5–)20.5–92(–98) × (3–)3.5–4.5 μm, 2–6(–8)-septate, smooth, olivaceous brown to slightly dark brown, paler towards the tips. ***Conidiogenous cells*** terminal or lateral, medium brown, smooth, proliferating sympodially, with slightly tapering to flat-tipped apical loci; loci 1.5–2.5 μm wide, not thickened and not darkened. ***Conidia*** solitary, narrowly obclavate to subacicular, straight to curved, (16–)22–54.5(–65) × 3–4.5(–5) μm, 2–6-septate, often constricted at the septa, olivaceous brown, smooth, apex subacute, base truncate to obconically truncate, 1.5–2.5 μm wide, hila not thickened and not darkened.

##### Additional specimen examined.

Benin. Atlantique: Cotonou, University of Abomey-Calavi, c. 9 m a.s.l., 6°24'45"N, 2°20'41"E, on *Senna occidentalis*, 26 Sep 2019, Y. Meswaet and A. Tabé, YMM12B (***Paratypes***: M-0312677; UNIPAR).

##### Herbarium specimens examined for comparison.

On *Senna occidentalis* (as *Cassia occidentalis* L.): USA. South Carolina: Aiken, 1876, Ravenel H. W. s.n. (BPI 439584, ***Holotype*** of Cladosporium personatum var. cassiae Thüm.).

##### Host and distribution.

On *Senna occidentalis* (Fabaceae) in Benin.

##### Notes.

Currently, eleven *Pseudocercospora* species are known on *Senna* spp. (Fabaceae), namely *Ps. angustata* (Chupp & Solheim) Deighton on *Senna hirsuta* (L.) H.S. Irwin & Barneby, *Ps. cassiae-alatae* (J.M. Yen & Lim) J.M. Yen on *S. alata* (L.) Roxb., *Ps. cassiae-fistulae* Goh & W.H. Hsieh on *Cassia fistula* L. and *S. rizzinii* H.S. Irwin & Barneby, *Ps. cassiae-occidentalis* (J.M. Yen) J.M. Yen on *S. occidentalis*, *Ps. cassiae-siameae* (Chidd.) Deighton on *S. siamea* (Lam.) H.S. Irwin & Barneby, *Ps. nigricans* (Cooke) Deighton on *Senna* spp., *Ps. sennae-multijugae* on *S. multijuga* (Rich.) H.S. Irwin & Barneby, *Ps. sennae-rugosae* A. Hern. Gut., Z.M. Chaves & Dianese on *S. rugosa* (G. Don) H.S. Irwin & Barneby, *Ps. singaporensis* (J.M. Yen) J.M. Yen on *S. occidentalis* (L.) Link, *Ps. taichungensis* Goh & W.H. Hsieh on *S. atomaria* (L.) H.S. Irwin & Barneby (Hernández-Gutiérrez et al. 2015; Farr and Rossman 2021). Among these eleven species of *Pseudocercospora*, only *Ps. nigricans* and *Ps. singaporensis* have some similarity with the species described here (Table 6). *Ps. sennicola*, however, differs from *Ps. nigricans* in causing often indistinct leaf spots, shorter conidiophores [(16.5–)20.5–92(–98) μm versus 15–125 µm in *Ps. nigricans*] with 6(–8) septa and shorter conidia [(16–)22–54.5(–65) versus 20–80 μm in *Ps. nigricans*] (Hsieh and Goh 1990). *Ps. sennicola* differs from *Ps. singaporensis* by causing often indistinct leaf spots, slightly developed stromata, longer conidiophores [(16.5–)20.5–92(–98) μm versus 31–77 µm in *Ps. singaporensis*] and shorter conidia [(16–)22–54.5(–65) versus 30–67 μm in *Ps. singaporensis*] (Yen and Lim 1980). Moreover, the specimen from Benin has conidia with more septa (2–6 versus strictly 3-septate in *Ps. singaporensis*) and conidial walls constricted at the septa.

In the multi-gene tree (Fig. 1), *Ps. sennicola* is located in a polytomy at the end of a long branch reflecting a long genetic distance to other species included in the analysis. Morphologically, *Ps. sennicola* is distinct from all *Pseudocercospora* species known on species of Fabaceae from Benin by longer conidiophores [(16.5–)20.5–92(–98) μm] and smaller conidia [(16–)22–54.5(–65) μm)].

Based on a MegaBLAST search using the ITS sequence data, the closest matches in NCBI’s GenBank nucleotide database were *Pseudocercospora fuligena* on *Lycopersicon* sp. (Solanaceae) from Thailand (GenBank GU214675; Identities 674/687, i.e., 98%), *Pseudocercospora chengtuensis* on *Lycium chinense* (Solanaceae) from South Korea (GenBank GU214672; Identities 674/687, i.e., 98%) and *Pseudocercospora atromarginalis* on *Solanum nigrum* L. (Solanaceae) from South Korea (GenBank GU214671; Identities 673/687, i.e., 97%). Based on the result of our comparative study, we consider the present *Pseudocercospora* species on *Senna occidentalis* from Benin to represent a distinct species, which is described here. However, as sequence data are only available for *Ps. sennae-multijugae*, more molecular sequence data are needed to clarify the species delimitations among these twelve *Ps.* species on *Senna* spp.

**Figure 20.**
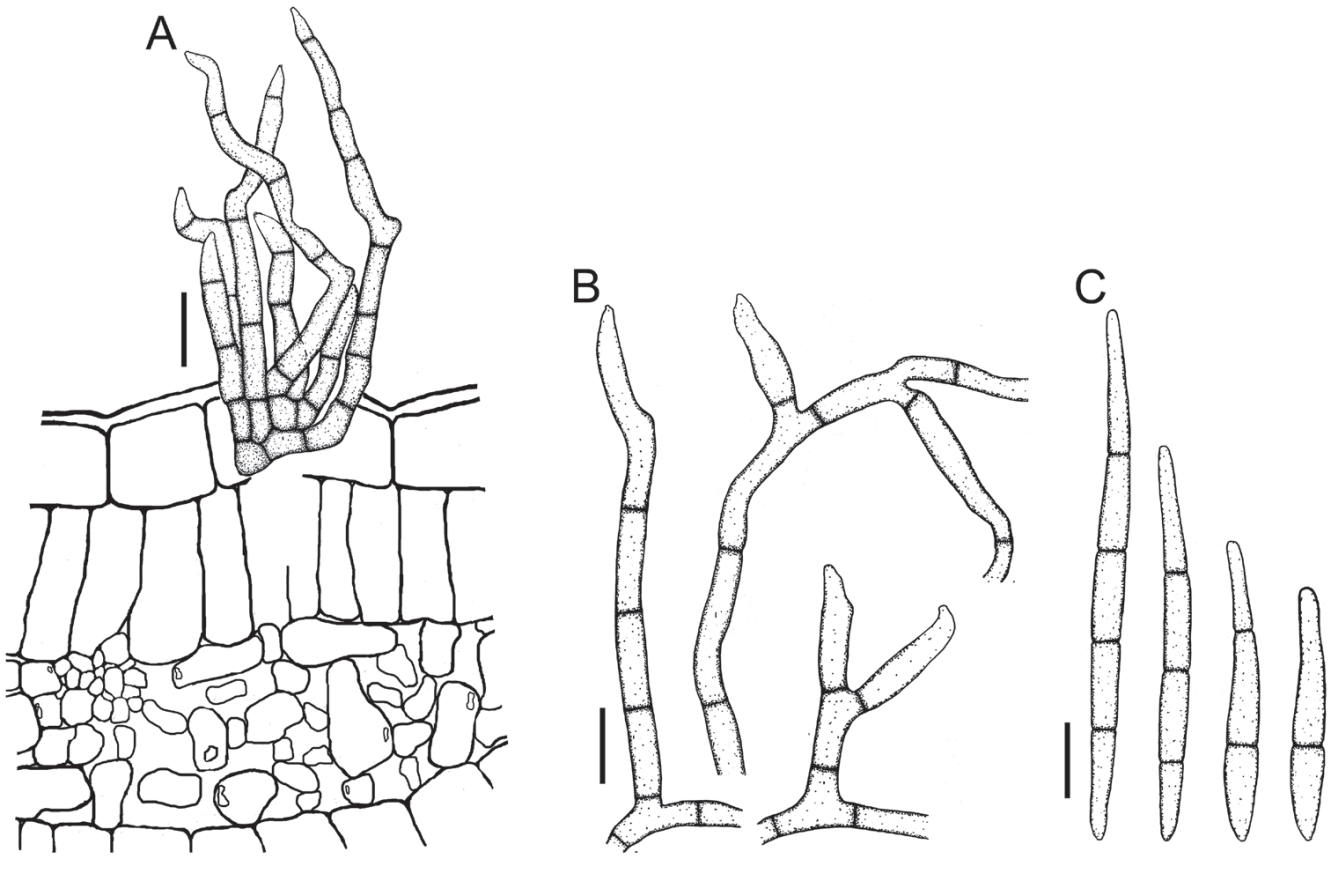
*Pseudocercospora sennicola* on *Senna occidentalis* (YMM12) **A** fascicle of conidiophores growing out of a small stroma embedded in the epidermis **B** solitary conidiophores arising from external hyphae **C** conidia. Scale bars: 15 μm (**A**); 10 μm (**B, C**).

#### Pseudocercospora tabei

Taxon classificationFungiMycosphaerellalesMycosphaerellaceae

Y.Meswaet, Mangelsdorff, Yorou & M.Piepenbr. sp. nov.

C528D5E1-DBFD-5FEA-A628-F245DBA17C68

839177

Figs 14I , 21

##### Type.

Benin. Borgou: Parakou, c. 360 m a.s.l., 9°20'07"N, 2°38'50"E, on *Vigna unguiculata* (L.) Walp. (Fabaceae), 2 Sep 2019, Y. Meswaet and A. Tabé, YMM220 (***Holotype***: M-0312678; ***Isotype***: UNIPAR). ***Ex holotype sequences*.**MW834450 (SSU), MW834434 (LSU), MW834439 (ITS), MW848617 (*tef1*).

##### Etymology.

The epithet *tabei* refers to the person who collected the type specimen, Affoussatou Tabé, mycologist at the University of Parakou, Benin.

##### Diagnosis.

*Pseudocercospora tabei* differs from other *Pseudocercospora* spp. known on *Vigna* spp. by external hyphae, well-developed stromata, as well as the sizes of conidiophores [(20.5–)24–82(–84.5) × 3–4(–4.5) μm] and conidia [(20.5–)24–82(–84.5) × 3–4(–4.5) μm] (Table 7).

**Table 7.** Comparison of *Pseudocercospora tabei* YMM220 on *Vigna unguiculata* with *Pseudocercospora* species known from *Vigna* spp. based on literature ^a–d^.

*Pseudocercospora* species	Stromata	Conidiophore size (in μm), branching, septa	Conidia size (in μm), septa
*Pseudocercospora tabei* (YMM220)	Small or well-developed up to 45 μm diam.	(11.5–)14.5–40(–44.5) × (3–)3.5–4(–4.5), branched, 0–4-septate	(20.5–)24–82(–84.5) × 3–4(4.5), 2–6(–8) septa
*Ps. cruenta* ^a^	Up to 30 μm diam.	10–75 × 3–5, branched, 0–3-septate	25–120 × 2–5, 3–14 septa
*Ps. mungo* ^b^	Up to 30 μm diam.	Up to 90(–130) × 4.5–7.5, branched, 1–3-septate	25–84 × 4.5–7.5, 3–8 septa
*Ps. phaseolicola* ^a^	Absent	3–25 × 1.5–3	20–90 × 1.5–2, indistinctly septate
*Ps. shihmenensis* ^a^	Absent	35–55 × 4–5, branched, 1–4-septate	20–52 × 4–5, 3–8 septa
*Ps. vexillatae* ^ac^	Presen t	10–17 × 4–5, unbranched, continuous or rarely 1-septate	40–100 × 2.5–4, 3–8 septa
*Ps. vignae-reticulatae* ^b^	Small	40–250 × 3.5–5.5, branched	30–95 × 4 –6.5, 1–12 septa
*Ps. vignicola* ^c^	Well-developed	22–75 × 3–5, branched, 0–1-septate	30–60 × 2.5–3, 3–6 septa
*Ps. vignigena* ^d^	Small	22–75 × 3–5, unbranched, 1–3-septate	33–60 × 4–5.5(–6), 3–6 septa

^a^Hsieh and Goh (1990), ^b^Deighton (1976), ^c^Braun et al. (1999), ^d^Yen et al. (1982)

##### Description.

***Leaf spots*** amphigenous, subcircular to irregularly angular, 2.5–7.5 mm diam., occasionally limited by veins, yellowish brown to pale brown, reddish brown to dark brown when old, more evident on the adaxial surface of the leaves, margin indefinite. ***Caespituli*** amphigenous, brown. ***Mycelium*** internal and external. External hyphae branched, 2–2.5(–3.5) μm wide, septate, olivaceous brown to brown, smooth. ***Stromata*** lacking or formed by few aggregated swollen hyphal cells to well-developed, up to approx. 45 μm diam., immersed in the mesophyll or in substomatal chambers, globular to irregular, brown to mostly dark brown. ***Conidiophores*** in small, loose to moderately dense fascicles arising from stromata, breaking through the adaxial epidermis of the leaves or penetrating through stomatal openings, or solitary, arising from external hyphae, straight to sinuous or somewhat geniculate, simple or rarely branched, (11.5–)14.5–40(–44.5) × (3–)3.5–4(–4.5) μm, 0–4-septate, smooth, olivaceous brown to brown, paler towards the tips, sometimes a conidiophore is reduced to a single conidiogenous cell. ***Conidiogenous cells*** terminal or lateral, rarely up to 20 μm long, pale or olivaceous brown, smooth, proliferating sympodially; loci 2–3.5 μm wide, not thickened and not darkened. ***Conidia*** solitary, narrowly cylindrical to obclavate-cylindrical, straight to slightly curved, (20.5–)24–82(–84.5) × 3–4(–4.5) μm, conspicuously 2–6(–8)-septate, olivaceous brown, smooth, apex subacute to rounded and narrower than the rest of the conidium, base truncate, (2–)2.5–3.5 µm wide, hila not thickened and not darkened.

##### Additional specimens examined.

Benin. Borgou: Parakou, c. 354 m a.s.l., 9°20'02"N, 2°38'48"E, on *Vigna unguiculata*, 27 Aug 2019, Y. Meswaet and A. Tabé, YMM232A (***Paratypes***: M-0312679; UNIPAR). Benin. Borgou: Parakou, c. 391 m a.s.l., 9°22'56"N, 2°37'33"E, same host, 29 Aug 2019, Y. Meswaet and A. Tabé, YMM232B (M-0312680).

##### Herbarium specimens examined for comparison.

*Pseudocercospora cruenta*. On *Vigna unguiculata*: USA. Mississippi: Starkville, Sep 1888, Tracy S. M. s.n. (BPI 435817 ***Paratype*** of *Cercospora dolichi*). On *Phaseolus* sp.: USA. South Carolina: Aiken, Ravenel H. W. s.n (BPI 439619, type of *C. phaseolorum*). *Pseudocercospora stizolobii*. On *Mucuna* sp.: Philippines. Los Baños, 6 Apr 1913, Raimundo M. B. 892 (BPI 441666, ***Holotype*** of *C. stizolobii*).

##### Host and distribution.

On *Vigna unguiculata* (Fabaceae) in Benin.

##### Notes.

On species of *Vigna*, eight species of *Pseudocercospora*, namely *Ps. cruenta*, *Ps. mungo* Deighton, *Ps. phaseolicola* Goh & W.H. Hsieh, *Ps. shihmenensis* (J.M. Yen) J.M. Yen, *Ps. vexillatae* (J.M. Yen) U.Braun, *Ps. vignae-reticulatae* Deighton, *Ps. vignicola* (J.M. Yen, A.K. Kar & B.K. Das) U.Braun and *Ps. vignigena* J.M. Yen, A.K. Kar & B.K. Das are known (Farr and Rossman 2021). Among these species, *Ps. mungo* described on *Vigna radiata*, *V. mungo* from Tanzania (East Africa) (Deighton 1976) and *Ps. phaseolicola* on *Vigna radiata* from China and Taiwan (Hsieh and Goh 1990) are morphologically similar to the present *Pseudocercospora* specimen from Benin (Table 7). Based on the original description by Deighton (1976), *Ps. mungo*, however, differs from the present species in causing leaf spots that form only indefinite chlorotic areas on the adaxial surface, hypophyllous caespituli, lack of external hyphae and above all, by longer and wider conidiophores [up to 90(–130) × 4.5–7.5 µm)] and wider conidia (4.5–7.5 µm) (Deighton 1976). *Ps. tabei* causes yellowish brown to pale brown leaf spots, that are reddish brown to dark brown, when old, forms amphigenous caespituli, often produces well developed stromata, external hyphae and above all, shorter and narrower conidiophores [(11.5–)14.5–40(–44.5) × (3–)3.5–4(–4.5) µm] and narrower conidia (3–4 μm). *Ps. phaseolicola* differs by producing hypophyllous caespituli, no stromata, non-fasciculate, olivaceous, shorter and narrower conidiophores [3–25 × 1.5–3 µm versus (11.5–)14.5–40(–44.5) × (3–)3.5–4(–4.5) µm in *Ps. tabei*] and narrower conidia [1.5–2 µm versus 3–4 µm in *Ps. tabei*] (Hsieh and Goh 1990).

**Figure 21.**
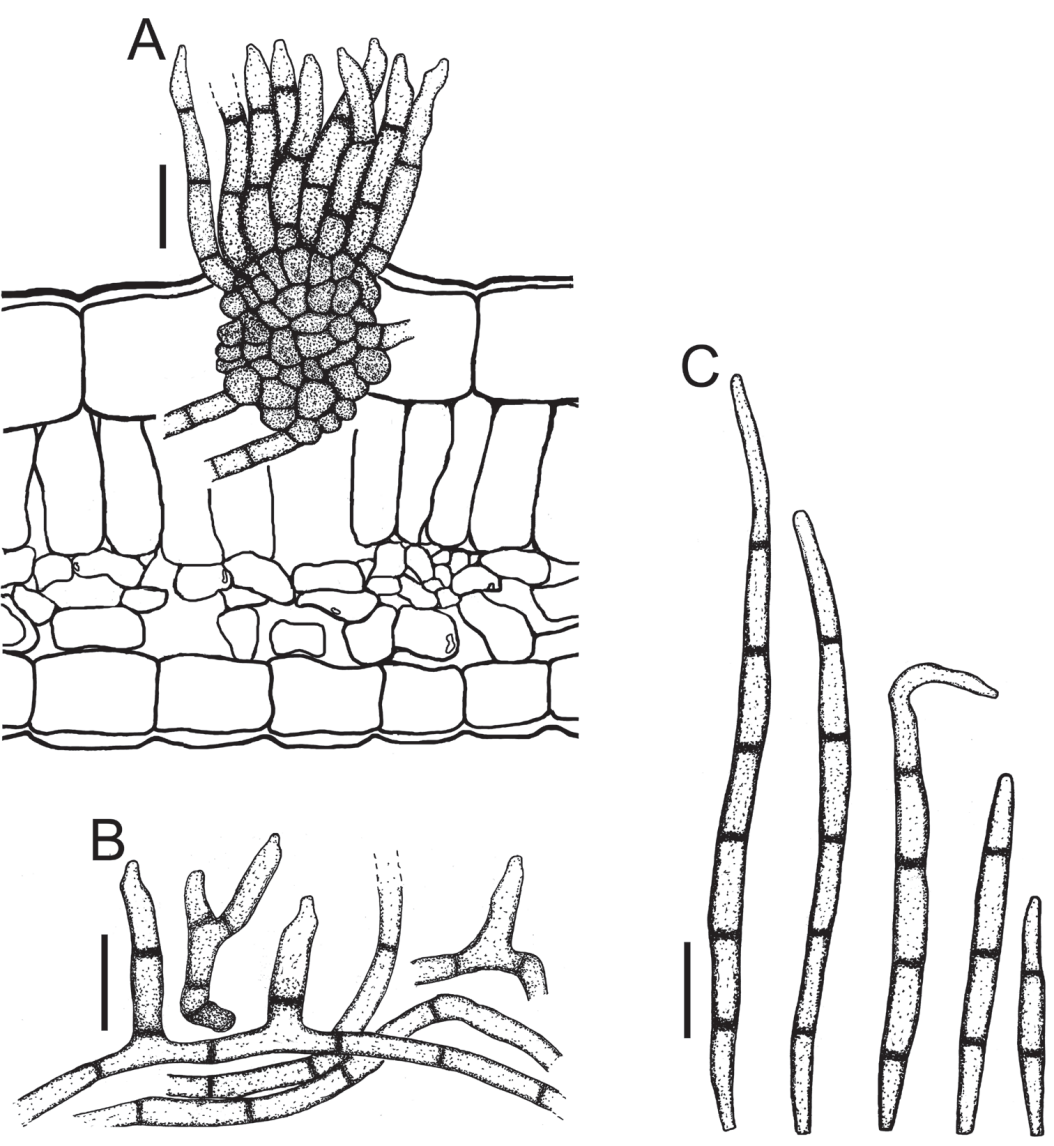
*Pseudocercospora tabei* on *Vigna unguiculata* (YMM220) **A** immersed stroma with conidiophores **B** solitary conidiophores arising from external hyphae **C** conidia. Scale bars: 15 μm (**A**); 12 μm (**B**); 10 μm **(C**).

In the multi-gene phylogeny (Fig. 1), *Ps. tabei* forms part of a polytomy with a large genetic distance (branch length) in relation to other sequences considered in the analysis. In the *tef1* phylogeny, *Ps. tabei* clustered together with the isolates of *Ps. cruenta* on *Vigna* and *Phaseolus* form Benin (see Suppl. material 4). However, morphologically, the present species is clearly distinct from specimens of *Ps. cruenta* by having darker and shorter conidiophores and above all, shorter conidia [(20.5–)24–82(–84.5) μm] (Table 7). It is not possible to distinguish *Ps. tabei* from other numerous *Pseudocercospora* spp. by the phylogenetic analyses based on ITS sequences.

Based on a MegaBLAST search in the NCBI GenBank nucleotide database using the *tef1* sequence, the closest matches were *Ps. cruenta* on *Phaseolus vulgaris* (Fabaceae) from Taiwan (GenBank GU384405; Identities 283 / 312, i.e., 90%), *Pseudocercospora* sp. A on *P. vulgaris* (Fabaceae) from Iran MB-2015(GenBank KM452885; Identities 263 / 292, i.e., 90%) and *Ps. madagascariensis* on *Eucalyptus camaldulensis* (Myrtaceae) from Madagascar (GenBank KF253265; Identities 276 / 314, i.e., 88%).

### Key 1: Key to species of *Cercospora* on Fabaceae known for Benin

**Table:** 

1	Stromata well-developed, i.e., usually broader than 40 μm diam	**2**
–	Stromata lacking or small, i.e., usually less than 20 μm diam	**3**
2	Conidiophores branched, with polyblastic conidiogenous cells, conidia mostly 26–160 × 4–5 μm. On *Vigna*	***C. rhynchophora***
–	Conidiophores unbranched, usually with monoblastic conidiogenous cells, conidia mostly 27–70 × 2–3 μm. On *Lablab*	**C. cf. fagopyri**
3	Stromata totally lacking, hyphae mainly internal, conidiophores branched, mostly 18–178 × 4–5 μm, conidia mostly 19–88 × 3.5–4.5. On *Desmodium*	***C. parakouensis***
–	Stromata often formed by few aggregated swollen hyphal cells with similar morphology	**4**
4	Conidiophores up to 400 μm long. On *Vigna*.	**5**
–	Conidiophores usually not longer than 150 μm	**6**
5	Leaf spots inconspicuous or absent, caespituli mostly epiphyllous, conidia mostly 38–188 μm long	***C. tentaculifera***
–	Leaf spots conspicuous, brown to later with necrotic centre, caespituli mostly hypophyllous, conidia mostly 26–100 μm long	***C. vignae-subterraneae***
6	Conidia up to 330 μm long. On *Calopogonium*, *Vigna*	**C. aff. canescens**
–	Conidia mostly 20–160 μm long	**7**
7	Only internal hyphae	**8**
–	Internal and external hyphae	**9**
8	Internal hyphae often distinct and developed, conidiophores in loose to moderately large and dense fascicles of up to approx. 16. On *Crotalaria*	***C. beninensis***
–	Internal hyphae often indistinct, conidiophores in small and loose fascicles of up to approx. 6 conidiophores, conidiophores mostly attenuated towards the tips. On *Vigna*	***C. phaseoli-lunati***
9	Conidiophores unbranched, in small, loose or moderately large and dense fascicles of up to approx. 22. On *Vigna*	**C. cf. canscorina**
–	Conidiophores branched	**10**
10	Leaf spots almost lacking or brown discolorations, often uniform in colour and width, conidia hyaline. On *Zornia*	***C. zorniicola***
–	Leaf spots often developed, reddish brown, later dark brown by abundant caespituli, conidia often sub-hyaline. On *Phaseolus*	***Cercospora* sp. YMM297B**

### Key 2: Key to the species of *Pseudocercospora* on Fabaceae known for Benin

**Table:** 

1	Conidiophores in synnematous fascicles, synnemata up to 250 µm high, mostly 20–40 µm wide. On *Phaseolus*	***Ps. griseola***
–	Conidiophores solitary, fasciculate or in sporodochia	**2**
2	Stromata well-developed	**3**
–	Stromata lacking or very small	**4**
3	Leaf spots often lacking or indistinct, conidiophores often narrower towards the tips, mostly 20–92 μm long, conidia, mostly 22–55 μm long, constricted at the septa. On *Senna*	***Ps. sennicola***
–	Leaf spots evident, conidiophores, mostly 14–40 μm long, conidia mostly 24–82 μm long. On *Vigna*	***Ps. tabei***
4	Caespituli amphigenous, conidiophores mostly 15–54 μm long, conidia mostly 42–132 μm long. On *Phaseolus*, *Vigna*	***Ps. cruenta***
–	Caespituli mainly epiphyllous, conidiophores mostly 13–44 μm long, conidia mostly 38–110 μm long. On *Centrosema*	***Ps. bradburyae***

## Discussion

The present study aims to increase the knowledge on the diversity of cercosporoid fungi in tropical Africa. Therefore, cercosporoid fungi collected on fifteen species of plants belonging to ten genera of Fabaceae found in Benin, West Africa, were characterised concerning their morphology, host species and DNA sequence data (18S rDNA, 28S rDNA, ITS and *tef1*). The specimens of cercosporoid species collected in Benin are attributed to groups corresponding to *Cercospora*, *Pseudocercospora* and a heterogeneous group around *Passalora*. The four-gene phylogenetic tree yielded results consistent with the current knowledge of generic relationships as presented in previous studies (Crous et al. 2013; Groenewald et al. 2013; Nakashima et al. 2016). Species of *Cercospora* and *Pseudocercospora* form morphologically distinct groups that receive moderate support in the phylogenetic analysis (Fig. 1). In the *Cercospora* and *Pseudocercospora* clades, the lengths of branches of most new species (*C. beninensis*, *C. rhynchophora*, *C. vignae-subterraneae*, *C. zorniicola*, *Ps. sennicola* and *Ps. tabei*) are quite long (Fig. 1). This indicates a relatively large genetic and evolutionary distance from neighbouring species included in the analysis. The partial gene sequences of the protein-coding region *tef1* and the combined analysis of four loci provided better results than single gene analyses of ITS and LSU rDNA for the differentiation of species of *Cercospora* and *Pseudocercospora*. Consequently, these molecular sequence data only allow to measure phylogenetic distances between the species. A similar situation has been found for *Cercospora* spp. by Bakhshi et al. (2015, 2018) and for *Pseudocercospora* spp. by Crous et al. (2013a) and Silva et al. (2016).

Fortunately, most species included in this study differ from each other by their morphology and host range. For example, *Cercospora tentaculifera* (YMM75) on *Vigna unguiculata* causes inconspicuous leaf spots and produces adaxial caespituli with large conidiophores (up to 435 μm) that are constricted at the septa (Figs 2H, 11). Thereby, this species is easily distinguishable from other *Cercospora* spp. known on species of *Vigna* and *Phaseolus*. *C. zorniicola* (YMM299) on *Zornia glochidiata* produces external hyphae and conidiophores that are unbranched and uniform in colour and width with usually monoblastic conidiogenous cells (Fig. 13). This is the first species of *Cercospora* known for the host genus *Zornia*.

For the morphological identification of all species included in this study, we examined about 50 type specimens and other specimens loaned from BPI, ILL and NY. As result of these analyses, dichotomous keys to the species of *Cercospora* and *Pseudocercospora* infecting members of Fabaceae known for Benin are presented (see below). The following morphological characteristics are helpful to separate species of *Cercospora* and *Pseudocercospora*: characteristics of leaf spots (distinctiveness, colour, size, form) and sporulation (distinctiveness, position on the leaf), the stroma (size, density), the external hyphae (present/absent), conidiophores (form, size, branching, number and position of conidiogenous loci, form of conidiogenous cells), and conidia (form, size range) (comp. Deighton 1976; Crous and Braun 2003; Crous et al. 2013; Groenewald et al. 2013; Videira et al. 2017).

In order to obtain DNA sequence data, up to now, only cercosporoid fungi available as cultures have been used (Groenewald et al. 2013). Due to the fact that most cercosporoid fungi are not available as cultures, molecular sequences are available only for a small fraction of the species diversity of cercosporoid fungi known by morphological characteristics. It is often difficult to cultivate cercosporoid fungi, as this requires living fungal cells and a sterile environment to avoid contamination. As it was not possible to cultivate cercosporoid fungi in Benin, a technique for DNA isolation from dry specimens has been developed and successfully applied in the context of the present study for cercosporoid fungi for the first time. This direct DNA extraction method opens interesting possibilities to obtain DNA data of cercosporoid and other fungal plant pathogens especially in tropical countries.

The present study is the first effort towards generating molecular and morphological data for cercosporoid fungi in Benin, West Africa. We found 18 taxa, representing only a small fraction of the yet unknown species diversity of cercosporoid fungi (Piepenbring et al. 2020; Farr and Rossman 2021). Eight taxa found in this study are proposed as species new to science. Ten known species have been identified, including taxa important for agriculture such as *Pseudocercospora cruenta* and *Ps. griseola* on *Phaseolus lunatus* as well as *Nothopassalora personata* and *Passalora arachidicola* on *Arachis hypogaea*. Eight species are reported for Benin for the first time, with three of them namely, Cercospora cf. canscorina, C. cf. fagopyri and *C. phaseoli-lunati*, being new for West Africa.

New scientific data, such as species new to science, new records of hosts and for geographic areas, will help plant pathologists to develop efficient and sustainable disease management programs to control these fungal diseases and quarantine officials to take decisions based on scientific evidence. The plethora of novel and newly reported taxa collected on Fabaceae in Benin confirms that mycologists and phytopathologists in Africa have so far not given much attention to the species diversity of fungi occurring on plants, including species of economic relevance, such as those belonging to Fabaceae. Benin and other tropical African countries are likely to harbour highly diverse mycobiomes including cercosporoid fungi that still await discovery (Piepenbring et al. 2020). It is important to investigate them, because these unknown plant pathogens are or may become relevant as agents of emerging diseases that may spread and threaten cultivated plants worldwide. We hope that this study motivates further mycologists to study cercosporoid fungi in Benin, as well as in other countries of tropical Africa, and help to get a better understanding of cercosporoid fungal diversity worldwide.

## Conclusions

The present study is a first step for the investigation of the diversity of cercosporoid fungi by an integrative approach including morphological, phylogenetic and ecological information. Taxonomic studies in this work generated eight newly described species, eight new records and the confirmation of two species of cercosporoid fungi that were previously reported from Benin. Previously, 12 cercosporoid fungi were known for Benin. The present work expands this number by adding 16 species of *Cercospora* and *Pseudocercospora* to this list, with a total of 28 species. These records together with herbarium specimens and molecular sequence data form a baseline for further studies in the field of systematics, ecology and phytopathology referring to cercosporoid fungi. This information will help plant pathologists to develop effective disease management programs and evidence-based quarantine regulations. The results obtained for a single family (Fabaceae) in easily accessible vegetation close to settlements suggest that many more taxa of cercosporoid fungi remain to be discovered on plants belonging to other family of plants in diverse habitats. In the future, more attention should be directed towards collecting cercosporoid and other pathogenic fungi from Benin as well as other parts of tropical Africa.

## Supplementary Material

XML Treatment for Cercospora beninensis

XML Treatment for Cercospora aff. canescens

XML Treatment for Cercospora cf. canscorina

XML Treatment for Cercospora cf. fagopyri

XML Treatment for Cercospora parakouensis

XML Treatment for Cercospora phaseoli-lunati

XML Treatment for Cercospora rhynchophora

XML Treatment for Cercospora

XML Treatment for Cercospora tentaculifera

XML Treatment for Cercospora vignae-subterraneae

XML Treatment for Cercospora zorniicola

XML Treatment for Nothopassalora personata

XML Treatment for Passalora arachidicola

XML Treatment for Pseudocercospora bradburyae

XML Treatment for Pseudocercospora cruenta

XML Treatment for Pseudocercospora griseola

XML Treatment for Pseudocercospora sennicola

XML Treatment for Pseudocercospora tabei

## Supplementary materials

Supplementary material 1 Checklist for cercosporoid fungi in West Africa Data type Checklist Explanation note This information is based on the checklist published by Piepenbring et al. (2020) and updated with new results from the present publication and some further publications. https://binary.pensoft.net/file/556620This dataset is made available under the Open Database License (http://opendatacommons.org/licenses/odbl/1.0/). The Open Database License (ODbL) is a license agreement intended to allow users to freely share, modify, and use this Dataset while maintaining this same freedom for others, provided that the original source and author(s) are credited.Yalemwork Meswaet, Ralph Mangelsdorff, Nourou S. Yorou, Meike Piepenbring

Supplementary material 2 References for the checklist for cercosporoid fungi in West Africa Data type text Explanation note References for the checklist for cercosporoid fungi in West Africa (Suppl. material 1). https://binary.pensoft.net/file/556621This dataset is made available under the Open Database License (http://opendatacommons.org/licenses/odbl/1.0/). The Open Database License (ODbL) is a license agreement intended to allow users to freely share, modify, and use this Dataset while maintaining this same freedom for others, provided that the original source and author(s) are credited.Yalemwork Meswaet, Ralph Mangelsdorff, Nourou S. Yorou, Meike Piepenbring

Supplementary material 3 A Bayesian phylogenetic tree inferred from ITS rDNA sequence data of cercosporoid species Data type phylogenetic Explanation note Nodes receiving Bayesian PP ≥ 0.94 are considered as strongly supported and are indicated by thickened branches. Newly described species are denoted in bold and red text, newly reported species are indicated in blue text. https://binary.pensoft.net/file/556622This dataset is made available under the Open Database License (http://opendatacommons.org/licenses/odbl/1.0/). The Open Database License (ODbL) is a license agreement intended to allow users to freely share, modify, and use this Dataset while maintaining this same freedom for others, provided that the original source and author(s) are credited.Yalemwork Meswaet, Ralph Mangelsdorff, Nourou S. Yorou, Meike Piepenbring

Supplementary material 4 A Bayesian phylogenetic tree inferred from *tef1* DNA sequence data of cercosporoid species Data type phylogenetic Explanation note Nodes receiving Bayesian PP ≥ 0.94 are considered as strongly supported and are indicated by thickened branches. Newly described species are denoted in bold and red text, newly reported species are indicated in blue text. https://binary.pensoft.net/file/556623This dataset is made available under the Open Database License (http://opendatacommons.org/licenses/odbl/1.0/). The Open Database License (ODbL) is a license agreement intended to allow users to freely share, modify, and use this Dataset while maintaining this same freedom for others, provided that the original source and author(s) are credited.Yalemwork Meswaet, Ralph Mangelsdorff, Nourou S. Yorou, Meike Piepenbring
