# Supplementary material for: Unravelling unexplored diversity of cercosporoid fungi (Mycosphaerellaceae, Mycosphaerellales, Ascomycota) in tropical Africa
Source: MycoKeys. 2021 Jun 17;81:69–138. doi: 10.3897/mycokeys.81.67850 (PMC8225595; doi:10.3897/mycokeys.81.67850)
Supplement: Supplementary material 2 — References for the checklist for cercosporoid fungi in West Africa [file mycokeys-81-069-s002.docx]

**References to literature including records of cercosporoid and ramularioid**

**fungi in West Africa**

Blomme G, Ploetz R, Jones D, Langhe E de, Price N, Gold C, Geering A, Viljoen A, Karamura D, Pillay M, Tinzaara W, Teycheney P-Y, Lepoint P, Karamura E, Buddenhagen I (2013) A historical overview of the appearance and spread of *Musa* pests and pathogens on the African continent: highlighting the importance of clean *Musa* planting materials and quarantine measures. Annals of Applied Biology 162: 4–26.

Bouhot D (1966) Quelques champignons phytopathogènes nouveaux ou peu connus en Sénégal. Bulletin Trimestriel de la Société Mycologique de France 82: 274–300.

Chevaugeon J (1950) Maladies cryptogamiques du manioc en Côte d'Ivoire. I. - Observations préliminaires sur la nécrose des sommités. Revue de Pathologie Végétale et d'Entomologie Agricole de France 29: 3–8.

Chevaugeon J (1951b) *Cercospora personata* (B. et C.) Ellis. Cercosporiose de l'arachide. Revue de Mycologie. Supplément Colonial 16(2): 1–7.

Chevaugeon J (1952) Maladies des plantes cultivées en Moyenne-Casamance et dans le delta central nigérien. Revue de Pathologie Végétale et d'Entomologie Agricole de France 31(1): 3–51.

Chevaugeon J (1956a) Les maladies cryptogamiques du manioc en Afrique occidentale. Encyclopédie Mycologique 28: 1–205.

Chevaugeon J (1956b) Enquête phytopathologique dans le bassin du Cavally. Revue de Mycologie, Supplément Colonial 21/22: 57–86.

Chupp C (1954) A Monograph of the Fungus Genus *Cercospora*. Ithaca, New York. Published by the author.

Crous PW, Braun U (2003) *Mycosphaerella* and its anamorphs: 1. Names published in *Cercospora* and *Passalora*. Centraalbureau voor Schimmelcultures, Utrecht, 571 pp.

Dade HA (1940) A revised list of Gold Coast fungi and plant diseases. Bulletin of Miscellaneous Information 1940: 205–247.

Deighton FC (1936a) Preliminary list of fungi and diseases of plants in Sierra Leone and list of fungi collected in Sierra Leone. Bulletin of Miscellaneous Information 1936: 397–424.

Deighton FC (1936b) List of fungi collected in Sierra Leone. Bulletin of Miscellaneous Information 7: 424–433.

Deighton FC (1956) Diseases of cultivated and economic plants in Sierra Leone. Government of Sierra Leone, London.

Deighton FC (1959) Studies on *Cercospora* and allied genera. I. *Cercospora* species with coloured spores on *Phyllanthus* (Euphorbiaceae). Mycological Papers 71: 1–23.

Deighton FC (1969b) Microfungi IV: Some hyperparasitic hyphomycetes and a note on *Cercosporella uredinophila* Sacc. Mycological Papers 118: 1–41; 23 figs. 2 plates.

Deighton FC (1972) Four leaf-spotting hyphomycetes from Africa. Transactions of the British Mycological Society 59: 419–427.

Deighton FC (1973b) Studies in *Cercospora* and allied genera. IV. *Cercosporella* Sacc., *Pseudocercosporella* gen. nov. and *Pseudocercosporidium* gen. nov. Mycological Papers 133: 1–62.

Deighton FC (1974) Studies on *Cercospora* and allied genera. V. *Mycovellosiella* Rangel and a new species of *Ramulariopsis*. Mycological Papers 137: 1–75.

Deighton FC (1976) Studies on *Cercospora* and allied genera. VI. *Pseudocercospora* Speg., *Pantospora* Cif. and *Cercoseptoria* Petr. Mycological Papers 140:1–168.Deighton FC (1979) Studies on *Cercospora* and allied genera VII. New species and redispositions. Mycological Papers 144: 1–56.

Deighton FC (1979) Studies on *Cercospora* and allied genera. VII. New species and redispositions. Mycological Papers 144: 1–56.

Deighton FC (1981) Two species of *Pseudocercospora* from Sierra Leone. Transactions of the British Mycological Society 77: 450–453.

Deighton FC (1983) Studies on *Cercospora* and allied genera. VIII. Further notes on *Cercoseptoria* and some new species and redispositions. Mycological Papers 151: 1–20.

Deighton FC (1987) New species of *Pseudocercospora* and *Mycovellosiella*, and new combinations into *Pseudocercospora* and *Phaeoramularia*. Transactions of the British Mycological Society 88: 365–391.

Deighton FC (1990) A new species of *Cladosporium* causing leaf spots on *Cercestis* in Sierra Leone. Mycological Research 94: 570.

Drouillon R (1951) La maladie des taches brunes de l'arachide. Revue de Mycologie, Supplément Colonial 16: 1–11.

Eboh DO (1981) A taxonomic survey of Nigerian rust fungi: Uredinales Nigerianensis II. Mycologia 73: 445–453.

Ellis MB (1959) *Clasterosporium* and some allied Dematiaceae - *Phragmosporae* II. Mycological Papers 72: 1–75.

Ellis MB (1960) Dematiaceous Hyphomycetes. I. Mycological Papers 76: 1–36.

Ellis MB (1967) Dematiaceous Hyphomycetes. VIII. *Periconiella, Trichodochium*, etc. Mycological Papers 111: 1–46.

Ellis MB (1971) Dematiaceous Hyphomycetes. Commonwealth Mycological Inst, Kew.

Ellis MB (1976, reprinted1985) More dematiaceous Hyphomycetes. Commonwealth Mycological Inst, Kew.

Fomba DC (1984) Rice disease situation in mangrove and associated swamps in Sierra Leone. Tropical Pest Management 30: 73–81.

Harris E (1960) *Ramulispora sorghicola* sp. nov. Transactions of the British Mycological Society 43: 80–84.

Houessou HJH, Beed F, Sikirou R, Ezin V (2011) First report of *Cercospora beticola* on lettuce (*Lactuca sativa*) in Benin. New Disease Reports 23: 16.

Hughes SJ (1952a) Fungi from the Gold Coast I. Mycological Papers 48: 1–91.

Hughes SJ (1953) Fungi from the Gold Coast II. Mycological Papers 50: 1–104.

Kirk PM (1980) *Pseudocercospora abelmoschi*. [Descriptions of Fungi and Bacteria]. International Mycological Institute, Descriptions of Fungi and Bacteria 678.

Kohlmeyer J (1966) Neue Meerespilze an Mangroven. Berichte der Deutschen Botanischen Gesellschaft 79: 27–37.

Kohlmeyer J (1968b) Marine fungi from the tropics. Mycologia 60: 252–270.

Kohlmeyer J, Kohlmeyer E (1971) Marine fungi from tropical America and Africa. Mycologia 63: 831–861.

Koné NA, Koné D, Nicot P (2010) State of knowledge of fungal diversity in Côte d’Ivoire. In: Konate S, Kampmann D (eds) Atlas de la biodiversité de l'Afrique de l'Ouest, Tome III: Côte d'Ivoire. Goethe-Universität Frankfurt am Main, Frankfurt, 172–177 pp.

Kranz J (1964a) Fungi collected in the Republic of Guinea. I. Collection from the rain forest. Sydowia 17: 132–138.

Kranz J (1964b) Fungi collected in the Republic of Guinea. II. Collection from the Kindia area in 1962. Sydowia 17: 174–185.

Kranz J (1966a) Neue *Cercospora* Arten aus Westafrika, Sydowia 19(1-6): 73–83

Kranz, J (1966b) Fungi collected in the Republic of Guinea, III. Collections from the Kindia area in 1963/64, and Host Index. Sydowia 19: 92–107.

Kranz J (1967) Über parasitische Pilzgesellschaften kleinster Areale. Zeitschrift für Pflanzenkrankheiten und Pflanzenschutz / Journal of Plant Diseases and Protection 73: 27–34.

Kranz J (1968) Neue Hyphomyceten aus Guinea. Sydowia 20: 211–217.

Kranz J (1970) Neue *Mycosphaerella*-Arten aus Guinea. Nova Hedwigia 18: 235–239.

Kutama AS, Bashir B, James D (2010) Incidence of *Sorghum* diseases in Dawakin-Kudu Local Government Area, Kano State, Nigeria. African Journal of Agricultural Research 6(4): 307–313.

Leather RI (1959) Diseases of economic plants in Ghana other than cacao. Ghana Ministry of Food and Agriculture Bulletin No. 1, 42 pp.

Lenné JM (1990) A world list of fungal diseases of tropical pasture species. Phytopathological Papers, vol. 31. CAB International, Wallingford, UK.

Lenné JM, Calderón M (1989) Problemas causados por plagas y enfermedades en *Andropogon gayanus*. In: Toledo JM, Vera R, Lascano C, Lenné JM (eds) *Andropogon gayanus* Kunth: Un pasto para los suelos ácidos del trópico. CIAT, pp 191–238.

Luc M (1953a) Champignons graminicoles de Côte d’Ivoire I. - Pyrénomycètes. Revue de Mycologie, Supplément Colonial 18: 1–37.

Marley PS, Diourté M, Neya A, Nutsugah SK, Sérémé P, Katilé SO, Hess DE, Mbaye DF, Ngoko Z (2002) Sorghum and pearl millet diseases in West and Central Africa. In: Leslie JF (ed) Sorghum and millets diseases [based on contributions to the Third Global Conference on Sorghum and Millets Diseases in Guanajuato, Mexico, September 2000], 1st ed. Iowa State Press, Ames, pp 419–425.

Meswaet Y, Mangelsdorff R, Yorou NS, Piepenbring M (2019) A new species of *Pseudocercospora* on *Encephalartos barteri* from Benin. Asian Journal of Mycology 2: 101–109.

Montegut J (1967) Contribution à étude d'un complexe parasitaire s'attaquant à l'appareil aérien des cotonniers et entrainant son déssèchement. Rapport de mission à l’Office du Niger (Mali). Coton et Fibres Tropicales 22: 439–453.

Moreau C (1949) Micromycètes africains. I. Revue de Mycologie, Supplément Colonial 14: 15–22.

Moreau C (1950b) Les mycocécidies des régions tropicales. Revue de Mycologie, Supplément Colonial 15: 1–44.

Moreau C (1952) Les maladies parasitaires des principales cultures coloniales: Revue Bibliographique. X. Revue de Mycologie, Supplément Colonial 17: 84–96.

Moreau C, Moreau M (1951) Pyrénomycètes du caféier en Côte d'Ivoire. Revue de Mycologie, Supplément Colonial 16: 12–80.

Mulder JL (1982) New species and combinations in *Stenella*. Transactions of the British Mycological Society 79: 469–478.

Patouillard N, Hariot P (1900) Enumération des champignons récoltés par M.A. Chevalier au Sénégal et dans le Soudan occidental. In: Morot LM (ed) Journal de botanique. Bureau de journaux, Paris, pp 234–246.

Piątek M, Yorou NS (2018) *Pseudocercospora avicenniicola* on black mangrove (*Avicennia germinans*) in Benin: The first report from Africa. Forest Pathology 49: e12478 (1–4).

Piening LJ (1962) A check list of fungi recorded from Ghana: Part I. Ghana Ministry of Agriculture, Bulletin 2: 1–92.

Resplandy R, Chevaugeon J, Delassus M, Luc M (1954) Première liste annotée de champignons parasites de plantes cultivées en Côte d'Ivoire. Annales de Epiphytes 1: 1–61.

Richardson MJ (1990) An annotated list of seed-borne disease, 4. Edition. International Seet Testing Association, Zürich.

Roger L (1953) Phytopathologie des pays chauds. Tome II. Encyclopédie Mycologique, XVIII, Paris.

Savary S, Bosc J-P, Noirot M, Zadoks J (1988) Peanut rust in West Africa: A new component in a multiple pathosystem. Plant Disease 72: 1001–1009. https://doi.org/10.1094/PD-72-1001.

Sivanesan A (1979) *Mycosphaerella sieberiana* sp. nov. with a *Pseudocercospora* conidial state. Transactions of the British Mycological Society 72: 157–161.

Sivanesan A, Okpala EU (1979) New ascomycetes from Nigeria. Transactions of the British Mycological Society 72: 520–524.

Soura BH, Gnancadja LSA, Koita K, Gnancadja C (2018) Distribution of *Cercospora oryzae*, the fungus causing *Cercospora* leaf spot or narrow brown leaf spot in southern Benin (advantages and constraints of rice production). Asian Journal of Science and Technology 9(11): 8986–8991.

Sydow H, Sydow P (1904) Novae fungorum species. Annales Mycologici 2:162–174.Thomas MD (1991) Development of Gray Leaf Spot on *Sorghum* in Burkina Faso. Plant Disease 75: 45–47.

Thomas MD (1991) Development of Gray Leaf Spot on *Sorghum* in Burkina Faso. Plant Disease 75: 45–47.

Turner PD (1971) Micro-organisms associated with oil palm (*Elaeis guineensis* Jaco.). Phytopathological Papers 14: 1–58.

Viennot-Bourgin G (1959) Étude de micromycètes parasites récoltés en Guinée. Annales de l'Institut National Agronomique 45: 1–91.

Wakefield EM (1918b) Fungi exotici - XXIV. Kew Bulletin 1918: 207–210.

West J (1938) A preliminary list of plant diseases in Nigeria. Bulletin of Miscellaneous Information 1938: 17–23.

Yen J-M (1974) Les *Cercospora* de Côte d'Ivoire - I. Bulletin de la Société Mycologique de France 90: 307–324.

Yen J-M (1975) Les *Cercospora* de Côte d'Ivoire - II. Bulletin de la Société Mycologique de France 91: 89–103.

Yen J-M (1978) Les *Cercospora* de Côte d'Ivoire - III. Bulletin de la Société Mycologique de France 94: 381–383.

Zida PE, Séréme P, Leth V, Sankara P, Somda I, Néya A (2008) Importance of seed-borne fungi of *Sorghum* and pearl millet in Burkina Faso and their control using plant extracts. Pakistan Journal of Biological Science 11: 321–331.
