# Supplementary figures and images for: Unravelling unexplored diversity of cercosporoid fungi (Mycosphaerellaceae, Mycosphaerellales, Ascomycota) in tropical Africa
Source: MycoKeys. 2021 Jun 17;81:69–138. doi: 10.3897/mycokeys.81.67850 (PMC8225595; doi:10.3897/mycokeys.81.67850)

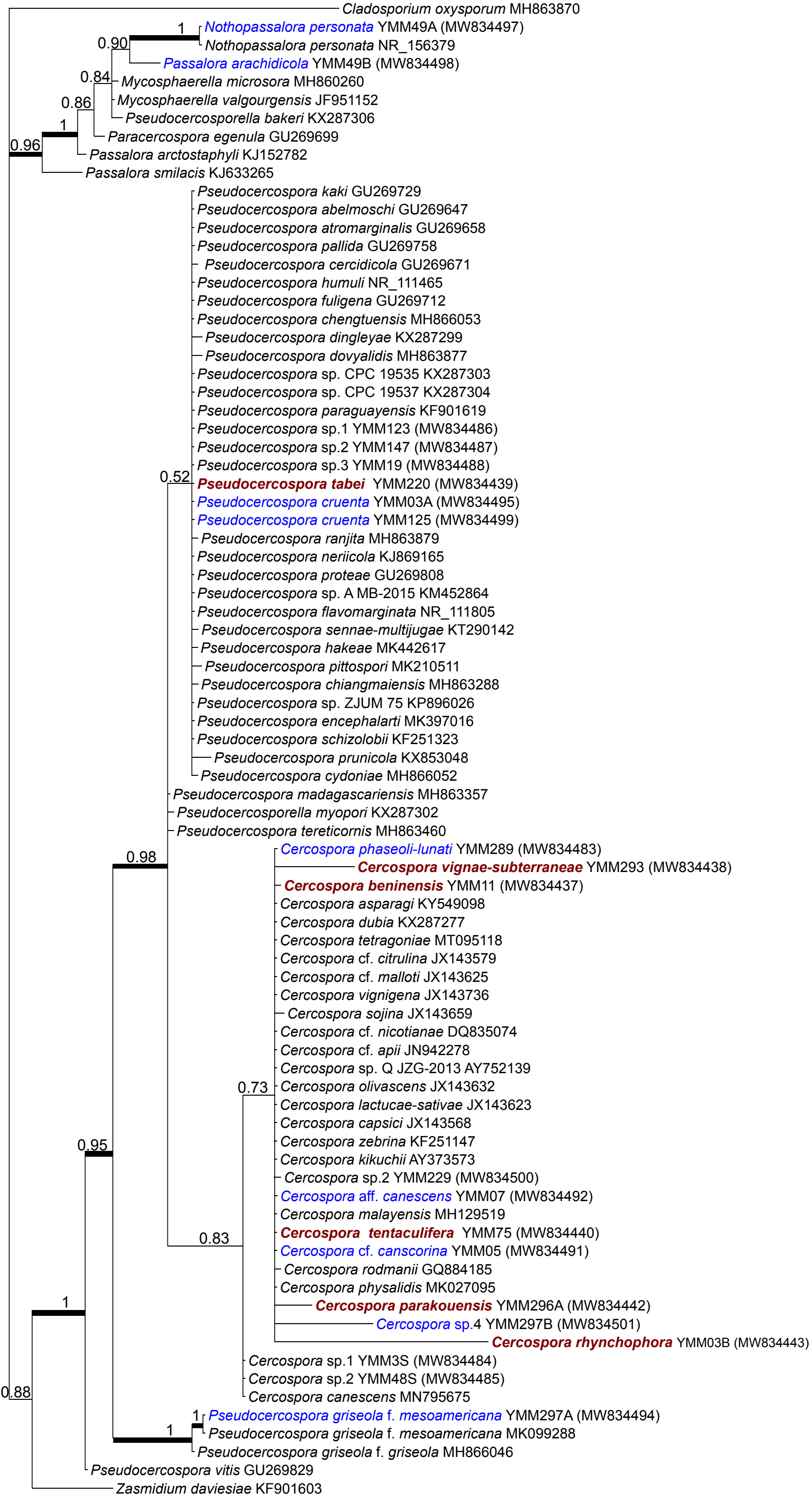

0.04

Supplement: Supplementary material 3 — A Bayesian phylogenetic tree inferred from ITS rDNA sequence data of cercosporoid species [file mycokeys-81-069-s003.pdf]

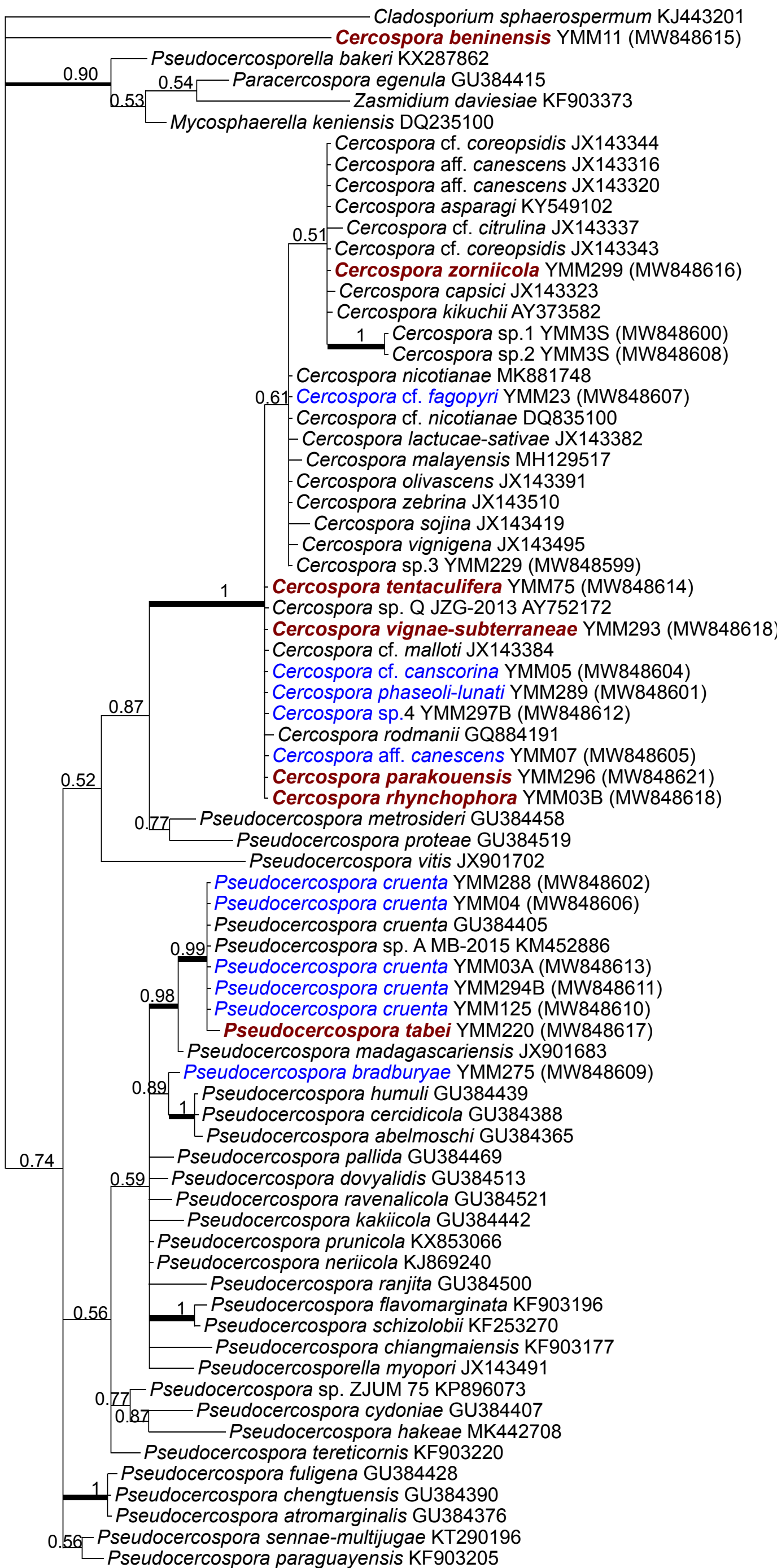

0.09

Supplement: Supplementary material 4 — A Bayesian phylogenetic tree inferred from tef1 DNA sequence data of cercosporoid species [file mycokeys-81-069-s004.pdf]
